# Evolutionary Digital Twin-Oriented Complex Networked Systems driven by node features and the mutation of feature preferences

**DOI:** 10.1371/journal.pone.0303571

**Published:** 2024-05-16

**Authors:** Jiaqi Wen, Bogdan Gabrys, Katarzyna Musial

**Affiliations:** Complex Adaptive Systems, Data Science Institute, University of Technology Sydney, Sydney, NSW, Australia; Rutgers The State University of New Jersey, UNITED STATES

## Abstract

Accurate modelling of complex social systems, where people interact with each other and those interactions change over time, has been a research challenge for many years. This study proposes an evolutionary Digital Twin-Oriented Complex Networked System (DT-CNS) framework that considers heterogeneous node features and changeable connection preferences. We create heterogeneous preference mutation mechanisms to characterise nodes’ adaptive decisions on preference mutation in response to interaction patterns and epidemic risks. In this space, we use nodes’ interaction utilities to characterise the positive feedback from interactions and negative impact of epidemic risks. We also introduce social capital constraint to harness the density of social connections better. The nodes’ heterogeneous preference mutation styles include the (i)inactive style that keeps initial social preferences, (ii) ignorant style that randomly mutates preferences, (iii) egocentric style that optimises individual interaction utility, (iv) cooperative style that optimises the total interaction utilities by group decisions and (v) collaborative style that further allows the cooperative nodes to transfer social capital. Our simulation experiments on evolutionary DT-CNSs reveal that heterogeneous preference mutation styles lead to various interaction and infection patterns. The results also show that (i) increasing social capital enables higher interactions but higher infection risks and uncertainty in decision-making; (ii) group decisions outperform individual decisions by eliminating the unawareness of the decisions of other nodes; (iii) the collaborative nodes under a strict social capital limit can promote interactions, reduce infection risks and achieve higher overall interaction utilities.

## Introduction

Digital Twin (DT) paradigm in the context of Complex Networked Systems (CNSs) means that a network representation is able to accurately reflect or even extend the reality. Over the years, we have seen the trend of developing CNSs with increasing complexity levels that bring us closer and closer to a Digital Twin paradigm. This new research space is called Digital Twin Oriented Complex Networked Systems (DT-CNSs) [1].

Considerable studies in CNS space focus on the simulation and modelling of static networks [2–8]. They decompose the networks as nodes and their connections, which are modelled based on rules that characterise nodes’ preferences for connecting with others. Over the years, three main connection rules were proposed in this space, including preferential attachment [2], homophily [9] and transitivity [10]. More specifically, [2] proposes a scale-free network model based on the preferential attachment to higher node degrees. [9] uses the homophily principle to describe the nodes’ tendency to connect with similarly attributed others. [10] proposes to use transitivity as a critical concept to present nodes’ preferences to connect with their neighbours’ neighbours. The three rules mentioned above have been respectively well analysed and modelled in many studies [11–17]. However, only a few studies consider the combined effect of more than one connection rule in CNS modelling [17, 18]. For example, [18] considers both the homophily and the transitivity effects on a network formation.

Current research on CNSs also progresses towards a more realistic representation of real systems by modelling network topology changes [18–23]. Recent studies also introduce the epidemic spreading processes on dynamic networks and investigate the nodes’ infection occurrences impacted by the evolving network connectivity patterns [24, 25].

In contrast, only a few studies investigate the influence of an epidemic outbreak on network evolution [26, 27]. For example, [26] models the endogenous social distancing phenomenon in evolving social networks by incorporating the interaction risks from an epidemic outbreak into the nodes’ decisions on connecting with others. Despite this study, a heavily underdeveloped research space remains where nodes make heterogeneous decisions on network evolution in response to the connectivity patterns and the infection occurrences for positive feedback. The interrelated changes of networks and processes, motivated by the nodes’ purposeful behavioural changes, add complexity to the CNS dynamics and pose a challenge to the DT-CNS modelling framework.

Some studies have accommodated the individual motivations of social interactions into the individual decision-making processes on connection preferences. They employ the social capital concept to represent people’s investment in social connections for expected returns [28, 29]. For example, in the knowledge-sharing process on social networks, nodes, motivated by reputation and altruism, conduct knowledge-sharing behaviours based on their social capital (such as trust, identification and reciprocity, which are embedded in social relations) [28].

Our previous work proposed the DT-CNS modelling framework based on heterogeneous node features and related preferences, including preferential attachment and homophily [30]. The framework proposed there models CNSs composed of static networks and a dynamic process on the networks. This study extends this framework by modelling the interrelated network evolution and dynamic process on the evolving networks. We build an evolutionary DT-CNS framework featuring heterogeneous node features and changeable connection preferences, which combine the effects of preferential attachment, homophily and transitivity. We propose to measure the nodes’ interaction utilities based on the reward (i.e. social stimuli originates from and motivates the interactions) and epidemic risks from their interactions. We create heterogeneous preference mutation mechanisms to characterise nodes’ adaptive decisions on changing the connection preferences in response to the changes of interaction utilities. In addition, we introduce the nodes’ maximum investment in social connections, referred to as social capital constraints, to the nodes’ preference mutation process and propose to model nodes’ preference changes as an optimisation process for higher interaction utilities with limited social connections. Under the social capital limit, nodes can follow heterogeneous preference mutation styles, including the (i) inactive style that keeps zero social preferences, (ii) ignorant style that randomly mutates preferences, (iii) egocentric style that optimises individual interaction utility, (iv) cooperative style that optimises the total interaction utilities by group decisions and (v) collaborative style that further allows the cooperative nodes to transfer social capital.

Through conducting simulation-based experiments on the evolutionary DT-CNSs, we find that (i) increasing social capital enables higher interactions and triadic closures but higher infection risks and uncertainty in decision-making; (ii) group decisions outperform individual decisions by avoiding the unawareness of the decisions of other nodes; (iii) the collaborative strategy, given a strict social capital limit, outperforms the inactive, ignorant, egocentric and cooperative strategies by achieving more interactions and triadic closures, lower infection numbers and consequently, higher interaction utilities.

This study innovates in the following aspects:

- proposal of an evolutionary DT-CNS framework related to heterogeneous node features and the changeable preferences for connecting with others.
- introduction of social capital limits to nodes’ adaptive decisions on preferences changes for interaction utility maximisation.
- creation of heterogeneous preference mutation mechanisms that drive nodes’ individual and group behaviours.
- suggestion of strict social capital limit on social connections and collaboration between nodes in an epidemic outbreak.

The rest of this study is structured as follows: Modelling Framework presents the methodology of building an evolutionary DT-CNS. Following this, Experiment Design, Results and Analysis builds and evaluates the evolutionary DT-CNSs. Finally, we conclude with Conclusion.

## Modelling framework

In this section, we build an evolutionary DT-CNS based on (i) heterogeneous features of nodes, (ii) nodes’ preferences for connecting with others and (iii) heterogeneous preference mutation styles that are driven by the utility and the risk of interactions. We present the components of DT-CNSs related to nodes, their interactions driven by features and related preferences, and their conditions in an epidemic spread in Complex Networked System Components and propose ways to calculate the interaction utilities and risks for individuals. In CNS Dynamics, we model the dynamics of DT-CNS that governs the nodes’ interactions and interaction preference mutation based on their utilities and risks.

### Complex Networked System Components

CNSs are composed of the (i) nodes that possess features and preferences for connecting with others and (ii) the edges that represent interactions between pairs of nodes. These nodes and edges compose the CNSs’ network dimension, and in the CNSs’ process dimension, the process (e.g. epidemic) spreads via the edges and results in changes in the nodes’ status (e.g. nodes’ health conditions). The network and process dimensions constitute the CNS.

We represent the network at time *t* as **G**_*t*_ = {**V**_*t*_, **E**_*t*_}, based on the dynamic network components: nodes **V**_*t*_ = {*v*_1,*t*_, ⋯, *v*_*N*,*t*_} and edges **E**_*t*_ = {*e*_*ij*,*t*_|*v*_*i*,*t*_, *v*_*i*,*t*_ ∈ **V**_*t*_}. The node *v*_*i*,*t*_ at time *t* is defined as:

$$\begin{array}{c} v_{i,t}\left(f_{i,t},sDNA_{i,t},\beta_{i,t},U_{i,t},R_{i,t},{\$}_{i,t},{\hat{\$}}_{i,t}\right) \end{array}$$ (1)

where we represent the node’s features and the related preferences with **f**_*i*,*t*_ and *sDNA*_*i*,*t*_. *β*_*i*,*t*_ represents the node’s health condition with 0 and 1, each representing the node’s healthy and infected condition. The node *v*_*i*,*t*_ obtains the utility U_*i*,*t*_ from interacting with others at time *t* and suffers the interaction risk R_*i*,*t*_ given an epidemic outbreak. The social capital embedded in nodes’ interactions is denoted by $_*i*,*t*_ and constrained by the social capital limit ${\hat{\$}}_{i,t}$. Nodes interact under the social capital limit ${\hat{\$}}_{i,t}$ for objectives related to interaction utilities.

The node’s preferences *sDNA*_*i*,*t*_ for the related features **f**_*i*,*t*_ is determined by

$$\begin{array}{c} sDNA_{i,t}=\left({\text{p}}_{i,t},{\text{w}}_{i,t}^{p},{\text{h}}_{i,t},{\text{w}}_{i,t}^{h},c_{i,t},w_{i,t}^{c}\right) \end{array}$$ (2)

where **p**_*i*,*t*_ and **h**_*i*,*t*_ are vectors that determine the negative, zero, and positive preferences for features and feature differences by values −1, 0 and 1. These two vectors are followed with the same length weighting vectors **w**^*p*^ and **w**^*h*^, indicating the weight of preference and valuing within (0, 1]. Similarly, c determines the negative, zero and positive preference for connecting with nodes with common friends by −1, 0 and 1, followed by the weight of preference $w_{i,t}^{c}$.

The edge *e*_*ij*,*t*_ represents the interactions between the node pair: *v*_*i*,*t*_ and *v*_*j*,*t*_, which is defined as:

$$\begin{array}{c} e_{ij,t}\left(I_{ij,t},w_{ij,t}^{I}\right) \end{array}$$ (3)

where *I*_*ij*,*t*_ represents no interaction and an interaction between a node pair with 0 and 1 respectively. It is followed by an interaction intensity factor $w_{ij,t}^{I}$, ranging beteween 0 and 1.

#### Interaction reward

The interaction reward generally refers to social stimuli that positively reinforce the frequency or intensity of a behaviour pattern [31, 32]. In the context of social networks, the interaction reward **R**_*i*,*t*_ for node *v*_*i*,*t*_ represents the social stimuli such as cultural goods, money, positive emotional expressions and social reputation, which originates from and further motivates the interactions between node *v*_*i*,*t*_ and others.

In this study, we assume that a node’s interaction reward depends on the scores and frequency of its interactions. It is calculated by aggregating the reward *r*_*i*,*t*_(*i*, *j*) from any pairwise interactions.

$$\begin{array}{c} {\text{R}}_{i,t}=\sum_{v_{j,t}\in V_{t}}r_{i,t}\left(i,j\right) \end{array}$$ (4)

The reward for node *v*_*i*,*t*_ from the interaction with node *v*_*j*,*t*_ depends on the interaction score *Π*_*ij*,*t*_ and the individual interaction cost *c*_*i*,*t*_.

$$\begin{array}{c} r_{i,t}\left(i,j\right)=\epsilon \Pi_{ij,t}-\psi_{i,t}^{\beta }c_{i,t} \end{array}$$ (5)

where *ϵ* > 0 indicates that the reward correlates directly with the interaction score Π_*ij*,*t*_. *ψ* ∈ (1, 2] is a tuning parameter for the node’s average interaction cost *c*_*i*,*t*_, depending on the node’s infection status *β*_*i*,*t*_. The infected nodes have a higher interaction cost and low-score interactions.

The interaction score Π_*ij*,*t*_ of the interaction between the node pair *v*_*i*,*t*_ and *v*_*j*,*t*_ depends on their mutual evaluation based on features and related preferences.

$$\begin{array}{c} \Pi_{ij,t}=\frac{1}{2}\left(\Pi_{i\to j,t}+\Pi_{j\to i,t}\right) \end{array}$$ (6)

where Π_*i*→*j*,*t*_ and Π_*j*→*i*,*t*_ each represents the unilateral evaluation of node *v*_*i*,*t*_ and node *v* + *j*, *t* on their interaction.

The score Π_*i*→*j*_ that node *v*_*i*,*t*_ assigns to its interaction with *v*_*j*,*t*_ is calculated based on the preferential attachment, homophily and closure effects, each represented as $\pi_{i\to j,t}^{h}$, $\pi_{i\to j,t}^{p}$ and $\pi_{i\to j,t}^{c}$.

$$\begin{array}{c} \Pi_{i\to j,t}=\{\begin{array}{cc} \frac{1}{3}\left(\pi_{i\to j,t}^{h}+\pi_{i\to j,t}^{p}+\pi_{i\to j,t}^{c}\right)+\epsilon_{i\to j,t} & \text{if}\;v_{i,t}\;\text{and}\;v_{j,t}\;\text{encounters}.\\ 0 & \text{if}\;\text{else}. \end{array} \end{array}$$ (7)

where *v*_*i*,*t*_ evaluates the pairwise interaction with others by averaging the homophily score $\pi_{i\to j,t}^{h}$, preferential attachment score $\pi_{i\to j,t}^{p}$ and the closure effect $\pi_{i\to j,t}^{c}$ based on their encounters, ranging between [0, 1]. The nodes encounter each other based on an encounter rate at *η*, where the nodes’ encounters follow a binary distribution and *η* denotes the probability of encounters. Moreover, without an encounter, the subjective node assigns 0 to this unilateral evaluation of the pairwise interactions. *ϵ*_*i*→*j*,*t*_ ∼ *N*(0, 0.01^2^) is a random interference that follows a normal distribution *N*(0, 0.01^2^). It characterises the unobservable and uninterpretable interference in nodes’ evaluation on interactions between pairs of nodes, which results in different node pair scores even for the node pairs attributed with the same features.

The homophily effect $\pi_{i\to j,t}^{h}$ describes the node’s preference for connecting with similar or dissimilar others. It is calculated based on the feature differences and the preference vectors **p**_*i*,*t*_ and ${\text{w}}_{i,t}^{p}$:

$$\begin{array}{c} \pi_{i\to j,t}^{h}=\frac{1}{2l}{\left|{\text{f}}_{i}-{\text{f}}_{j}\right|}^{\tau }\left({\text{h}}_{i,t}⊙{\text{w}}_{i,t}^{h}\right)+\frac{1}{2} \end{array}$$ (8)

where the feature vector **f**_*i*_ for node *v*_*i*,*t*_ and the feature vector **f**_*j*_ for node *v*_*j*,*t*_ include the standardised feature values from a range [0, 1]. |**f**_*i*_ − **f**_*j*_| represents the elementary absolute values of the difference between the feature vector **f**_*i*_ and **f**_*j*_. *τ* denotes the transpose operator. **h**_*i*,*t*_ represents the preference vector of node *v*_*i*,*t*_ for the feature differences. ${\text{w}}_{i,t}^{h}$ represents the corresponding preference weighting vector. ⊙ is the Hadamard product operator. The aggregated homophily effects from each feature difference are averaged and then standardised based on the feature-length *l*, ranging between [0, 1].

The preferential attachment effect $\pi_{i\to j,t}^{p}$ describes the node’s preference for connecting with other nodes with specific features:

$$\begin{array}{c} \begin{array}{c} \pi_{i\to j,t}^{p}=\frac{1}{2l}{\text{f}}_{j}^{\tau }\left({\text{p}}_{i,t}⊙{\text{w}}_{i,t}^{p}\right)+\frac{1}{2} \end{array} \end{array}$$ (9)

where **f**_*j*_ represents the feature vector for ndoe *v*_*j*,*t*_. **p**_*i*,*t*_ represents the preference vector of node *v*_*i*,*t*_ for the features. ${\text{w}}_{i,t}^{p}$ represents and the corresponding weighting factor. Similar to the homophily effect, each feature’s aggregated preferential attachment effects are also standardised with the feature-length *l*, ranging between [0, 1].

The closure effect $\pi_{i\to j,t}^{c}$ describes the node’s preference for connecting with nodes with or without common friends.

$$\begin{array}{c} \begin{array}{c} \pi_{i\to j,t}^{c}=c_{i,t}w_{i,t}^{c}\frac{\sum_{v_{k}\in \text{V}}I_{ik,t}I_{jk,t}}{\sum_{v_{k}\in \text{V}}I_{ik,t}} \end{array} \end{array}$$ (10)

where we calculate the number of common friends between node *v*_*i*,*t*_ and node *v*_*j*,*t*_ with $\sum_{v_{k}\in \text{V}}I_{ik,t}I_{jk,t}$. The proportion of common friends with node *v*_*j*,*t*_ from the perspective of node *v*_*i*,*t*_ is calculated as $\frac{\sum_{v_{k}\in \text{V}}I_{ik,t}I_{jk,t}}{\sum_{v_{k}\in \text{V}}I_{ik,t}}$.

#### Interaction risk

Given an epidemic outbreak, the node *v*_*i*,*t*_, either healthy or sick, takes the risk of getting infected or infecting the neighbours via interactions. We describe this epidemic spreading process with the “susceptible-infected-recovered” (*SIR*) model and assume an epidemic spreads from a single node and propagates with a transmissibility rate at $\zeta_{t}^{s}$ and a recovery rate at $\zeta_{t}^{r}$ every time an interaction occurs. The node *v*_*i*,*t*_ gets infected at a probability of ${\text{pr}}_{t}^{s}=\zeta_{t}^{s}$ given a single interaction with an infected node and, once infected, recovers at a probability of ${\text{pr}}_{t}^{\phi }\equiv \zeta_{t}^{r}$ before another interaction occurs.

We measure the infection risk $R_{i,t}^{H}$ of a healthy node *v*_*i*,*t*_ at time *t* with its probability of getting infected based on the available information about interactions and node infections.

$$\begin{array}{c} R_{i,t}^{H}=1-\prod_{v_{j,t}\in V_{t}}{\left(1-pr^{s}\right)}^{I_{ij,t}\beta_{j,t}} \end{array}$$ (11)

where $\prod_{v_{j,t}\in V_{t}}{\left(1-pr^{s}\right)}^{I_{ij,t}\beta_{j,t}}$ represents the probability of keeping healthy given the interactions *I*_*ij*,*t*_ with the nodes neighbour and the neighbour’s infected condition *β*_*j*,*t*_ = 1.

Similarly, we measure the spreading risk $R_{i,t}^{I}$ of an infected node *v*_*i*,*t*_ at time *t* with its probability of infecting others via interactions.

$$\begin{array}{c} R_{i,t}^{I}=1-\prod_{v_{j,t}\in V_{t}}{\left(1-pr^{s}\right)}^{I_{ij,t}\left(1-\beta_{j,t}\right)} \end{array}$$ (12)

where $\prod_{v_{j,t}\in V_{t}}{\left(1-pr^{s}\right)}^{I_{ij,t}(1-\beta_{j,t}})$ represents the probability of keeping healthy given the interactions *I*_*ij*,*t*_ with the node’s neighbour and the neighbour’s healthy condition *β*_*j*,*t*_ = 0.

#### Interaction utility

Referring to [26], we calculate the interaction utility U_*i*,*t*_ of the node *v*_*i*,*t*_ based on the interaction reward and the risk avoidance.

$$\begin{array}{c} U_{i,t}=\{\begin{array}{cc} R_{i,t}\left(1-R_{i,t}^{H}\right) & \text{if}\;\beta_{i,t}=1.\\ \delta R_{i,t}\left(1-R_{i,t}^{I}\right) & \text{if}\;\beta_{i,t}=0. \end{array} \end{array}$$ (13)

where the utility for node *v*_*i*,*t*_, given its healthy condition *β*_*i*,*t*_ = 1, is dependent on the infection risk $R_{i,t}^{H}$ and the interaction reward $R_{i,t}$. In contrast, in the infected condition, the node *v*_*i*,*t*_’s utility is directly correlated with interaction reward $R_{i,t}$, excluding the spreading risk $R_{i,t}^{I}$ and depreciated by *δ* due to the infected condition.

The expected interaction utility EU_*i*,*t*_ depends on the interaction probability, recovery probability and the presumed behavioural changes at *t*. We denotes the interaction utility based on the node’s health status, $U_{i,t}^{H}$ for a healthy node and $U_{i,t}^{I}$ for an infected node. The expected interaction utility is calculated as

$$\begin{array}{c} EU_{i,t}=\{\begin{array}{cc} U_{i,t}^{H}\left(1-R_{i,t-1}^{H}\right)+U_{i,t}^{I}\left(R_{i,t-1}^{H}\right) & \text{if}\;\beta_{i,t-1}=1.\\ U_{i,t}^{I}\left(1-{\text{pr}}^{\phi }\right)+U_{i,t}^{H}{\text{pr}}^{\phi } & \text{if}\;\beta_{i,t-1}=0. \end{array} \end{array}$$ (14)

where $R_{i,t-1}^{H}$ and pr^*ϕ*^ each describes the probability of this node getting infected and recover over time, given the interaction structures at *t* − 1. $U_{i,t}^{H}$ and $U_{i,t}^{I}$ each represents the individual utility calculated by each node based on their changes of preferences and the available information of the other nodes.

### CNS dynamics

We model the network dynamics based on the interaction scores driven by the features and related preferences. The interaction scores evolve by the preference mutation in response to an epidemic outbreak towards positive feedback related to the individual utility U_*i*,*t*_. We present the network growth and its evolution process in Network formation and Network evolution.

#### Network formation

The unconnected nodes encounter and simultaneously interact based on the expectations of interaction reward and interaction risks under social capital constraints. The social capital constraint refers to people’s maximum investment, due to personal capacity or social restrictions, in social interactions for expected returns [28, 29]. The interaction between a node pair is determined by

$$\begin{array}{c} I_{ij,t}=\{\begin{array}{cc} 1 & \text{if}\;r_{i,t}\left(i,j\right),r_{j,t}\left(i,j\right)\geq 0,{\$}_{i,t}<{\hat{\$}}_{i,t}\;\text{and}\;{\$}_{j,t}<{\hat{\$}}_{j,t}.\\ 0 & \text{if}\;\text{else}. \end{array} \end{array}$$ (15)

where nodes *v*_*i*,*t*_ and *v*_*j*,*t*_ interact under the social capital constraints ${\hat{\$}}_{i,t}$ and ${\hat{\$}}_{j,t}$ for a beneficial interaction reward *r*_*i*,*t*_(*i*, *j*). $_*i*,*t*_ and $_*j*,*t*_ each denotes the social capital of node *v*_*i*,*t*_ and node *v*_*j*,*t*_ They tend to use social capital best and select the most promisingly beneficial interactions characterised by higher interaction rewards.The interaction intensity of this node pair also depends on the interaction score:

$$\begin{array}{c} w_{ij,t}^{I}=\{\begin{array}{cc} \Pi_{ij,t} & \text{if}\;I_{ij,t}=1.\\ 0 & \text{if}\;I_{ij,t}=0. \end{array} \end{array}$$ (16)

#### Network evolution

The networks evolve with the changes in social relations and people’s preferences related to features. In this section, we introduce a preference mutation mechanism while incorporating five mutation styles: (i) inactive mutation where nodes keep initial preferences, (ii) ignorant mutation where nodes randomly mutate preferences, (iii) egocentric mutation that maximises the respective individual utility while unaware of others’ mutation actions, (iv) cooperative mutation where bonded nodes cooperate to maximise their overall utilities and (v) collaborative mutation that enables the nodes to transfer social capital and cooperate.

**Inactive** style describes the nodes’ inaction to the changes of interaction utilities and risks. These nodes keep their initial social preferences and make social contact by calculating the interaction scores for node pairs under their respective social capital constraints.

**Ignorant** style describes the active nodes who are unaware of the individual utilities and thus randomly select their social preferences related to preferential attachment, homophily and closure effects between positive, zero and negative.

**Egocentric** nodes optimise their interaction utilities by social preference mutation shortly after interactions while aware of others’ social preferences and, at the moment, assuming others’ inactiveness in preference mutation. Each egocentric node can evaluate *γ* sets of random preference changes in what-if scenarios related to the respective preference change and selects the best-performing one to achieve the highest interaction utility. The individual optimisation process is determined as follows:

$$\begin{array}{c} \begin{array}{cc} & \underset{{\text{sDNA}}_{i,t}}{\text{max}}\;\;EU_{i,t}\\ & s.t.\;0\leq {\$}_{i,t}\leq {\hat{\$}}_{i,t} \end{array} \end{array}$$ (17)

where **sDNA**_*i*,*t*_ represents the set of preference vectors for node *v*_*i*,*t*_ at time *t*. EU_*i*,*t*_ represents the expected utility of node *v*_*i*,*t*_ at time *t*. $_*i*,*t*_ represents the social capital of node *v*_*i*,*t*_ at time *t*. ${\hat{\$}}_{i,t}$ represents the corresponding social capital constraint for node *v*_*i*,*t*_.

**Cooperative** nodes cooperate and make a group decision on preference changes to maximise their total interaction utilities. In this study, we assume that all nodes in the node set **V**_*t*_ at time *t* cooperate with each other. These cooperative nodes consider *γ* attempts in preference mutation, and to evaluate these attempts, conduct what-if analyses under the social capital constraints of each node. The decision making process is shown as follows:

$$\begin{array}{c} \begin{array}{cc} & \underset{\left\{{\text{sDNA}}_{i,t}\right\}}{\text{max}}\;\;\sum_{v_{i,t}\in {\text{V}}_{t}}EU_{i,t}\\ & s.t.\;0\leq {\$}_{i,t}\leq {\hat{\$}}_{i,t} \end{array} \end{array}$$ (18)

where **sDNA**_*i*,*t*_ represents the set of preference vectors for node *v*_*i*,*t*_ at time *t*. EU_*i*,*t*_ represents the expected utility of node *v*_*i*,*t*_ at time *t*. $\sum_{v_{i,t}\in {\text{V}}_{t}}EU_{i,t}$ represents the sum of the expected utility of all nodes in the node set **V**_*t*_ at time *t*. $_*i*,*t*_ represents the social capital of node *v*_*i*,*t*_ at time *t*. ${\hat{\$}}_{i,t}$ represents the corresponding social capital constraint for node *v*_*i*,*t*_.

**Collaborative** nodes collaborate in a group decision on their preference changes. In contrast with the decision-making of the cooperative nodes under the respective individual social capital constraints, collaborative nodes can transfer their social capital between each other, where they make a group decision under the overall social capital constraints. The decision-making process of collaborative nodes is shown as follows:

$$\begin{array}{c} \begin{array}{cc} & \underset{\left\{{\text{sDNA}}_{i,t}\right\}}{\text{min}}\;\;\sum_{v_{i,t}\in {\text{V}}_{t}}EU_{i,t}\\ & s.t.\;0\leq \sum_{v_{i,t}\in {\text{V}}_{t}}{\$}_{i,t}\leq \sum_{v_{i,t}\in {\text{V}}_{t}}{\hat{\$}}_{i,t} \end{array} \end{array}$$ (19)

where **sDNA**_*i*,*t*_ represents the set of preference vectors for node *v*_*i*,*t*_ at time *t*. EU_*i*,*t*_ represents the expected utility of node *v*_*i*,*t*_ at time *t*. $\sum_{v_{i,t}\in {\text{V}}_{t}}EU_{i,t}$ represents the sum of the expected utility of all nodes in the node set **V**_*t*_ at time *t*. $_*i*,*t*_ represents the social capital of node *v*_*i*,*t*_ at time *t*. ${\hat{\$}}_{i,t}$ represents the corresponding social capital constraint for node *v*_*i*,*t*_.

#### Epidemic transmission with recovery

The epidemic spreads from the seed nodes **S**_*t*_ = {⋯, *v*_*i*,*t*_, ⋯|*v*_*i*,*t*_ ∈ *V*_*t*_}, propagates with the transmissibility rate at $\zeta_{t}^{s}$ and a recovery rate at $\zeta_{t}^{r}$. As presented in Interaction risk, a healthy node, given a connection with an infected node, gets infected at the probability of ${\text{pr}}^{s}=\zeta_{t}^{s}$. Correspondingly, the probability of a healthy node *v*_*i*,*t*_ (*β*_*i*,*t*−1_ = 0) keeping healthy (*β*_*i*,*t*_ = 0) can be calculated as

$$\begin{array}{c} pr\left(\beta_{i,t}=1|\beta_{i,t-1}=0\right)=\prod_{v_{j,t}\in V_{t}}{\left(1-pr_{t}^{s}\right)}^{I_{ij,t}\beta_{j,t}} \end{array}$$ (20)

which value depends on the epidemic transmissibility ${\text{pr}}_{t}^{s}=\zeta_{t}^{s}$, the interactions *I*_*ij*,*t*_ with each neighbour and the neighbour’s infected condition *β*_*j*,*t*_ = 1.

In contrast, the recovery rate *ζ*^*r*^ denotes the probability of the recovery of any infected node despite of the connections with infected others. The probability of an infected node *v*_*i*,*t*_ (*β*_*i*,*t*−1_ = 1) recovering to be healthy (*β*_*i*,*t*_ = 1) can be calculated as

$$\begin{array}{c} pr\left(\beta_{i,t}=0|\beta_{i,t-1}=1\right)\equiv \zeta^{r} \end{array}$$ (21)

where the recovery probability keeps equal to the recovery rate at *ζ*^*r*^.

## Experiment design, results and analysis

In this section, we conduct simulation-based experiments with the proposed DT-CNS and investigate the impact of different social capital constraints and heterogeneous preference mutation styles on the evolving interaction and infection patterns. Initial Parameters illustrates the initialised parameters of the DT-CNSs and the backbone networks of network simulations. Interaction Patterns and Infection Patterns present the interaction and infection patterns in different interaction scenarios. We calculate and compare the infection utilities in Interaction Utilities based on the interaction and infection patterns.

### Initial parameters

We simulate a 30-node network interacting in an epidemic outbreak for 100 iterations based on their interaction utilities related to preferences for their respective features and epidemic risks. Table 1 shows the initial parameters related to the nodes’ features, preferences, and epidemic transmissibility.

**Table 1:** The initialised parameters for the DT-CNSs.

CNS Component	Parameter	Meaning	Value
Network	*i*	Node identity	[0, 1, 2, ⋯, 29]
	**f**	Feature	U[0,1]
	**p**	Preference for features	[0]
	**w** ^*p*^	Weights of preference for features	[1]
	**h**	Preference for feature differences	[0]
	**w** ^*h*^	Weights	[1]
	**c**	Preference for transitivity	[0]
	**w** ^*c*^	Weights of preference for transitivity	[1]
	*η*	Encounter rate	1.00
	u^0^	Initial interaction cost	0.40
	*η*_*i*,*t*_	Infection penalty	1.5
	*γ*	Length of action space per node	2
	*δ*	Interaction utility discount	0.01
	*ϵ*	Scaling parameter for interaction utility	1.00
Process	*η* ^*s*^	Infection rate	0.20
	*η* ^*r*^	Recover rate	0.20

As shown in Table 1, the 30 nodes are each denoted by integer values ranging from 0 to 29. They have a single feature simulated by a random uniform distribution within [0, 1]. In this study, we select the 30 nodes with a single uniform feature as an example of the social network simulations to illustrate the mutual impact of epidemic spread and the network evolution. In the initialisation stage, these nodes are connected with each other based on the backbone network. These nodes have zero preferences regarding preferential attachment, homophily and closure effects. These preferences are initialised for illustration purposes, which mutate in the network evolution process in response to the feedback of interactions and infections. The corresponding preference weights keep fixed at 1.

Based on these features and preferences, the nodes encounter each other given an encounter rate of *η* = 1 and evaluate the node pairs based on the interaction scores. The interaction cost initialised for the healthy nodes keeps at 0.4, which turns into 0.6 under the infection penalty of *η* = 1.5 when the nodes get infected.

Given the above parameters initialised above, we investigate network evolution that starts from different backbone networks. We consider three scenarios with different set-ups of the backbone networks, including (i) an unconnected network composed of 30 nodes without connections, (iii) a scale-free network driven by a 0.2 probability of connecting with others, composed of 30 nodes and 56 edges, and (iii) a random network composed of 30 nodes and 56 random connections with each other. In each scenario, the epidemic spread from the most popular nodes, characterised by the highest node degrees. We respectively consider the first node, the first two nodes and the first three nodes that rank high in popularity as the seed nodes for the epidemic spread. In an empty network, the seed nodes are randomly selected as all node degrees are zero. Under the influence of seed selection and backbone networks, the networks evolve by preference mutation and re-evaluation of the pairwise interactions. We generate 8 simulations of evolving social networks in each experiment scenario and investigate the variability of infection patterns and interaction patterns in the following subsections.

### Infection patterns

**Fig 1:**
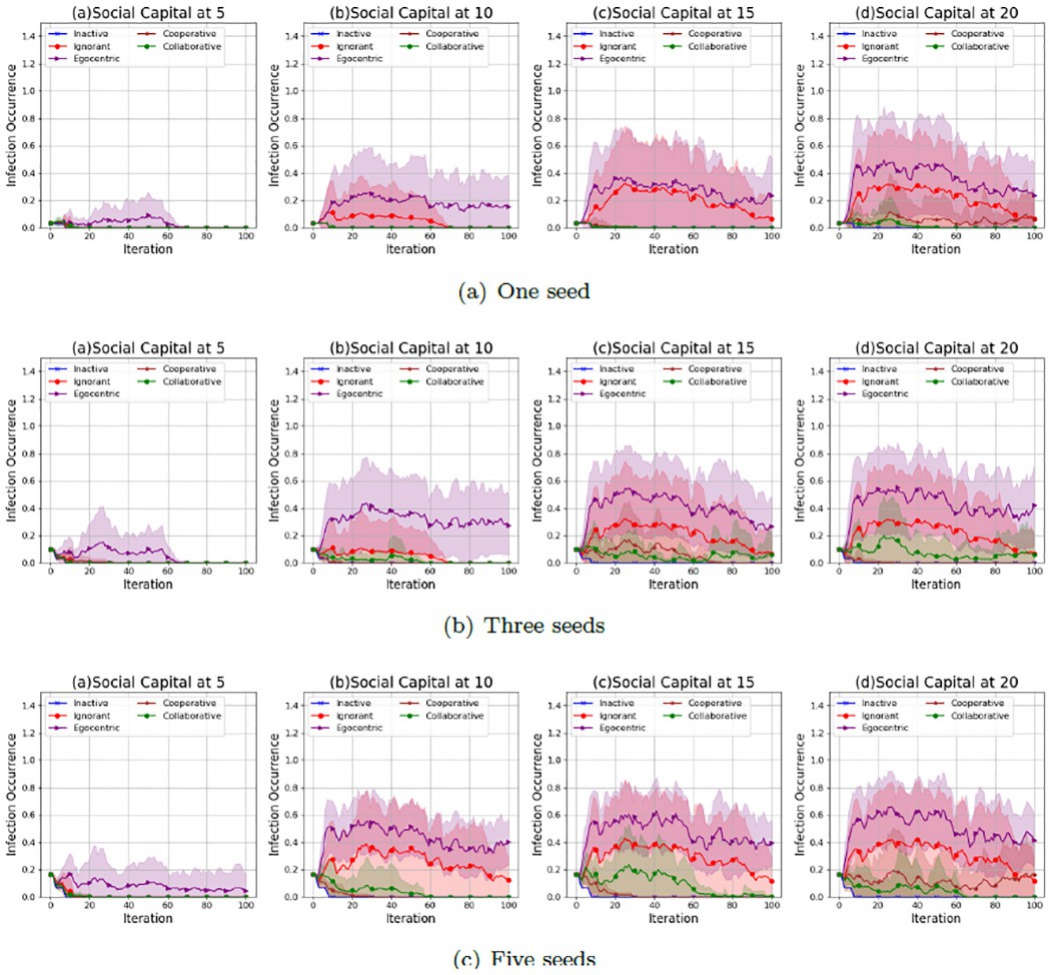
The average values and standard deviations of infection occurrences in 100 iterations in the evolving social networks based on an unconnected backbone network under the impact of one seed (a), two seeds (b) and three seeds (b). In each scenario, the evolving social networks are respectively driven by inactive, ignorant, egocentric, cooperative and collaborative mutation styles.

**Fig 2:**
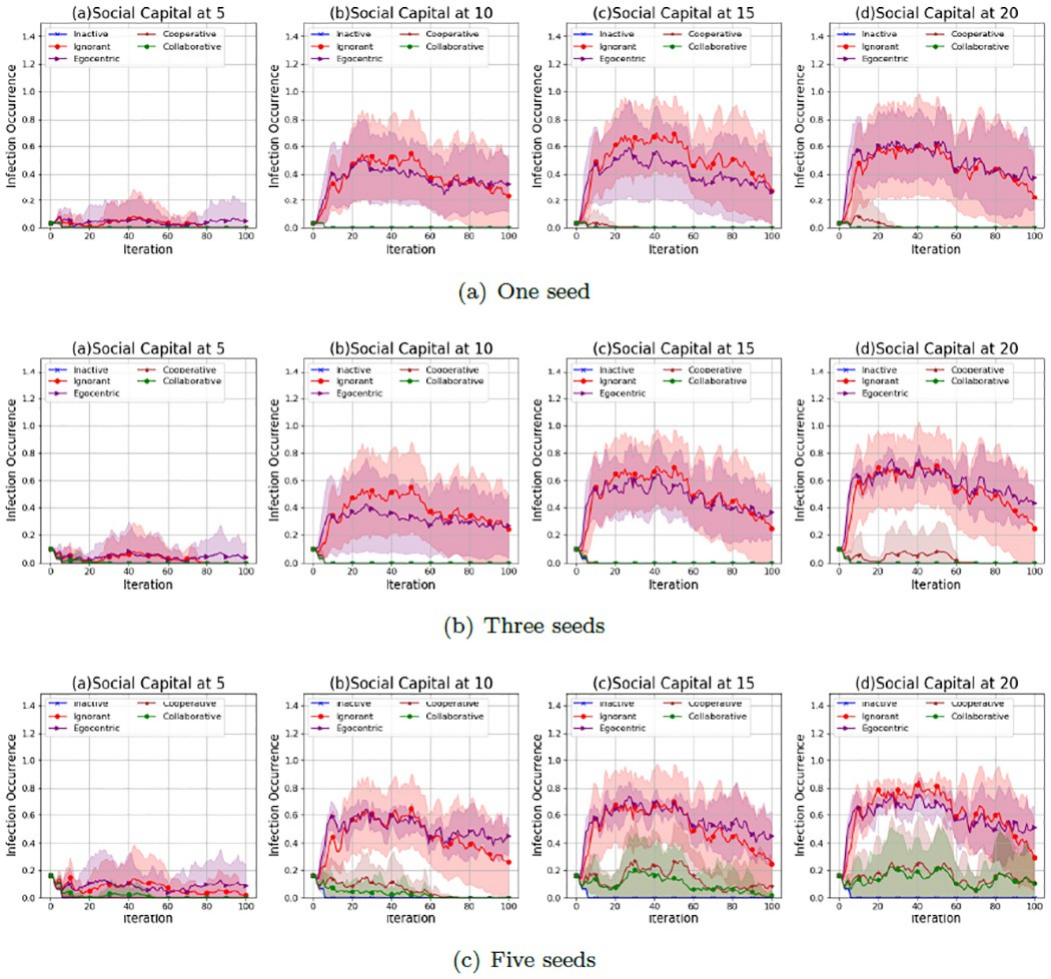
The average values and standard deviations of infection occurrences in 100 iterations in the evolving social networks based on a random backbone network under the impact of one seed (a), two seeds (b) and three seeds (b). In each scenario, the evolving social networks are respectively driven by inactive, ignorant, egocentric, cooperative and collaborative mutation styles.

**Fig 3:**
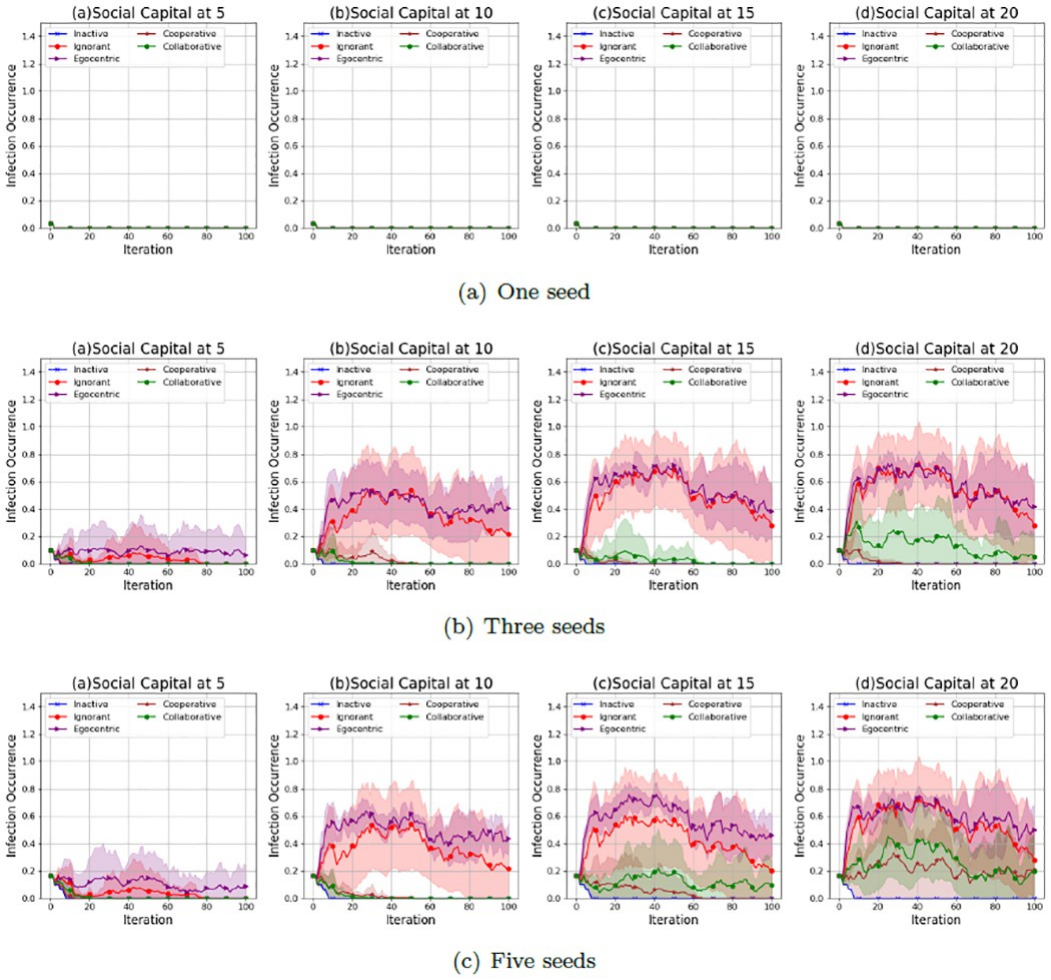
The average values and standard deviations of infection occurrences in 100 iterations in the evolving social networks based on a scale-free backbone network under the impact of one seed (a), two seeds (b) and three seeds (b). In each scenario, the evolving social networks are respectively driven by inactive, ignorant, egocentric, cooperative and collaborative mutation styles.

We investigate the infection patterns with a mutual influence on the evolving social networks according to the infection occurrence (proportion of infected nodes to the overall 30 nodes) in the evolving social networks that starts from the unconnected backbone network (See Fig 1), the random backbone network (See Fig 2) and the scale-free backbone network (See Fig 3).

As shown in Fig 1, the infection occurrence in evolving social networks increases with the social capital limit and the number of seeds in the epidemic spreading process. This is because higher social capital limits and more seeds lead to more interactions with the infected nodes, increasing infection risks. The infection occurrences in networks driven by the egocentric and ignorant nodes are much higher, with larger standard deviations, than in networks driven by the other types of nodes. In addition, the infection occurrences of egocentric nodes are highest among all the node types. This indicates that maximising individual reward by individual decisions without considering others’ rewards can lead to higher infection occurrences. The inactive nodes keep healthy with an infection occurrence close to zero for 100 iterations in all experiment scenarios. This is because inactive nodes keep zero preferences without active interactions with others. The infection occurrence of cooperative and collaborative nodes is close to zero, with smaller standard deviations given one seed or three seeds in epidemic spread. In contrast, the infection occurrence of cooperative and collaborative nodes is positive, given five seeds in epidemic spread. This indicates that cooperation and collaboration between nodes have fewer infection risks than other nodes. Cooperation and collaboration, given a lower social capital and fewer seeds, can mitigate the infections to zero.

As shown in Fig 2, the infection occurrences in the evolving social networks given a random backbone network have similar patterns to that of evolving social networks given an unconnected backbone network. The infection occurrence increases with the social capital limit and the number of seeds. Ignorant and egocentric nodes have fewer infection occurrences than the other nodes. In contrast, the inactive, cooperative and collaborative nodes keep the infection occurrences close to zero. However, the variability of infection occurrences in evolving networks given a random network, as indicated by the standard deviations, is generally smaller than that in evolving networks given an unconnected network. This indicates that the unconnected backbone network leads to the uncertainty in infection occurrences,

As shown in Fig 3, the infection occurrences in the evolving social networks given a scale-free network show different patterns when the social capital limit changes. The infection occurrences have been kept at zero since the second iteration, given only one seed without an epidemic spread from the seed. This is because the seed in a scale-free backbone network has a higher interaction cost due to its infection and much higher spreading risks considering a greater number of neighbours, which avoids further interactions with the seed and the epidemic spread from the seed. The infection occurrences increase significantly when the number of seeds increases from 1 to 3 and 5. This indicates that more seeds lead to higher infection risks and more difficulty in epidemic control.

**Fig 4:**
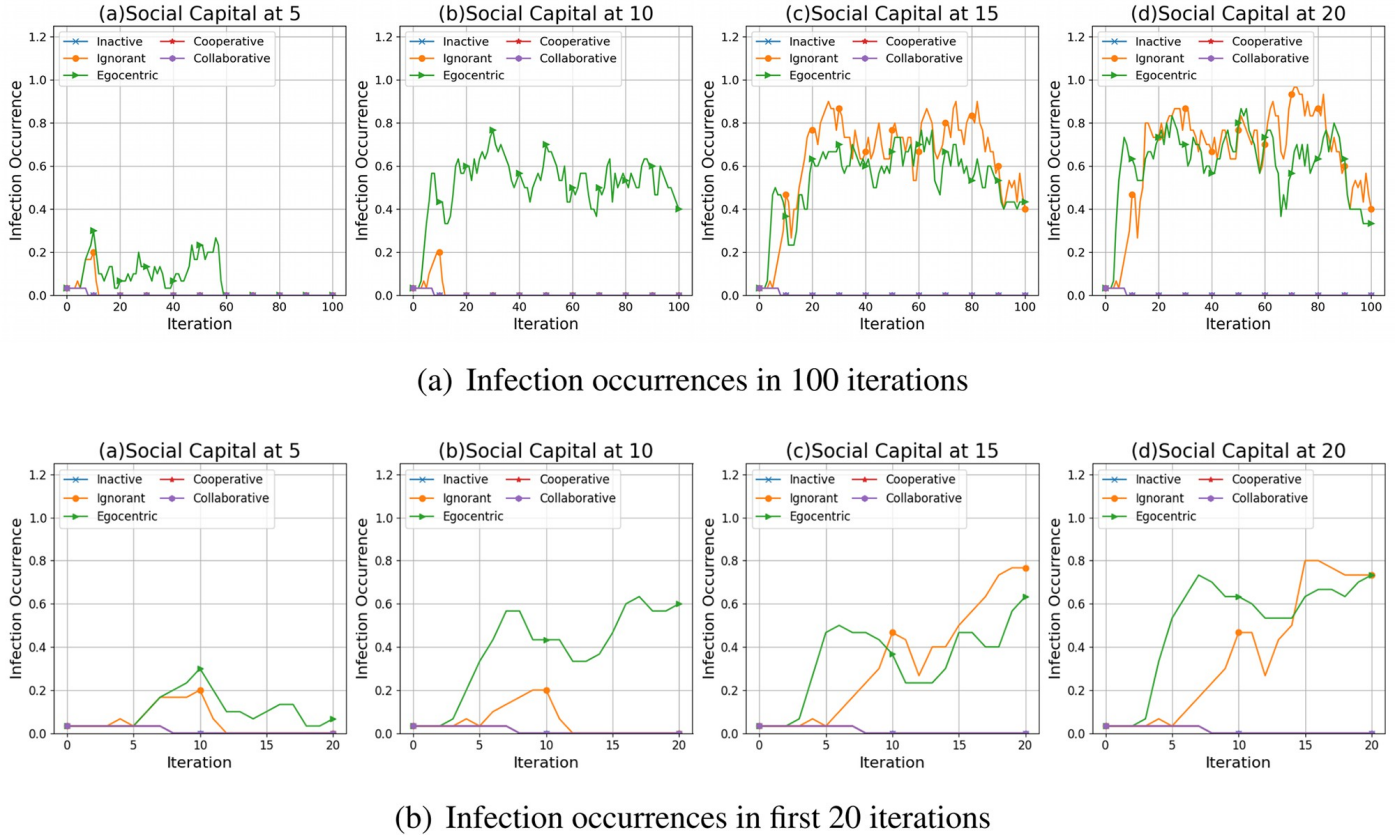
The infection occurrences in 100 iterations (a) and first 20 iterations (b) in the evolving social networks based on an unconnected backbone network under the impact of inactive, ignorant, egocentric, cooperative and collaborative mutation styles.

We take one of the evolving network simulations as an example to illustrate the impact of social capital limit and preference mutation styles on infection patterns. Fig 4 presents the infection occurrence on the evolving networks that are simulated given an unconnected backbone network and one seed for epidemic spread.

As shown in Fig 4, with the increased social capital limit, more infections occur in the epidemic spreading process, where some nodes get infected and some recover. Compared with the other node types, the ignorant and the egocentric nodes are more likely to be infected. Their infection occurrences, given the social capital limit at 15 and 20, fluctuate around 0.70 from the twentieth iteration. This indicates that around 70% of the nodes tend to be infected as the epidemic propagates for more than 20 iterations.This can be caused by the ignorance of group utilities and the unawareness of others’ decisions in individual decision-making processes. In contrast, the inactive, cooperative and collaborative nodes have insignificant infection occurrences. These nodes keep healthy since the tenth iteration (See Fig 4(b)). To better understand the interrelations between the abovementioned infection patterns and the nodes’ interactions, we present and compare the nodes’ interaction patterns driven by different preference mutation styles in Interaction Patterns..

### Interaction patterns

This section investigates the interaction patterns in an epidemic outbreak simulated over 100 iterations. Interactions in the generated networks are driven by different preference mutation styles, including inactive, ignorant, egocentric, cooperative, and collaborative styles. We firstly discuss the variability of interaction numbers given different backbone networks and numbers of seeds (Interaction variability). In addition, we present the evolving networks given an unconnected backbone network and one seed as an example to illustrate the impact of social capital limits and preference mutation styles. We present the visualisations of the evolving social networks in first 20 iterations in Supporting Information and take the most typical ones as examples in the discussion of interaction structures (Interactions). In addition, we compare the social networks driven by different mutation styles by analysing the nodes’ degree values (Degree Distribution) and clustering coefficients (Clustering Coefficient Distribution), and node pairs’ shortest path lengths (Shortest Path Length Distribution). To better understand the differences between the interaction structures of the evolving social networks driven by different preference mutation styles, we also calculate the Euclidean differences between these network statistics.

#### Interaction variability

**Fig 5:**
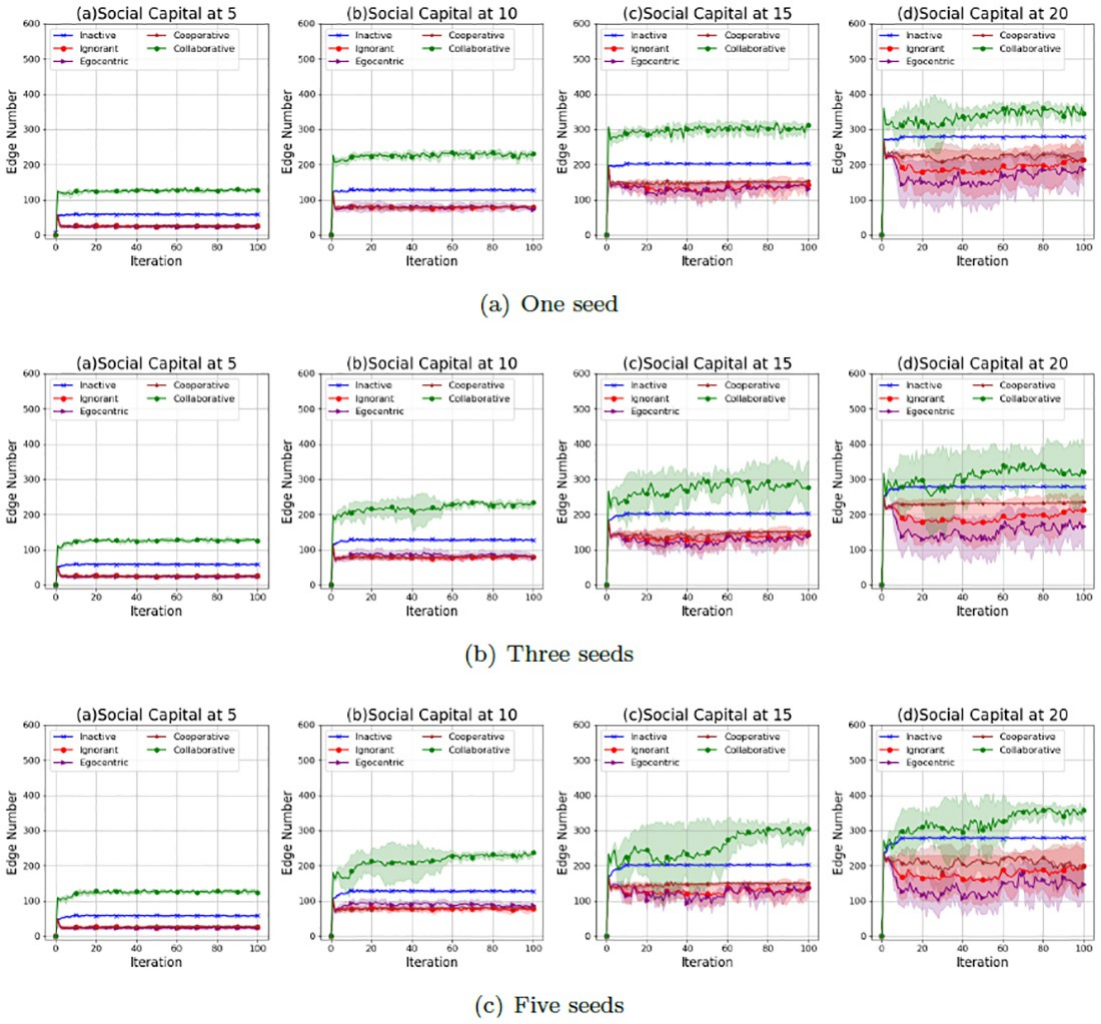
The average values and the standard deviations of interaction numbers in 100 iterations in the evolving social networks based on an unconnected backbone network under the impact of one seed (a), two seeds (b) and three seeds (b). In each scenario, the evolving social networks are respectively driven by inactive, ignorant, egocentric, cooperative and collaborative mutation styles.

**Fig 6:**
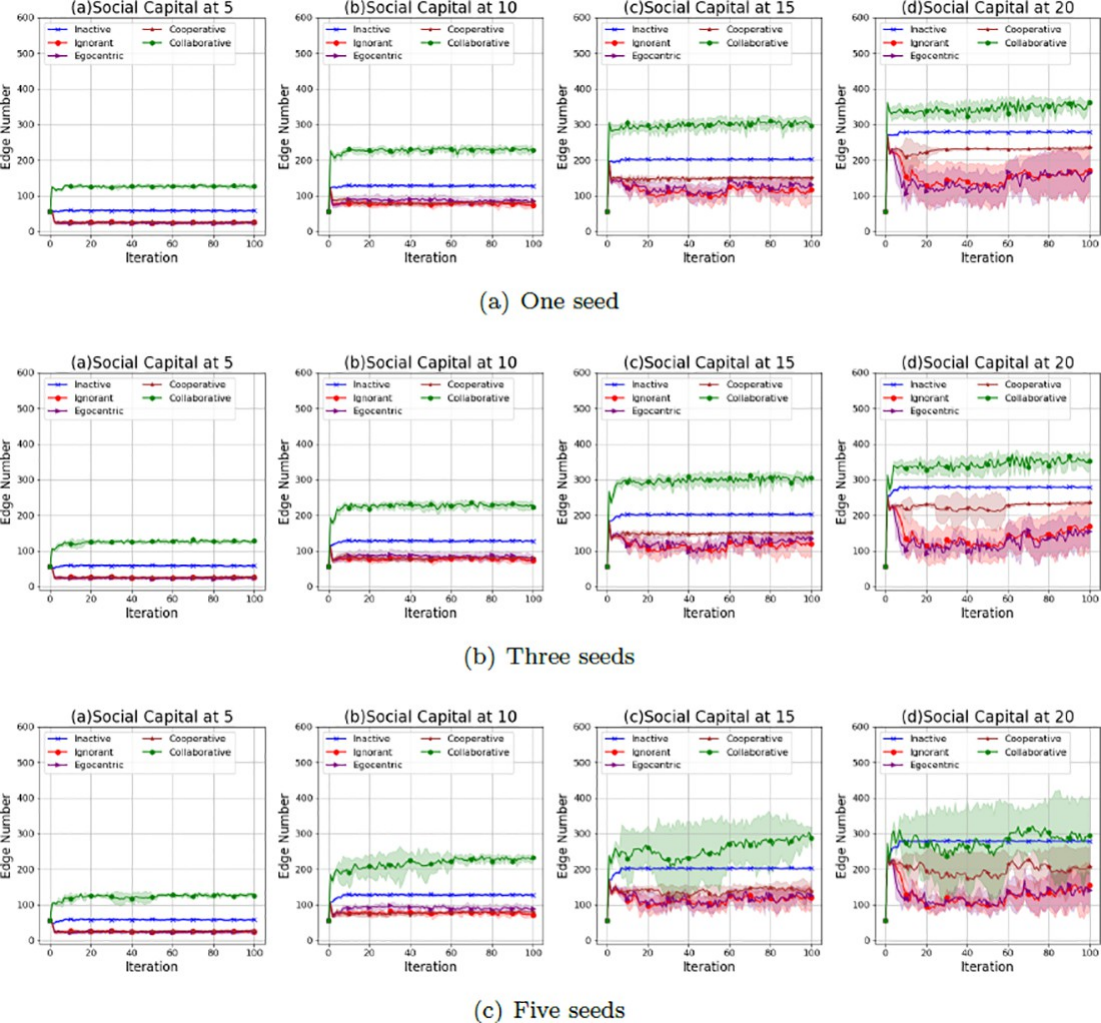
The average values and the standard deviations of interaction numbers in 100 iterations in the evolving social networks based on a random backbone network under the impact of one seed (a), two seeds (b) and three seeds (b). In each scenario, the evolving social networks are respectively driven by inactive, ignorant, egocentric, cooperative and collaborative mutation styles.

**Fig 7:**
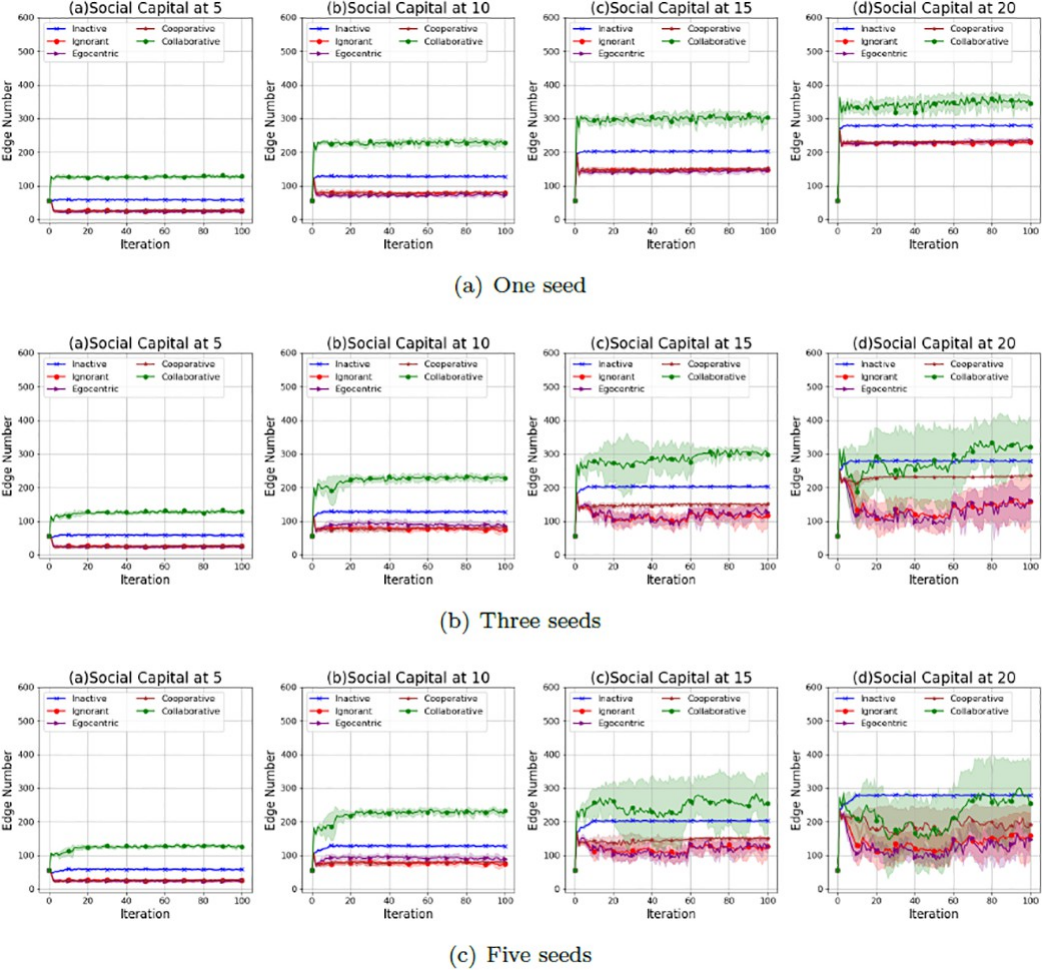
The average values and the standard deviations of interaction numbers in the evolving social networks based on a scale-free backbone network under the impact of one seed (a), two seeds (b) and three seeds (b). In each scenario, the evolving social networks are respectively driven by inactive, ignorant, egocentric, cooperative and collaborative mutation styles.

Figs 5–7 show the average values and the standard deviations of interaction numbers in evolving social networks over 100 iterations given an unconnected backbone network, a random backbone network and a scale-free backbone network.

As shown in Fig 5, the average values and the standard deviations of the interaction number increase with the social capital limit. In addition, the standard deviations increase with the number of seeds. This indicates that a higher social capital limit allows for more flexible interactions and leads to the variability of the interaction number. An increased number of seeds causes more infections (See Fig 1, which leads to fluctuations in interaction numbers over iterations with larger standard deviations. Overall, the collaborative nodes have the highest number of interactions over iterations. This is because collaborative nodes can transfer social capital to each other for more interactions and the corresponding interaction rewards. The inactive nodes have a higher number of interactions than the ignorant nodes, egocentric nodes and cooperative nodes. This is because inactive nodes keep zero preferences for features and feature differences without reacting to the epidemic spread and the interaction reward through preference mutation, which leads to a steady interaction number without variations. In contrast, the interaction number of ignorant nodes, egocentric nodes and cooperative nodes fluctuates with larger standard deviations due to their preference mutation given various infection occurrences in the evolving social networks.

As shown in Fig 6, the average values and the standard deviations of the interaction number of the evolving social networks based on a random backbone network share similar patterns with that of the evolving networks based on an unconnected backbone network. For example, the average interaction number increases with the social capital limit. The standard deviation of the interaction number increases with the social capital limit and the number of seeds, which can be caused by the increased infection occurrences (Se Fig 2). In addition, the collaborative nodes have more interactions than the other types of nodes due to the social capital transfer between the nodes.

As shown in Fig 7, the average values and the standard deviations of the interaction number of the evolving social networks based on a scale-free backbone network share similar patterns with that of the evolving networks based on an unconnected backbone network and the evolving networks based on an unconnected backbone network, without significant differences in interaction numbers over iterations. To have a deeper understanding of the interaction patterns under the impact of social capital limit and preference mutation styles, we take one of the evolving network simulations as an example, where social networks, driven by different preference mutation styles, evolve from an unconnected backbone network under different social capital limits.

#### Interactions

**Fig 8:**
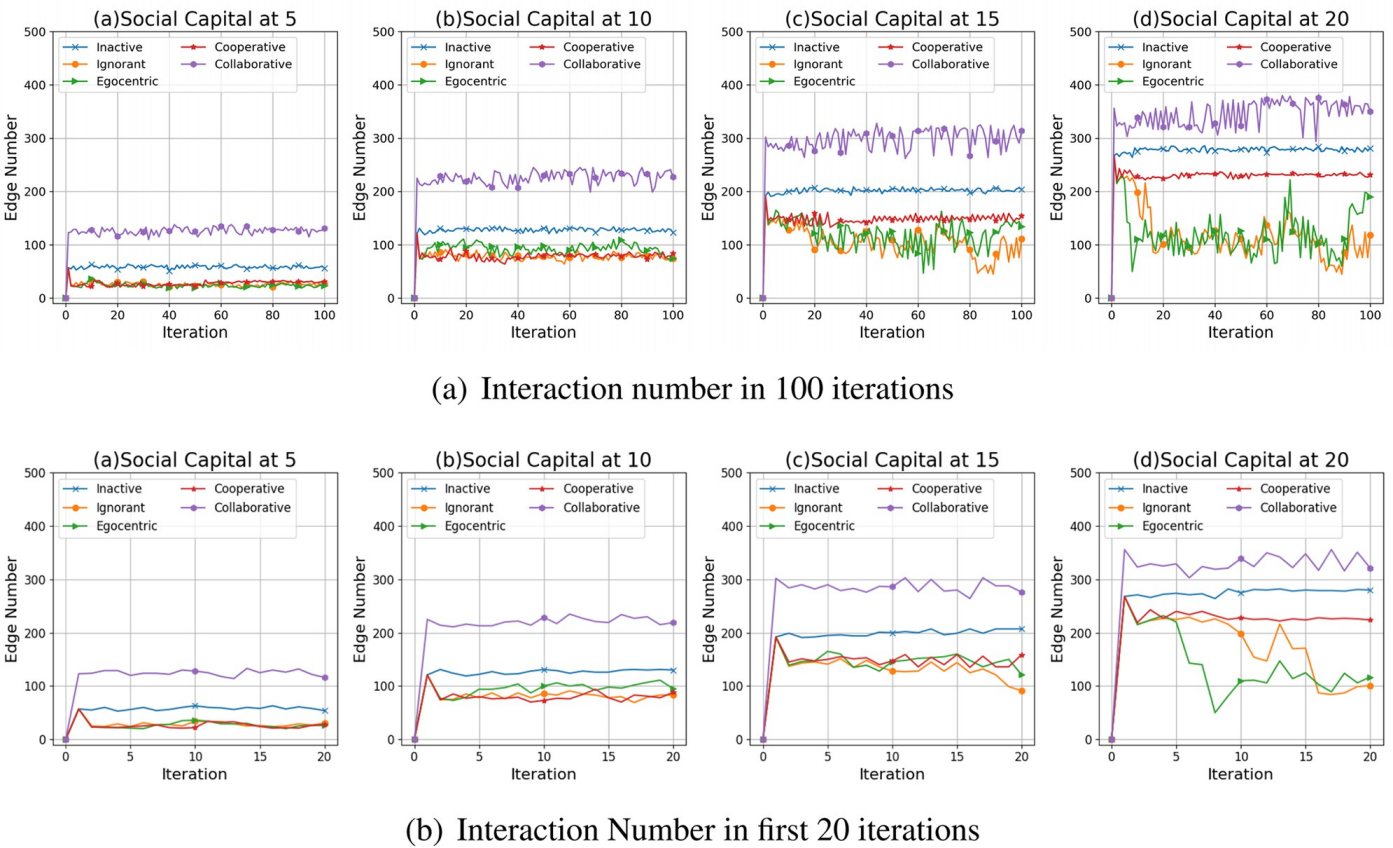
The number of interactions in the 100 simulations steps (a) and first 20 iterations (b) in the evolving social networks based on an unconnected backbone network under the impact of inactive, ignorant, egocentric, cooperative and collaborative mutation styles.

Fig 8(a) and 8(b) show impact on the interactions of different social capital constraints and preference mutation styles in 100 iterations and first 20 iterations. To illustrate the phenomenon presented in Fig 8, we respectively discuss these two aspects based on typical examples of social network simulations presented in Figs 10–12.

When the social capital limit increases, the number of interactions generally increases as nodes can interact more with others (Fig 8). Considering different preference mutation styles, collaborative nodes have the highest interaction numbers over the iterations. This can be caused by the flexible social capital transfer between the collaborative nodes, which enables the creation of more edges with the preferred nodes. In addition, the interaction numbers for each simulated network increase significantly in the first iterations and then fluctuate around a fixed value afterwards, except for the networks driven by the ignorant and the egocentric preference mutation styles under the social capital limit at 15 and 20. This can result from the significant number of infected nodes (See Fig 4(a)) in the ignorant and the egocentric network simulations, which increases the corresponding interaction cost and risk, reducing the interactions with infected nodes. In contrast, due to insignificant infection occurrence and low infection risks, the interaction numbers in networks driven by the inactive, cooperative, and collaborative mutation styles remain steady without significant fluctuations.

**Fig 9:**
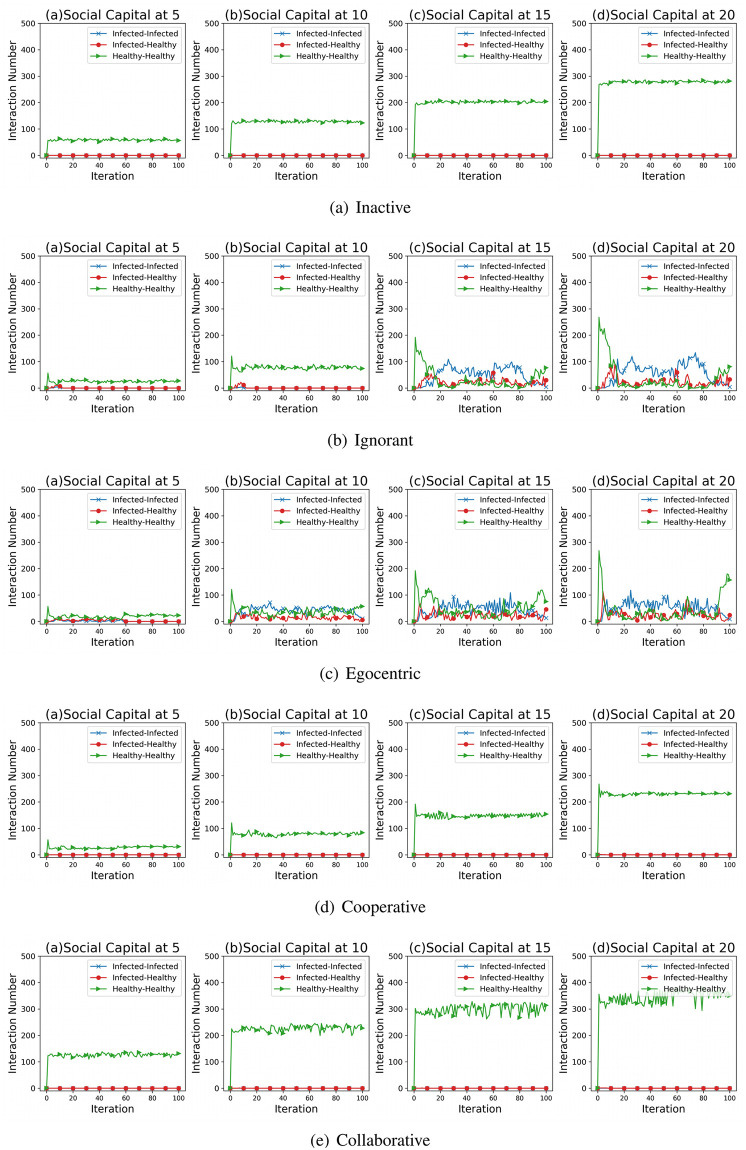
The interaction numbers’ changes between nodes in each health statuses in evolving social networks driven by different mutation styles under different social capital limits ranging within [5, 10, 15, 20]. The mutation styles include the inactive (Fig (a)), the ignorant (Fig (b)), the egocentric (Fig (c)), cooperative (Fig (d)) and the collaborative (Fig (e)) preference mutation styles.

To better understand the interrelations between the interaction numbers and the infection patterns, we present the interaction numbers between nodes with different health statuses in the evolving social networks driven by different mutation styles (Fig 9). In networks driven by the inactive, the cooperative and the collaborative preference mutation styles (See Fig 9(a), 9(d) and 9(e)), social interactions generally take place between the healthy nodes. In contrast, in social network simulations driven by the ignorant and the egocentric preference mutation styles (Fig 9(b) and 9(c)), there are interactions with the infected nodes over the iterations and some of them take place between healthy and infected nodes. These interactions increase significantly as the social capital limit increases from 10 to 20, resulting from and, in turn, reinforcing a high infection occurrence (Fig 4).

**Fig 10:**
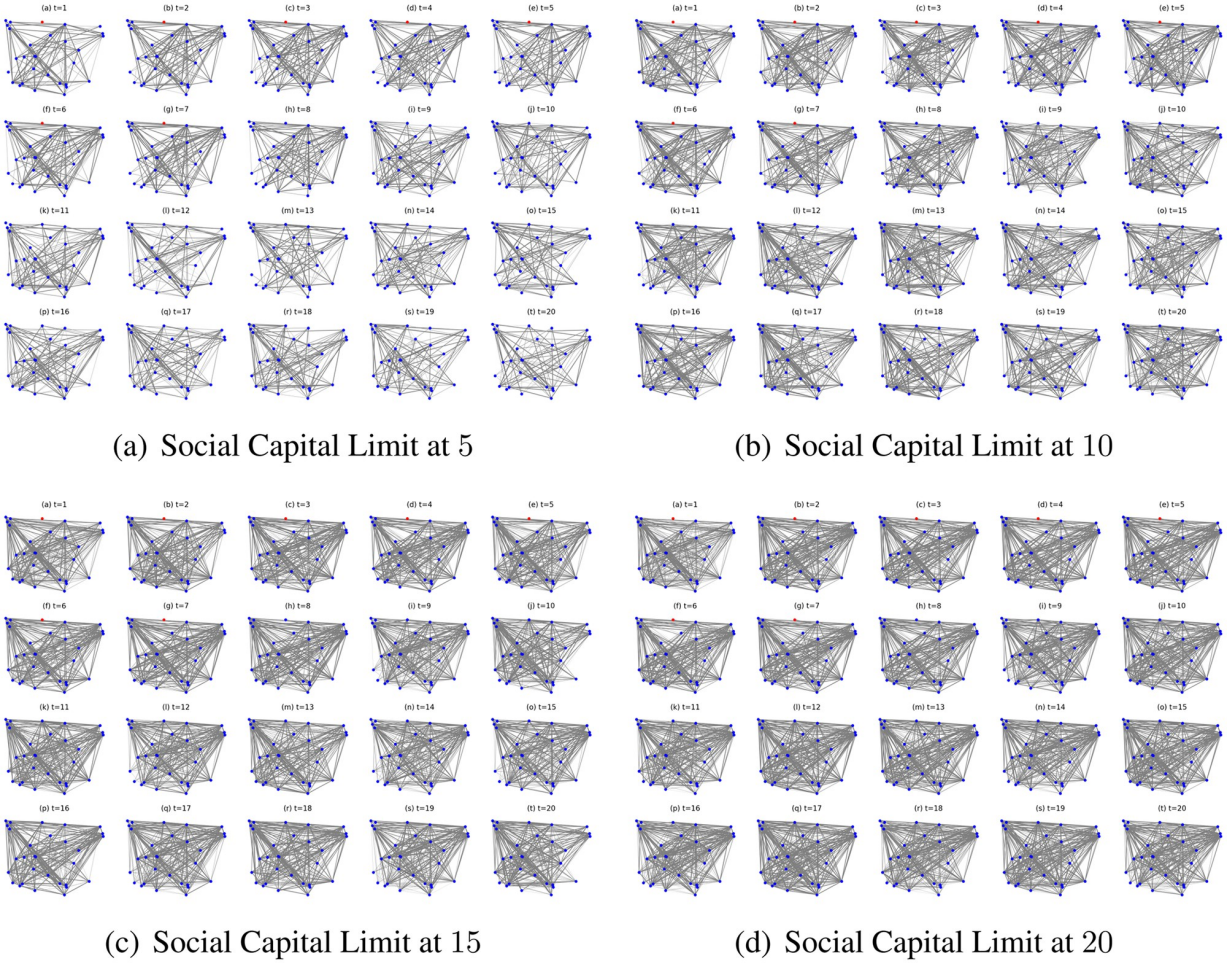
The evolving social networks driven by collaborative preference mutation styles under the impact of different social capital limits.

**Fig 11:**
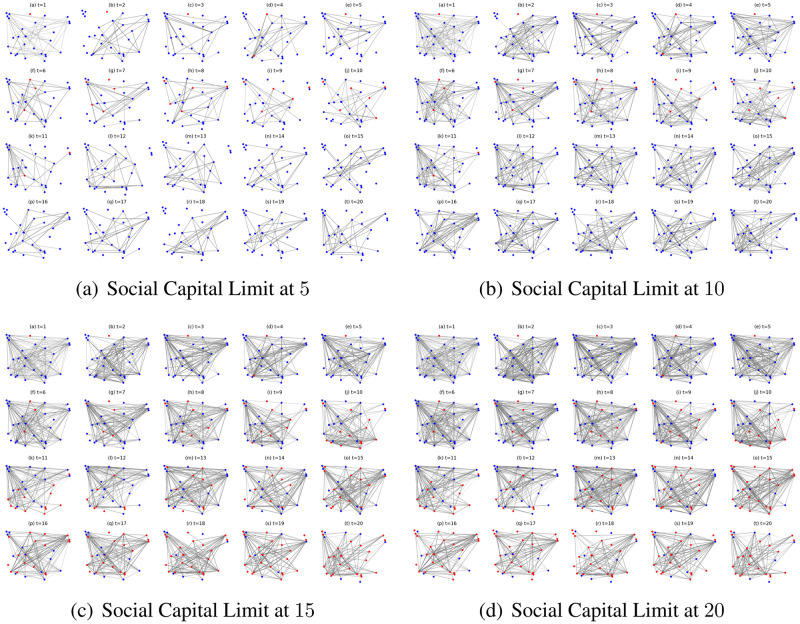
The evolving social networks driven by ignorant preference mutation styles under the impact of different social capital limits.

Since the social networks present different interaction numbers and infection occurrences in the first 20 iterations and afterwards they stay stable with small fluctuations, we present the social network simulations of the ignorant nodes (Fig 11) and the collaborative nodes (Fig 10) in the first 20 iterations under different social capital limits as an example to further explore the network structures and epidemic propagation. As shown in Fig 10, the networks get denser with more interactions given the increased social capital limits. The nodes collaborate to avoid contact with the infected seed node and interact most by reaching their respective social capital limits. In contrast, in Fig 11, the networks in the first ten iterations get denser with more interactions given the increased social capital limits. However, under the social capital limits at 15 and 20, the networks get sparser since the tenth iteration with less interactions and more infections. The decreased interaction number can be caused by the increase in infection occurrence (Fig 11(c) and 11(d)). In addition, as shown in Fig 11(a) and 11(b), under the social capital limit at 5 and 10, there are fewer infections due to a limited number of interactions and exposures to the epidemic spread. This indicates that a lower social capital limit enables to avoid specific interaction risks and limit the impact of epidemic spread on interaction patterns.

**Fig 12:**
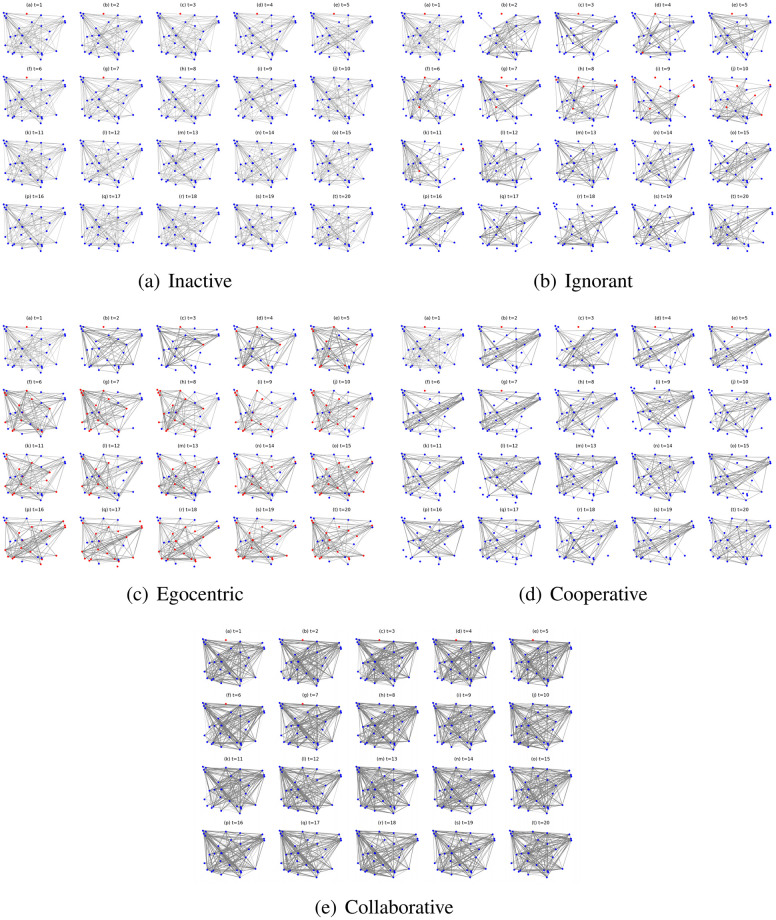
The evolving social networks driven by different mutation styles under the social capital limit at 10. The mutation styles include the inactive (Fig (a)), the ignorant (Fig (b)), the egocentric (Fig (c)), cooperative (Fig (d)) and the collaborative (Fig (e)) preference mutation styles.

We also find from Fig 8 that under the same social capital limit, nodes in the inactive, ignorant and cooperative mutation styles have different number of interactions. To better illustrate this phenomenon, we take the social network simulations under the social capital limit of 10 and driven by different mutation styles as an example (Fig 12). In Fig 12(e), collaborative nodes interact more than other node types. This is because there are few infections, and the collaborative nodes can transfer social capital to enable more interactions. The other types of nodes engage in a similar number of interactions but have different interaction structures and infection patterns. In Fig 12(c), the egocentric nodes have the highest infection occurrence and a significant number of interactions with infected nodes. In contrast, there are fewer infections and limited interactions with the infected nodes in the networks driven by ignorant preference mutation styles. Moreover, there is no social contact with the infected nodes in the networks driven by the inactive and the cooperative preference mutation styles. The abovementioned phenomenon indicates that the egocentric nodes maximise their individual utilities by expanding social contact even though they are infected, detrimental to the utilities of others.

#### Degree distribution

**Fig 13:**
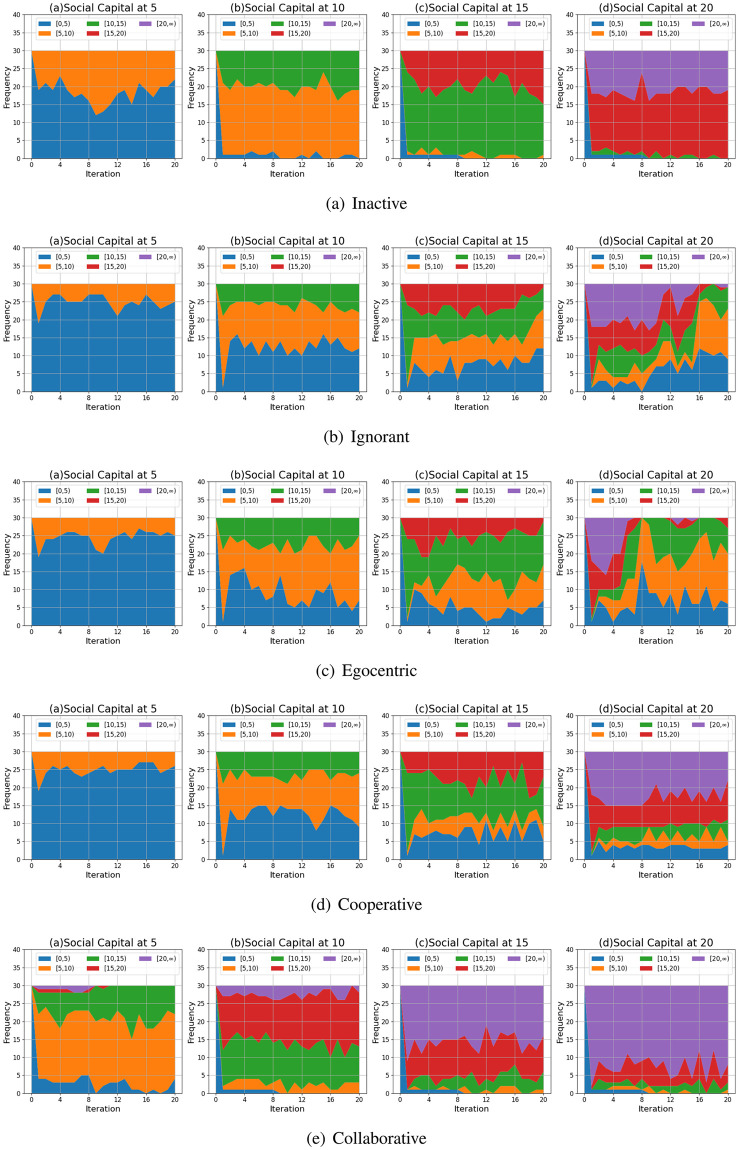
The degree distribution of evolving social networks driven by different mutation styles under different social capital limits ranging within [5, 10, 15, 20]. The mutation styles include the inactive (a), the ignorant (b), the egocentric (c), cooperative (d) and the collaborative (e) preference mutation styles.

**Fig 14:**
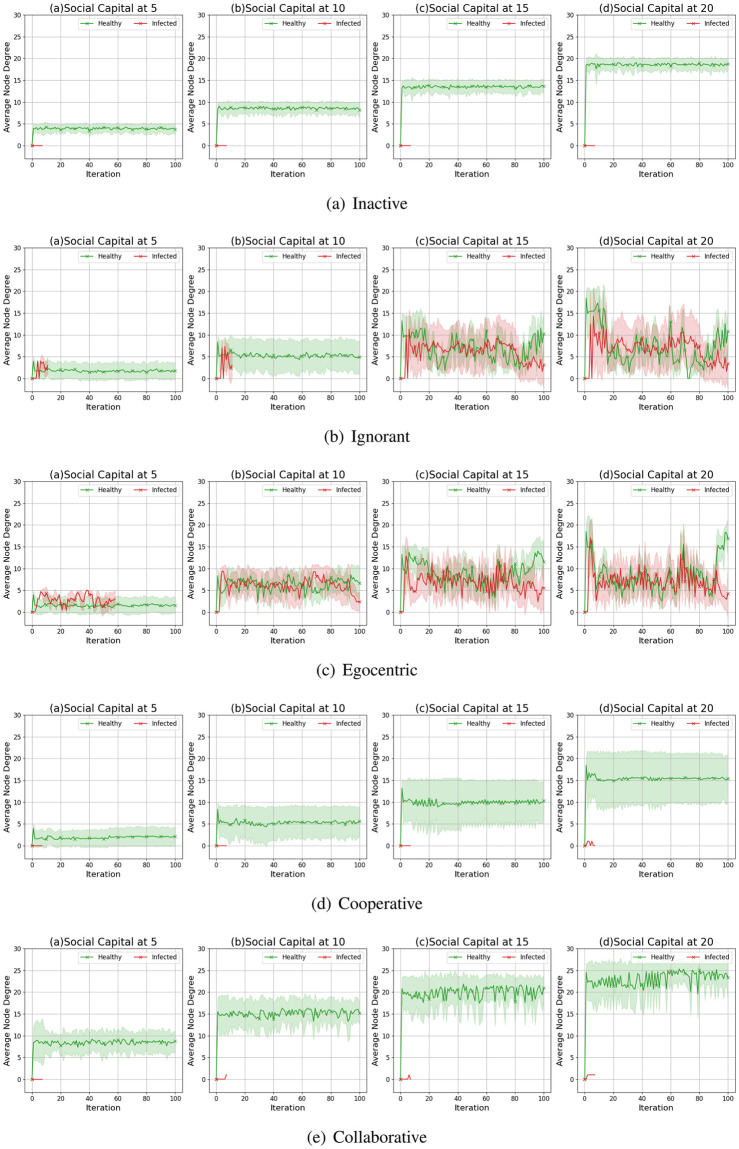
The average node degree for infected nodes and healthy nodes in the evolving social networks driven by different mutation styles under different social capital limits ranging within [5, 10, 15, 20]. The mutation styles include the inactive (Fig (a)), the ignorant (Fig (b)), the egocentric (Fig (c)), cooperative (Fig (d)) and the collaborative (Fig (e)) preference mutation styles.

**Fig 15:**
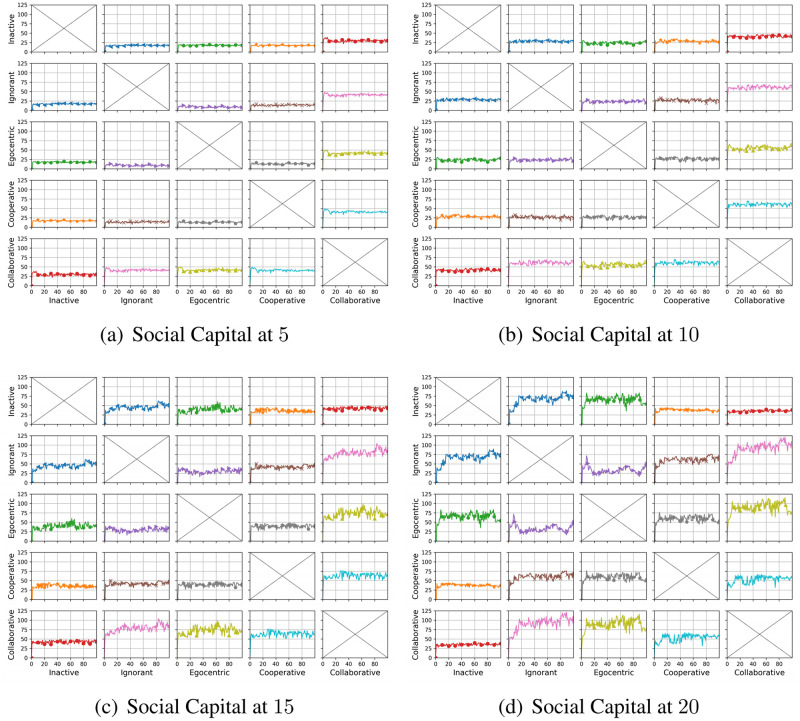
The Euclidean distances between the degree distributions of the evolving interaction networks under different social capital limits, driven by different mutation styles including inactive, ignorant, egocentric, cooperative and collaborative mutation styles. In each subplot, the x-axis represents the number of interactions ranging from 0 to 20, and the y-axis represents the Euclidean distance.

We compare the node degrees of the evolving social networks driven by different preference mutation styles over iterations, including the node degree distributions (Fig 13), average node degrees of the infected nodes and the healthy nodes (Fig 14) and the Euclidean distance values between node degrees in different social networks (Fig 15).

As shown in Fig 13, the node degree values increase with the increasing social capital limit. We take the social networks driven by ignorant nodes as an example (Fig 13(a)). Given a social capital limit of 5, a significant number of ignorant nodes’ degrees are less than 5 and less than 10 nodes’ have a degree value at 5, reaching the social capital limit. The corresponding node degree distribution for ignorant nodes changes as the social capital increases, with more observations of node degrees over 5. In addition, compared with other node types, the collaborative nodes have a generally higher node degree values (Fig 13(e)). The collaborative nodes’ degrees can be higher than the social capital limit as they can transfer social capital flexibly, as nodes with higher social capital can have more social interactions and higher node degrees. The collaborative nodes generally have more interactions and correspondingly, higher node degrees.

To further analyse the impact of infection patterns on the node degrees, we calculate the average values and standard deviations for the infected and healthy nodes (Fig 14). The standard deviations are presented as the width of the shade around average node degrees. In social networks driven by the inactive, cooperative and collaborative preference mutation styles, generally, all the nodes keep healthy over 100 iterations. Their average node degrees keep steady with small fluctuations since the second iteration. In contrast, the average node degrees for healthy ignorant nodes and healthy egocentric nodes increase over the first iteration and afterwards gradually decrease. This can be caused by the decreasing interactions between healthy nodes (Fig 9) and the increasing interaction occurrences in the epidemic outbreak (Fig 4). In the meantime, the average node degrees for the infected ignorant nodes and the infected egocentric nodes fluctuate around the similar average node degree values with that of healthy nodes (Fig 14(b) and 14(c)). We also find that the standard deviations of infected nodes are higher than those of healthy nodes when there are more infected nodes in social networks. Moreover, the standard deviations of node degrees for inactive nodes are significantly smaller than that of others. This is because each inactive node shares a similar connection pattern, where they randomly interact without specific connection preferences, in contrast to other node types.

As shown in Fig 15, we find that the differences between the degree distributions increase significantly when the social capital limit increases (Fig 15). This can be caused by increasing social interactions where nodes can connect with more people and make different choices. Within each subplot, there are more visible differences between the degree distributions of collaborative social networks and other social networks. This is because of the flexible selection of social connections given social capital transfer in group decisions when it comes to the collaborative nodes.

#### Clustering coefficient distribution

**Fig 16:**
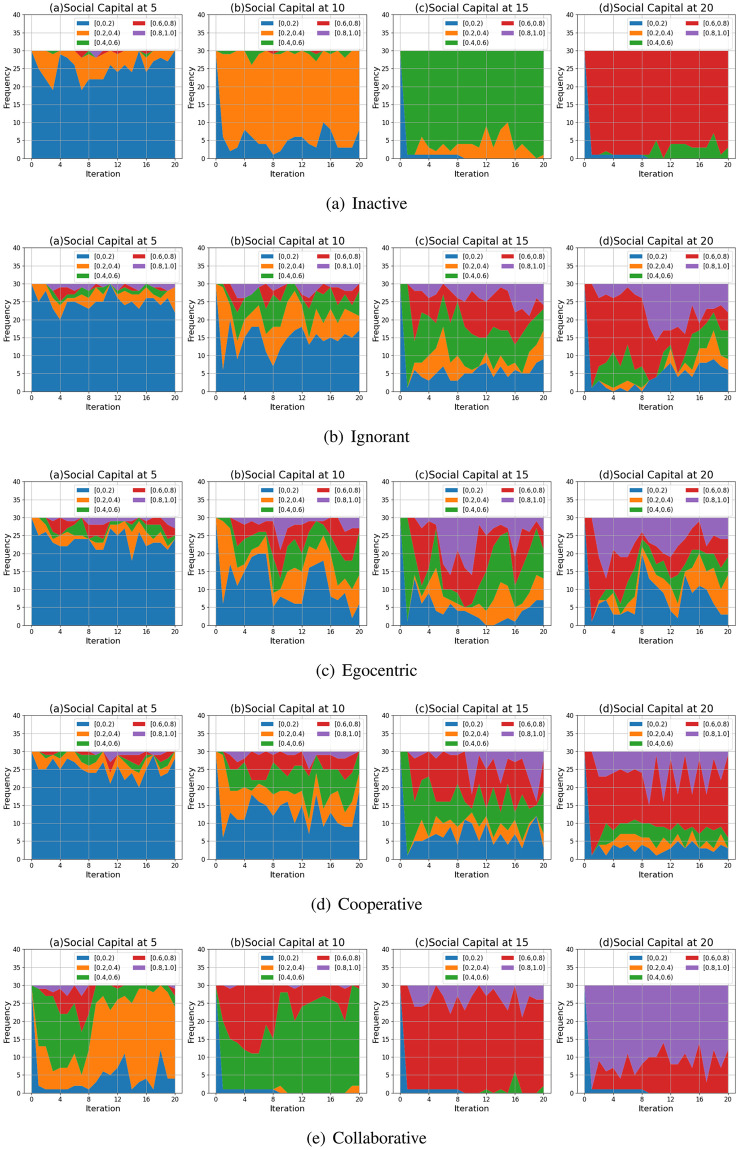
The clustering coefficient distribution of evolving social networks driven by different mutation styles under different social capital limits ranging within [5, 10, 15, 20]. The mutation styles include the inactive (a), the ignorant (b), the egocentric (c), cooperative (d) and the collaborative (e) preference mutation styles.

**Fig 17:**
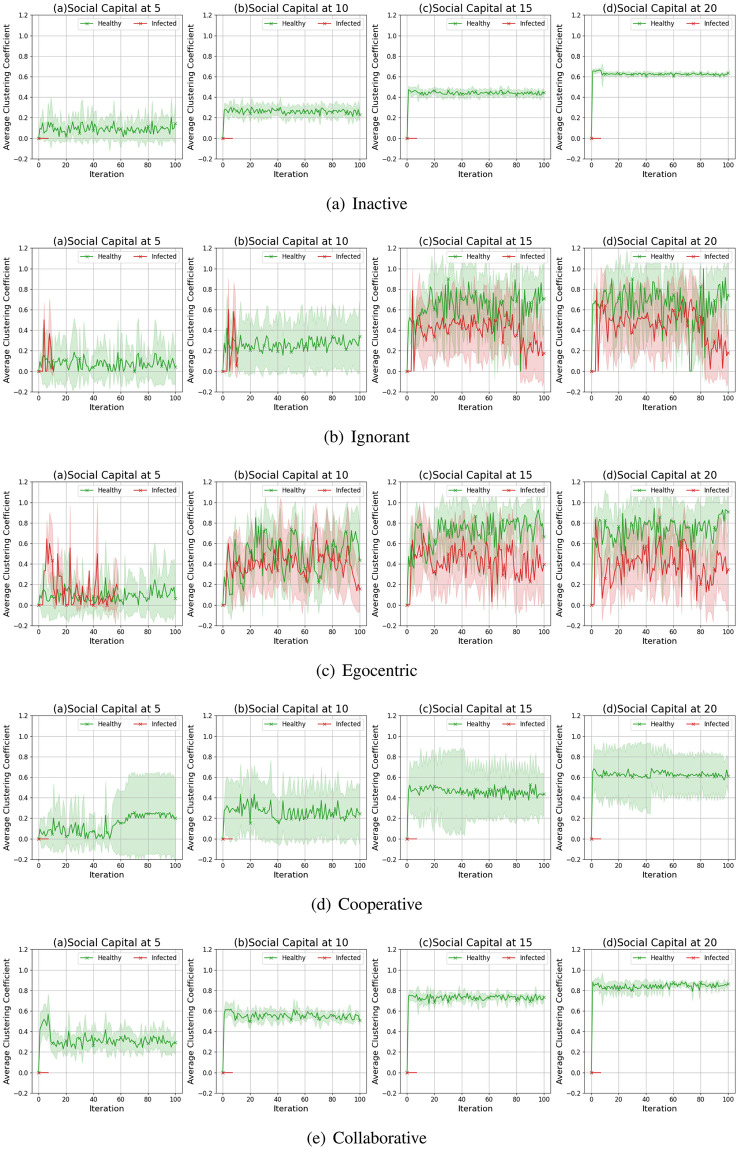
The average node clustering coefficient for infected nodes and healthy nodes in the evolving social networks driven by different mutation styles under different social capital limits ranging within [5, 10, 15, 20]. The mutation styles include the inactive (a), the ignorant (b), the egocentric (c), cooperative (d) and the collaborative (e) preference mutation styles.

**Fig 18:**
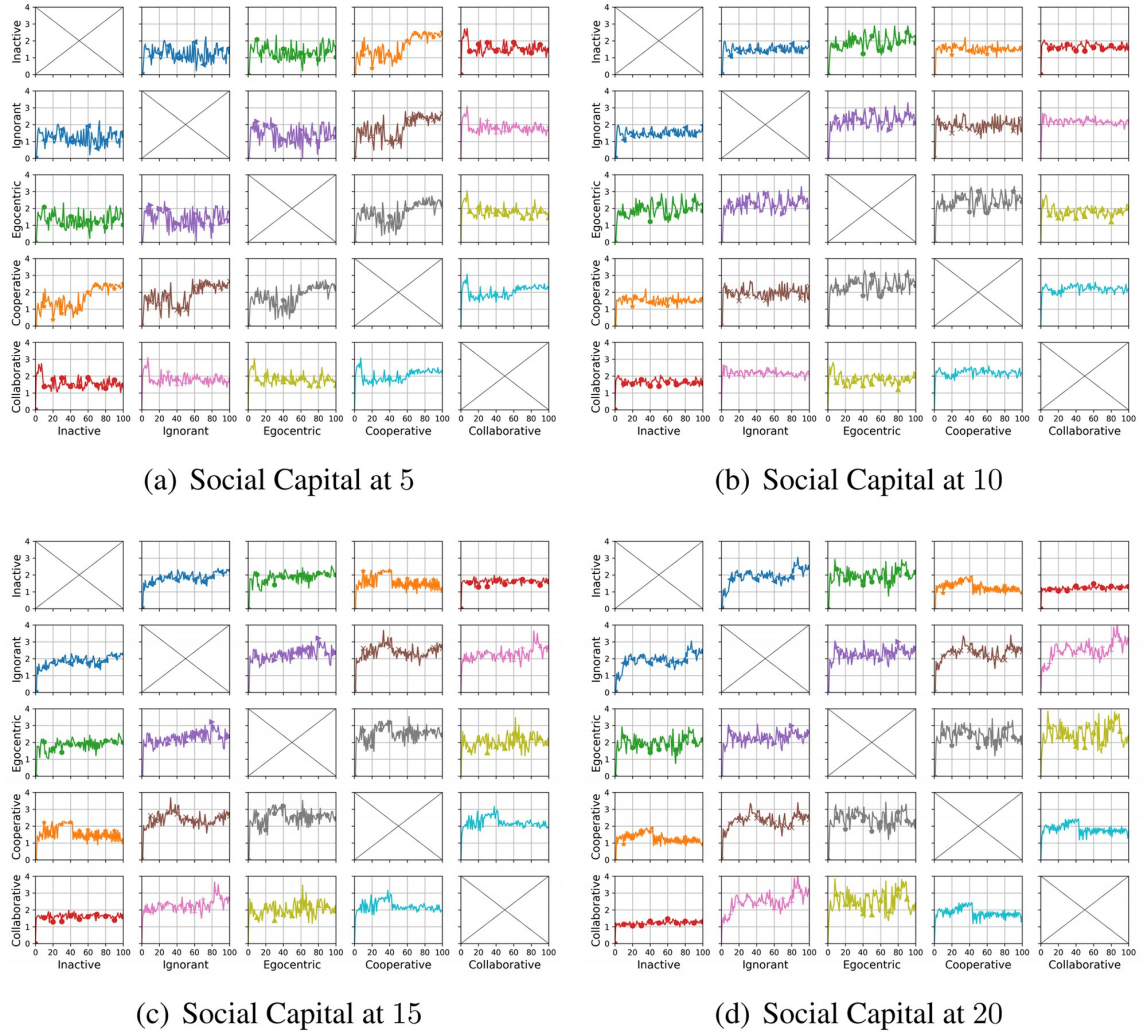
The Euclidean distances between the clustering coefficient distributions of the evolving interaction networks under different social capital limits, driven by different mutation styles including inactive, ignorant, egocentric, cooperative and collaborative mutation styles. In each subplot, the x-axis represents the number of interactions ranging from 0 to 20, and the y-axis represents the Euclidean distance.

We compare the node clustering coefficients of the evolving social networks driven by different preference mutation styles over iterations, including the node clustering coefficient distributions (Fig 16), average node clustering coefficients of the infected nodes and the healthy nodes (Fig 17) and the Euclidean distance values between the node clustering coefficients in different social networks (Fig 18).

As shown in Fig 16, the node clustering coefficient increases as the social capital limit increases. We take the social networks driven by cooperative nodes as an example (Fig 16(d)). Given a social capital limit of 5, more than 20 cooperative nodes’ clustering coefficients are less than 0.20. As the social capital limit increases, more node clustering coefficients take a value larger than 0.20, and some range between [0.80, 1.00]. In contrast, the inactive nodes’ clustering coefficient keep valuing lower than 0.8. Compared with other node types, the collaborative nodes have a generally higher node clustering coefficient values (Fig 13(e)). The abovementioned phenomenon implies that the social capital limits significantly impact the node clustering coefficient. Compared with other node types, collaborative nodes are more likely to cluster around the preferred nodes as they can transfer social capital to others.

In Fig 17, we analyse the impact of infection patterns on the node clustering coefficients by presenting their average values and standard deviations for the infected and healthy nodes. In social networks driven by the inactive and collaborative preference mutation styles (Fig 17(a) and 17(e)), generally, all the nodes keep healthy since the sixth iteration, where their node clustering coefficients have a steady average value and a small standard deviation than that of other node types. Compared with the collaborative nodes, the cooperative nodes have smaller average node clustering coefficients but larger standard deviations (Fig 17(d)). This implies the diversity of node clustering coefficients for cooperative nodes, which can also be seen in Fig 16(d). In contrast to the inactive, cooperative and the collaborative nodes, there is a significant number of infections among the ignorant and the egocentric nodes (Fig 4). The average node clustering coefficients for healthy ignorant nodes and healthy egocentric nodes are generally lower than that of their infected peers (Fig 17(b) and 17(c)). This indicates that the ignorant and egocentric nodes are more likely to cluster around the healthy nodes than the infected ones. In turn, it also implies that healthy ignorant and healthy egocentric nodes are more likely to interact with others. This is because the healthy nodes have lower interaction costs than the infected nodes, which influence the interactions of each node type despite their differences in preference styles.

When it comes to the comparison between clustering coefficient distributions (Fig 18), we find that the differences between them are significant in the first interactions and then slightly fluctuate over time. We find no significant changes in differences between Euclidean Distances when the social capital limit increases. This indicates that increasing interactions can not significantly influence the differences in clustering patterns. In addition, we find that the Euclidean Distances between the collaborative nodes and the egocentric/ignorant nodes are significantly higher than that between other evolving social networks. The collaborative nodes generally keep healthy over the iterations and can transfer social capital to others. This leads to node clusters around the nodes with preferred features, different from the cases of ignorant nodes and egocentric nodes.

#### Shortest path length distribution

**Fig 19:**
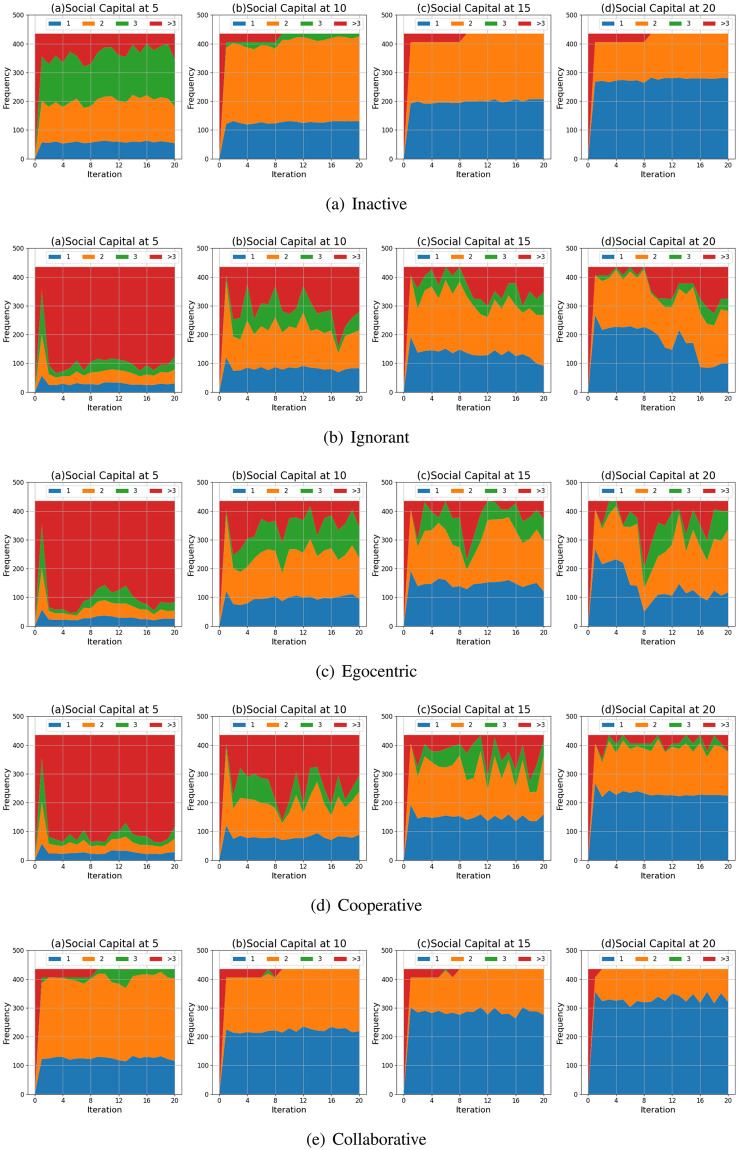
The shortest path length distributions of evolving social networks driven by different mutation styles under different social capital limits ranging within [5, 10, 15, 20]. The mutation styles include the inactive (a), the ignorant (b), the egocentric (c), cooperative (d) and the collaborative (e) preference mutation styles.

**Fig 20:**
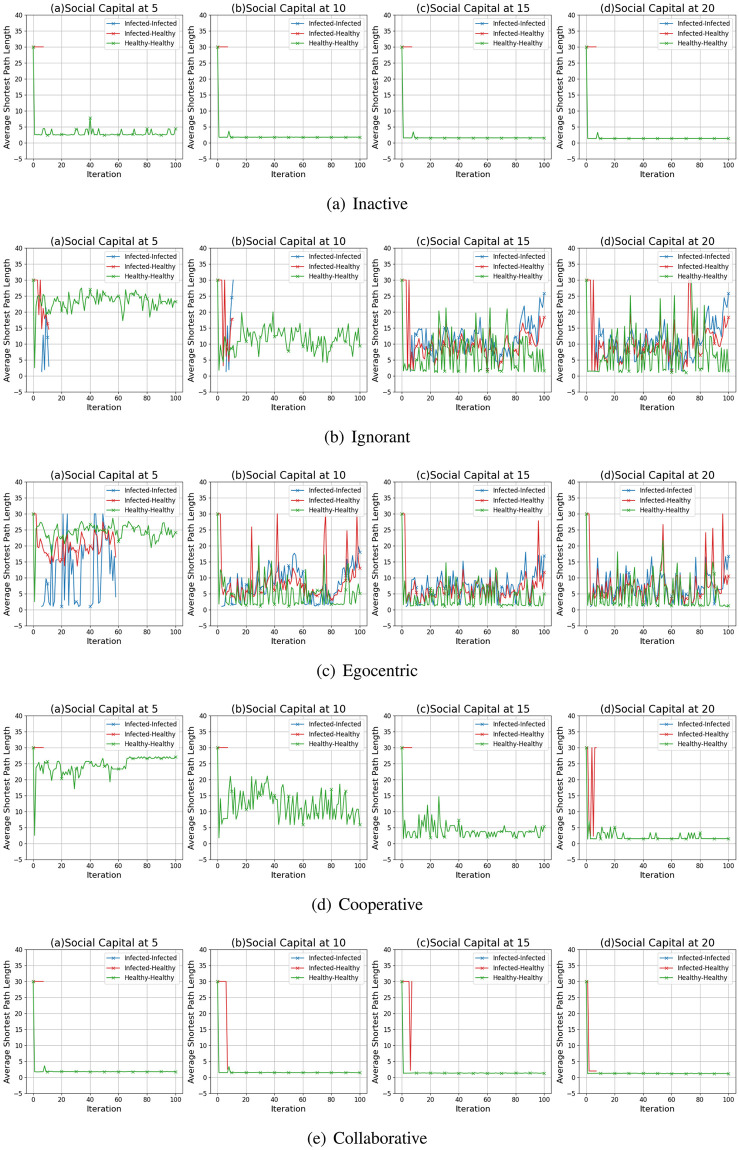
The average shortest path length for infected nodes and healthy nodes in the evolving social networks driven by different mutation styles under different social capital limits ranging within [5, 10, 15, 20]. The mutation styles include the inactive (a), the ignorant (b), the egocentric (c), cooperative (d) and the collaborative (e) preference mutation styles.

**Fig 21:**
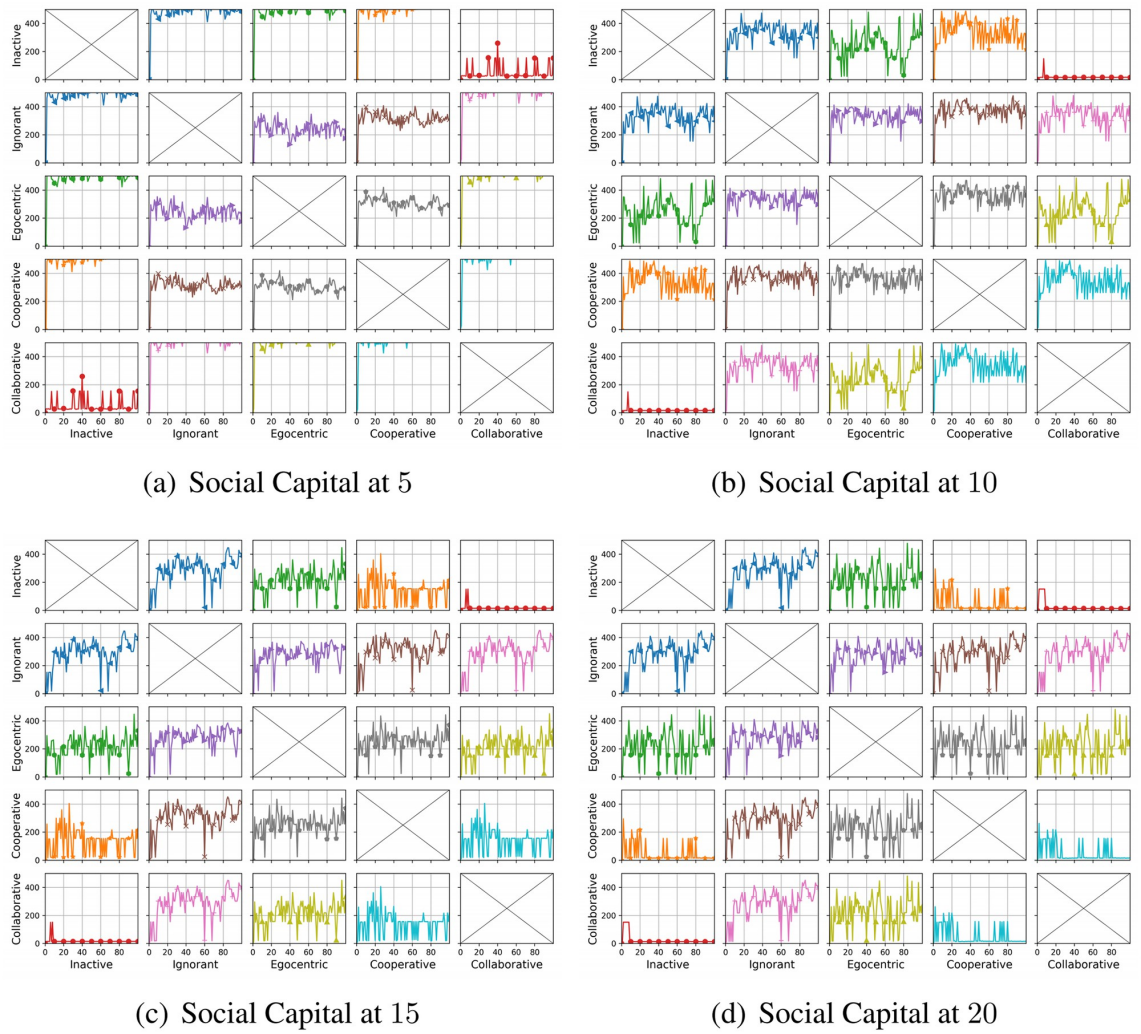
The Euclidean distances between the shortest path length distributions of the evolving interaction networks under different social capital limits, driven by different mutation styles including inactive, ignorant, egocentric, cooperative and collaborative mutation styles. In each subplot, the x-axis represents the number of interactions ranging from 0 to 20, and the y-axis represents the Euclidean distance.

We compare the shortest path lengths of the evolving social networks driven by different preference mutation styles over iterations, including the shortest path length distributions (Fig 19), average shortest path lengths of the node pairs considering their health statuses (Fig 20) and the Euclidean distance values between shortest path lengths in different social networks (Fig 21).

As shown in Fig 19, the shortest path length generally decreases as the social capital limit increases. We take the social networks driven by inactive nodes as an example (Fig 19(a)). Given a social capital limit of 5, the shortest path lengths between half of all inactive node pairs are 3 or over 3. As the social capital limit increases, the average shortest path lengths get shorter, and most inactive node pairs have shortest path lengths below 3. Given a social capital limit at 20, around 280 node pairs are directly connected, with an average shortest path length at 1. In addition, compared with other node types, the collaborative nodes have a generally lower shortest path length (Fig 19(e)). This implies that the social capital limits also significantly impact the shortest path lengths. Moreover, collaborative nodes are more likely to be more tightly connected than the other node types.

In Fig 20, we analyse the impact of infection patterns on the shortest path lengths by presenting their average values for the node pairs considering their health statuses. In social networks driven by the inactive, the cooperative and the collaborative preference mutation styles (Fig 20(a), 20(d) and 20(e)), generally, all the nodes keep healthy since the sixth iteration. Thus, we mainly present the average shortest path lengths between healthy nodes, in contrast to ignorant and egocentric nodes. Among different node types, the average shortest path lengths for inactive nodes and collaborative nodes are lower than 5 given any social capital limits, much smaller than that of other node types. This implies that there are fewer unconnected node pairs, and the inactive/collaborative nodes tend to be tightly connected despite the changes in social capital limit. In contrast, the average shortest path lengths between the cooperative nodes decrease with an increased social capital limit, indicating that more unconnected pairs get connected. Regarding the ignorant and egocentric nodes, the average shortest path lengths between the node pairs in respective health statuses fluctuate significantly. This can result from the fluctuating infection occurrence (Fig 4). In addition, given a social capital limit over 5, the average shortest path lengths between the healthy nodes fluctuate significantly and sometimes reach a lower value of around 1 when compared with the shortest path lengths between the infected and healthy nodes. This implies that the ignorant and the egocentric nodes can avoid the interaction risks by reducing tight connections with the infected nodes. This is because the infected nodes have higher interaction costs, suppressing the corresponding social interactions.

Regarding the comparison between shortest path length distributions (Fig 21), we find that the differences between them are significant when the social capital limit is 5. This implies that decreasing interactions can lead to larger differences in the shortest path length distributions. In addition, we find that the Euclidean Distances between the collaborative nodes and the inactive nodes are significantly lower than that between other evolving social networks. This is because both the inactive nodes and the collaborative nodes are adequately connected in social networks, as presented in the average shortest path lengths (Fig 20(a) and 20(d)).

### Interaction utilities

**Fig 22:**
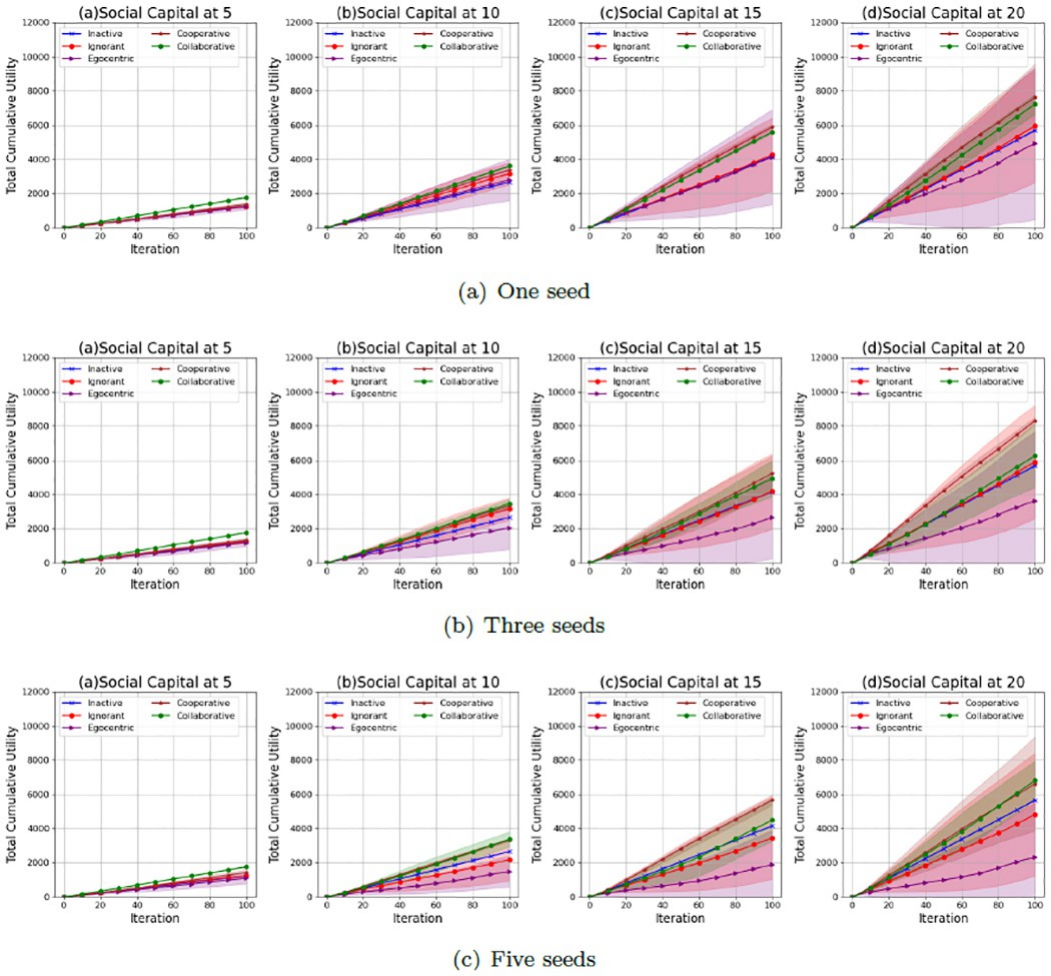
The average values and standard deviations of the cumulative total interaction utilities of nodes in 100 iterations in the evolving social networks based on an unconnected backbone network under the impact of one seed (a), two seeds (b) and three seeds (b). In each scenario, the evolving social networks are respectively driven by inactive, ignorant, egocentric, cooperative and collaborative mutation styles.

**Fig 23:**
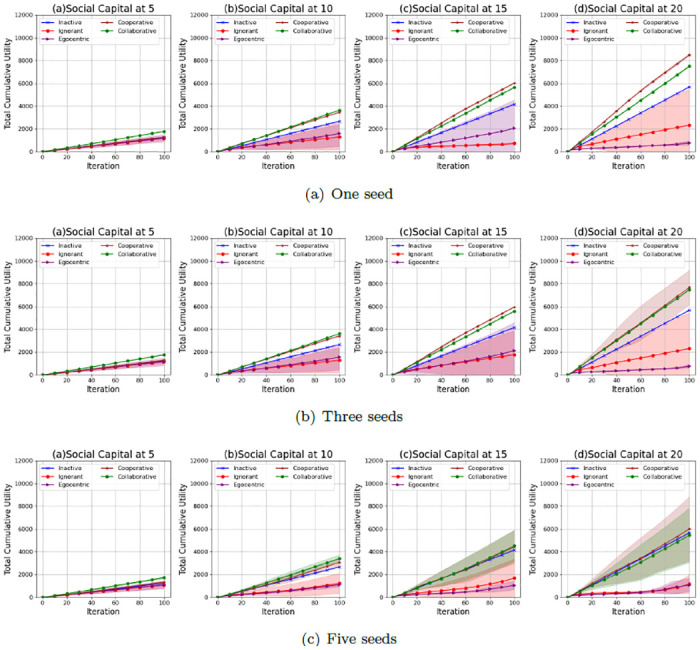
The average values and standard deviations of the cumulative total interaction utilities of nodes in 100 iterations in the evolving social networks based on a random backbone network under the impact of one seed (a), two seeds (b) and three seeds (b). In each scenario, the evolving social networks are respectively driven by inactive, ignorant, egocentric, cooperative and collaborative mutation styles.

**Fig 24:**
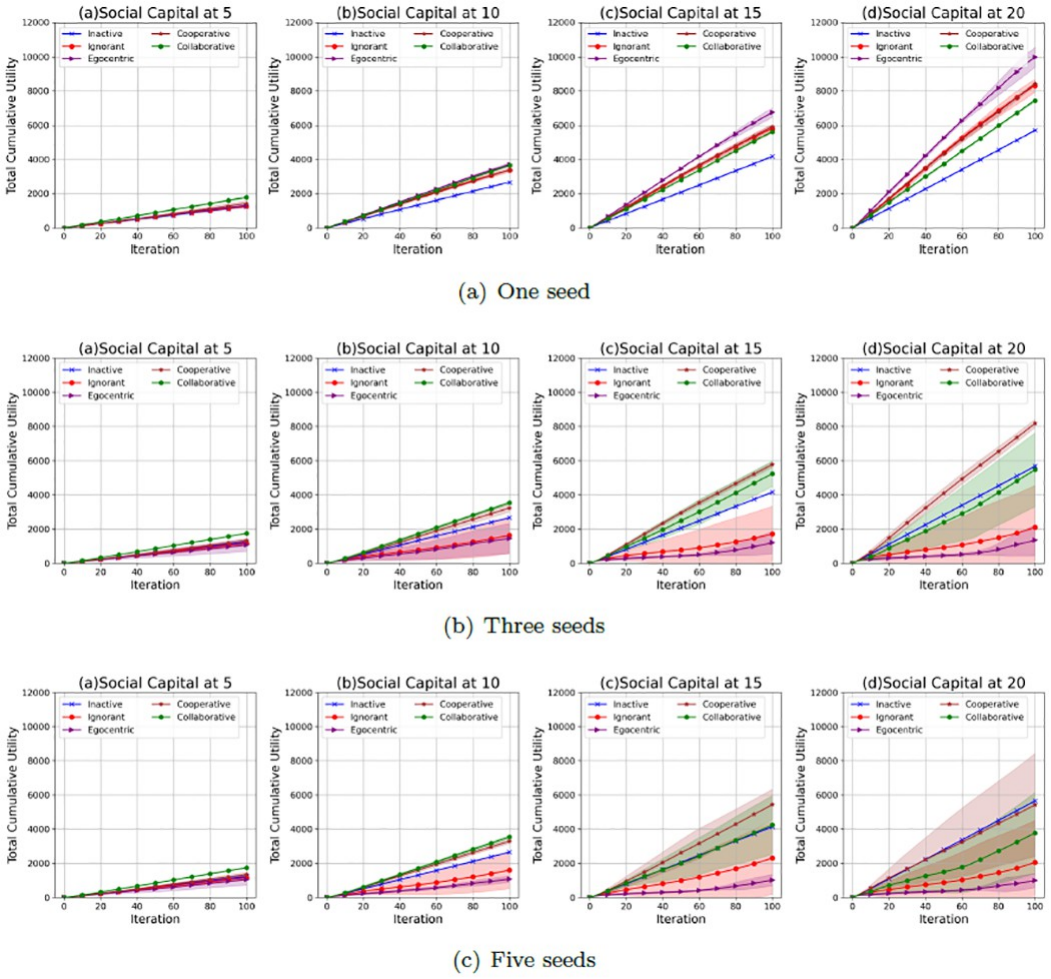
The average values and standard deviations of the cumulative total interaction utilities of nodes in 100 iterations in the evolving social networks based on a scale-free backbone network under the impact of one seed (a), two seeds (b) and three seeds (b). In each scenario, the evolving social networks are respectively driven by inactive, ignorant, egocentric, cooperative and collaborative mutation styles.

We further investigate the interaction utilities of the nodes in the evolving social networks. Figs 22–24 show the average values and the standard deviations of cumulative total interaction utilities for nodes in evolving social networks over 100 iterations given an unconnected backbone network, a random backbone network and a scale-free backbone network.

As shown in Fig 22, the cumulative values of total interaction utilities increase with the social capital limit, which results from the increasing number of interactions and the corresponding interaction rewards. Their standard deviations increase with the social capital limit and the number of seeds. This is because the fluctuations in the infection occurrence (Fig 1) and interaction number (Fig 5) influence the interaction utilities with the interaction risks and the interaction rewards. In addition, the cooperative nodes and the collaborative nodes generally have higher cumulative total interaction utilities than the other types of nodes. This is because cooperative nodes and collaborative nodes can make group decisions based on the group information, while the other types of nodes make individual decisions based on individual information without considering the overall infection risks and interaction rewards. The collaborative nodes can achieve higher average values of cumulative interaction utilities than cooperative nodes given a social capital at 5 and 10. In contrast, the cooperative nodes can achieve higher average values of interaction utilities than collaborative nodes given a social capital at 15 and 20. This indicates that collaborative nodes can improve the overall utilities of all nodes through social capital transfer when the random interference is limited given a smaller social capital limit.

As shown in Fig 23, the cumulative values of total interaction utilities also increase with the social capital limit due to the increasing number of interactions and the corresponding interaction rewards. However, compared with the standard deviations of cumulative total interaction utilities for evolving social networks based on an unconnected backbone network (Fig 22), the standard deviations of cumulative total interaction utilities in evolving social networks based on a random backbone network are smaller, which can be caused by their smaller standard deviations in interactions (Fig 6) and infection occurrences (Fig 2). In addition, the cooperative and collaborative nodes also have higher cumulative interaction utilities than the other types of nodes. The cooperative and collaborative nodes have similar cumulative interaction utilities, whose standard deviations significantly increase when the social capital and the number of seeds increase. This is because the infection occurrence increases, leading to fluctuations in interaction number and the variability of interaction utilities.

As shown in Fig 24, the cumulative values of total interaction utilities also increase with the social capital limit due to the increasing number of interactions and the corresponding interaction rewards. However, compared with the standard deviations of cumulative total interaction utilities for evolving social networks based on an unconnected backbone network (Fig 22) and a random network (Fig 23), the standard deviations of cumulative total interaction utilities in evolving social networks based on a scale-free network given a social capital limit at 5 are much smaller. This is because there is no epidemic spread (Fig 2) and a steady number of interactions (Fig 6). In addition, the cooperative and collaborative nodes generally have higher average values in cumulative interaction utilities and smaller standard deviations than the other types of nodes. However, the cooperative and collaborative nodes do not outperform the inactive nodes when the social capital limit is 20, and the number of seeds is 5, indicating the inefficiency of group decisions.

**Fig 25:**
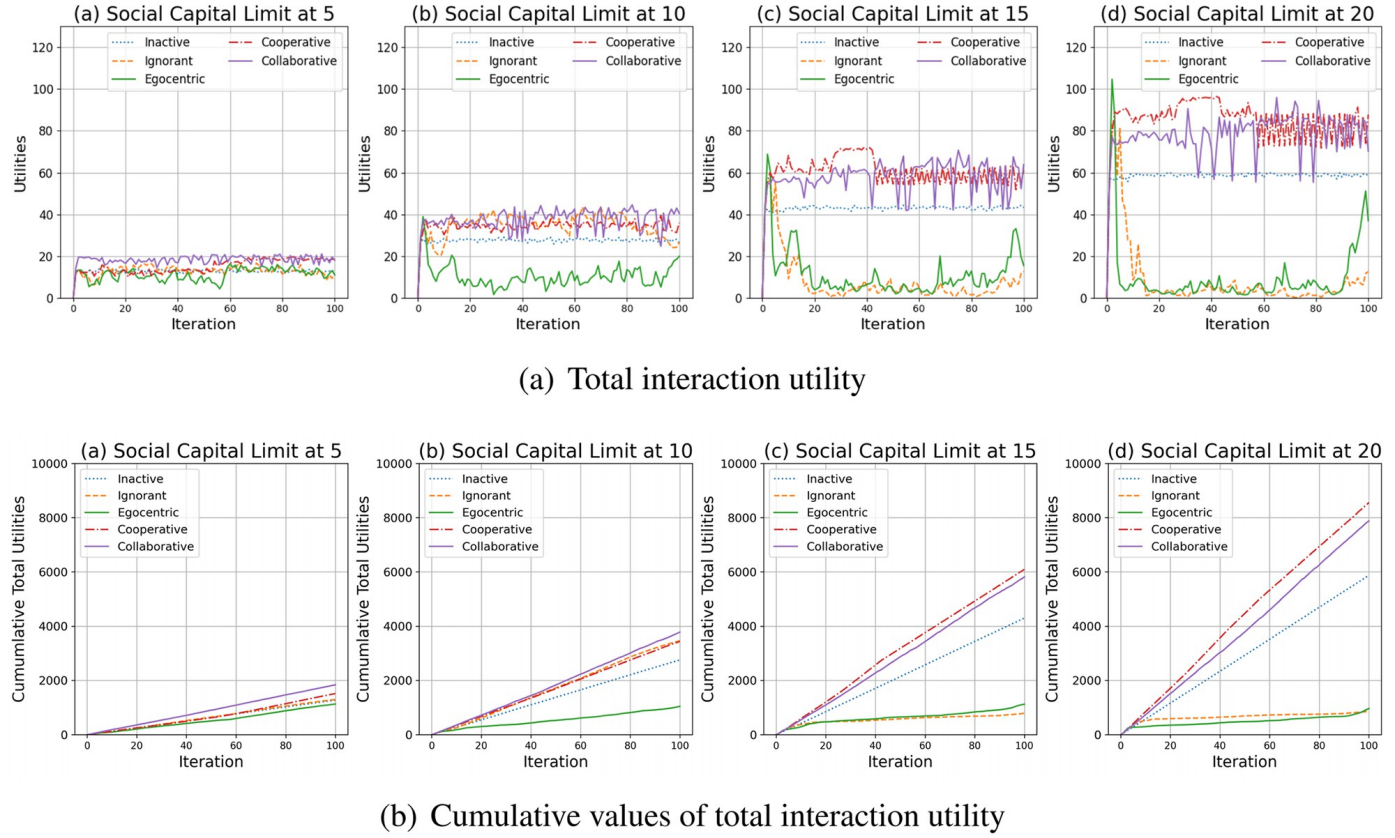
The total interaction utilities of nodes in the evolving social networks (a) and their cumulative values over time (b).

To better understand the abovementioned phenomenon, we discuss the interaction utilities of nodes in the evolving social networks based on an unconnected backbone network. Fig 25 shows the total interaction utilities of nodes in evolving social networks and their cumulative values over time.

As shown in Fig 25(a), the interaction utilities for the inactive, cooperative and collaborative nodes generally increase in the first iterations and keep steady with minor fluctuations. This is because most nodes interact without exposure to the infections. In contrast, the interaction utilities of ignorant and egocentric nodes fluctuate greatly due to many infections (See Fig 4). Compared with the other node types and given any social capital limit, the social interaction utilities of cooperative nodes and collaborative nodes are generally higher.Fig 25(b) shows the cumulative interaction utilities of all nodes in the evolving social networks. We find that the egocentric nodes have the lowest cumulative utilities regardless the social capital limit. This is because the egocentric nodes make inefficient individual decisions without considering the others’ behavioural changes. The collaborative and cooperative nodes can achieve the highest cumulative interaction utilities through group decisions. In addition, as shown in Fig 25(b), the collaborative nodes outperform the cooperative nodes under the social capital limit of 5 and 10 by more interactions and similar infection risks. In contrast, the cooperative nodes outperform the collaborative nodes under the social capital limit of 15 and 20 by fewer interactions and lower interaction risks. This indicates that the interaction risks and the infection occurrences greatly influence the utility maximisation process. A lower social capital limit makes it easier to avoid infection risks under the influence of random interference, ensuring the accuracy of group decisions on social capital transfer and group utility maximisation for collaborative nodes. In contrast, given a social capital limit at 10 or 15, the collaborative nodes have more flexible social capital transfer between the nodes and correspondingly develop more interactions, including the low-intensity ones which are significantly impacted by the random interference. The random interference leads to inaccurate group decision making process and harms the efficiency of interaction utility maximisation.

**Fig 26:**
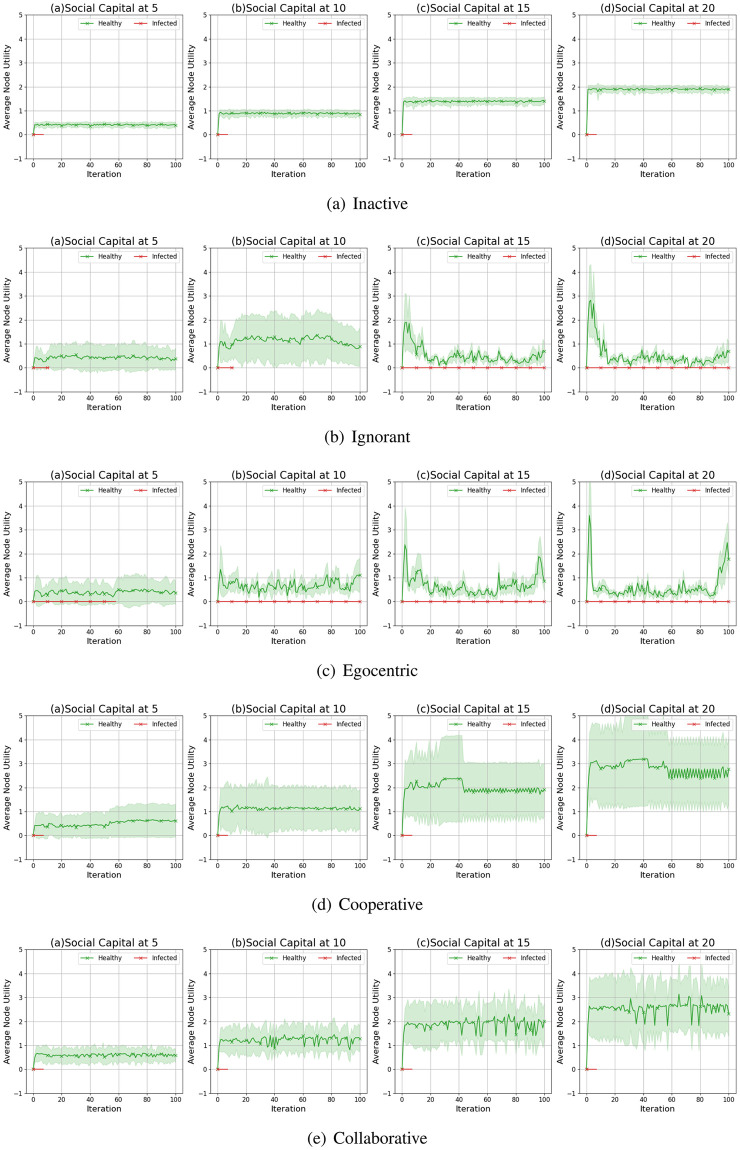
The average utility of infected nodes and healthy nodes in evolving social networks driven by different mutation styles under different social capital limits ranging within [5, 10, 15, 20]. The mutation styles include the inactive (a), the ignorant (b), the egocentric (c), cooperative (d) and the collaborative (e) preference mutation styles.

We present the average values and standard deviations of node utilities for the infected and healthy nodes in Fig 26. The average node utilities of healthy nodes are much higher than those of infected nodes. This is because the infected nodes tend to have higher interaction costs, and their interaction utilities are discounted due to their health statuses. It also indicates that, apart from the social capital limit, the utility discount (0.01 of the aggregated interaction utilities) and the interaction cost (0.60 for infected nodes and 0.40 for healthy nodes) can also suppress the interactions of infected nodes, and thus leading to the infection avoidance effect. Compared with the other node types, the average node utilities for the healthy ignorant nodes and the healthy egocentric nodes are much lower due to the interaction risks given a greater infection occurrence (Fig 4). This also implies that the spread of the epidemic can decrease the utilities of both infected and healthy nodes.

To summarise, first, regarding social capital, increasing social capital enables higher interactions but in the same time results in higher infection risks and uncertainty in decision-making. For example, the inactive, cooperative and collaborative nodes have more interactions when the social capital increases. The ignorant and egocentric nodes make more interactions when there are limited number of infections as the social capital limit increases from 5 to 10. In contrast, the ignorant and egocentric nodes reduce interactions when infection occurrence increases significantly under the social capital limit at 15 and 20. Second, among all the mutation styles, cooperative and collaborative mutation styles achieve higher total utilities than the other mutation styles. This implies that group decisions outperform individual decisions by avoiding the unawareness of the decisions of other nodes. Third, given an insignificant social capital limit that partially excludes the influence of random interference, the collaborative strategy outperforms the inactive, ignorant, egocentric and cooperative strategies by achieving higher interactions, lower infection occurrence and consequently, higher interaction utilities. This indicates that promoting collaborative interactions while imposing strict social capital limits in an epidemic outbreak can effectively reduce infection occurrence and preserve the overall benefit of people.

## Conclusion

This study extends our previous work on the DT-CNS modelling framework related to heterogeneous node features and connection preferences by introducing preference mutation mechanisms [30]. Our previous work enables to model CNSs composed of static networks and a dynamic process on the networks. This study progresses this framework by a substantial extension allowing to model the interrelated component changes in DT-CNSs.

More specifically, we propose an evolutionary DT-CNS modelling framework concerning the nodes’ adaptive decisions on a preference mutation in response to the interaction patterns and epidemic risks. Under this framework, we consider nodes’ heterogeneous features and changeable connection preferences, which combine the effects of preferential attachment, homophily and transitivity. We create heterogeneous preference mutation mechanisms to characterise nodes’ adaptive decisions on preference mutation in response to the interaction patterns and epidemic risks. The preference mutation styles can be inactive, ignorant, egocentric, cooperative and collaborative dependent on the consideration of node utilities and the transferability of social capital between nodes in their decision-making processes.

To build the preference mutation mechanism while incorporating the feedback of interactions and infections, we introduce the social capital limit (which limits the number of possible relationships) to the nodes’ preference mutation process and propose to model nodes’ preference changes as an optimisation process for higher interaction utilities with limited social capital. Nodes can follow heterogeneous preference mutation styles, including the (i)inactive style that keeps zero social preferences, (ii) ignorant style that randomly mutates preferences, (iii) egocentric style that optimises individual interaction utility, (iv) cooperative style that optimises the total interaction utilities by group decisions and (v) collaborative style that further allows the cooperative nodes to transfer social capital.

We conduct extensive simulation-based experiments on the evolutionary DT-CNSs. In the experiments, the nodes interact and mutate their preferences based on heterogeneous strategies, which leads to heterogeneous interaction and infection patterns. We also consider the impact of different number of seeds in epidemic spread and the backbone networks on the evolving social networks. We find that (i) increasing social capital enables more interactions, higher node degrees, higher clustering coefficient, lower shortest path lengths, but more variability in network structures and higher infection risks and uncertainty in decision-making; (ii) group decisions outperform individual decisions by eliminating the unawareness of the decisions of other nodes; (iii) the collaborative strategy, given a strict social capital limit, outperforms the inactive, ignorant, egocentric and cooperative strategies by achieving higher interactions, lower infection numbers and consequently, higher interaction utilities.

In summary, this study proposes an evolutionary DT-CNS framework related to heterogeneous node features and the changeable preferences for connecting with others. This modelling framework can model the interrelated CNS component changes by incorporating the impact of epidemic processes on the network evolution. Our future study will progress this framework by modelling interaction patterns’ influence on the dynamic processes’ transmissibility changes.

## Supporting information

S1 Appendix Social network simulations over twenty iterations based on an unconnected backbone network under social capital limit at 5. (PDF)

S2 Appendix Social network simulations over twenty iterations based on an unconnected backbone network under social capital limit at 20. (PDF)

S3 Appendix Social network simulations over twenty iterations based on an unconnected backbone network under social capital limit at 15. (PDF)

S4 Appendix Social network simulations over twenty iterations based on an unconnected backbone network under social capital limit at 20. (PDF)
