# Supplementary material for: Evolutionary Digital Twin-Oriented Complex Networked Systems driven by node features and the mutation of feature preferences
Source: PLoS One. 2024 May 16;19(5):e0303571. doi: 10.1371/journal.pone.0303571 (PMC11098356; doi:10.1371/journal.pone.0303571)
Supplement: S1 Appendix — (PDF) [file pone.0303571.s001.pdf]

Social network simulations over twenty iterations based on an unconnected backbone network under social capital limit at 5

In this appendix, we present the dynamic social networks generated over twenty iterations considering different social DNA mutation styles, under a social capital limit at 5.

Inactive

Table A. Topological information of the network simulations driven by inactive mutation style under a social capital limit at 5.

Iteration	Nodes		Edges	Node Degree				Clustering coefficient				Shortest path length				
	Connected	Unconnected		Avg.	Std.	Max.	Min.	Avg.	Std.	Max.	Min.	Fake Paths	Avg.	Std.	Max.	Min.
0	30	0	0	0.00	0.00	0	0	0.00	0.00	0	0	435	30.00	0.00	30	30
1	1	29	57	3.8	1.3	5	0	0.09	0.1	0.33	0.00	29	4.34	6.92	30	1
2	1	29	55	3.67	1.27	5	0	0.08	0.1	0.33	0.00	29	4.45	6.89	30	1
3	1	29	60	4.0	1.1	5	0	0.15	0.14	0.5	0.00	29	4.32	6.92	30	1
4	1	29	53	3.53	1.2	5	0	0.05	0.08	0.33	0.00	29	4.44	6.89	30	1
5	1	29	56	3.73	1.24	5	0	0.06	0.09	0.33	0.00	29	4.3	6.92	30	1
6	1	29	60	4.0	1.24	5	0	0.07	0.08	0.2	0.00	29	4.31	6.92	30	1
7	2	28	54	3.6	1.43	5	0	0.15	0.19	0.67	0.00	57	6.14	9.3	30	1
8	1	29	56	3.73	1.46	5	0	0.11	0.13	0.5	0.00	29	4.46	6.9	30	1
9	0	30	60	4.0	1.39	5	1	0.15	0.25	1.00	0.00	0	2.55	0.94	5	1
10	0	30	63	4.2	1.01	5	2	0.13	0.15	0.67	0.00	0	2.47	0.88	5	1
11	0	30	60	4.0	1.21	5	1	0.06	0.09	0.33	0.00	0	2.48	0.88	5	1
12	0	30	59	3.93	1.15	5	1	0.1	0.14	0.67	0.00	0	2.66	1.1	6	1
13	1	29	56	3.73	1.26	5	0	0.07	0.1	0.33	0.00	29	4.34	6.91	30	1
14	0	30	60	4.0	1.21	5	1	0.1	0.11	0.33	0.00	0	2.44	0.85	5	1
15	0	30	58	3.87	1.12	5	1	0.01	0.04	0.17	0.00	0	2.55	0.94	5	1
16	0	30	63	4.2	0.7	5	3	0.11	0.16	0.67	0.00	0	2.43	0.83	4	1
17	0	30	57	3.8	1.28	5	1	0.05	0.09	0.33	0.00	0	2.54	0.92	5	1
18	0	30	61	4.07	0.85	5	2	0.07	0.1	0.33	0.00	0	2.47	0.86	5	1
19	0	30	58	3.87	0.96	5	2	0.07	0.1	0.33	0.00	0	2.47	0.83	4	1
20	0	30	54	3.6	1.08	5	1	0.02	0.05	0.17	0.00	0	2.69	1.00	6	1

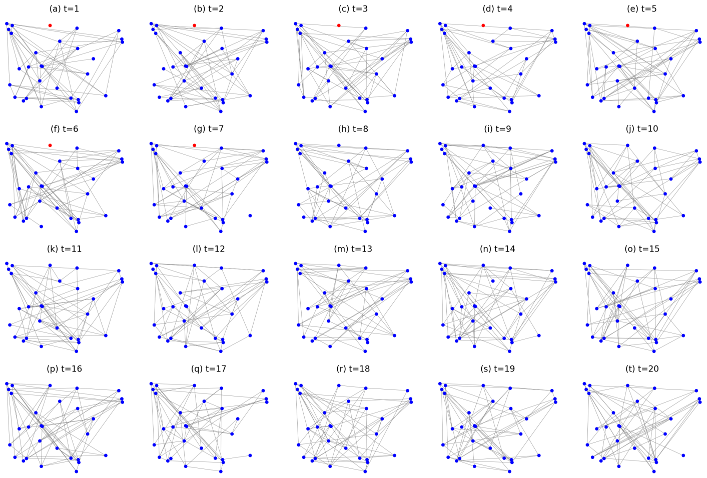

Fig A. The evolving social networks driven by inactive nodes in an epidemic outbreak.

Ignorant

Table B. Topological information of the network simulations driven by ignorant mutation style under a social capital limit at 5.

Iteration	Nodes		Edges	Node Degree				Clustering coefficient				Shortest path length				
	Connected	Unconnected		Avg.	Std.	Max.	Min.	Avg.	Std.	Max.	Min.	Fake Paths	Avg.	Std.	Max.	Min.
0	30	0	0	0.00	0.00	0	0	0.00	0.00	0	0	435	30.00	0.00	30	30
1	1	29	57	3.8	1.3	5	0	0.09	0.1	0.33	0.00	29	4.34	6.92	30	1
2	12	18	25	1.67	1.87	5	0	0.03	0.08	0.3	0.00	314	22.37	12.31	30	1
3	13	17	24	1.6	1.8	5	0	0.14	0.27	1.00	0.00	359	25.14	10.58	30	1
4	10	20	29	1.93	1.75	5	0	0.18	0.28	1.00	0.00	346	24.34	11.18	30	1
5	14	16	24	1.6	1.96	5	0	0.11	0.24	1.00	0.00	343	24.13	11.34	30	1
6	12	18	31	2.07	1.95	5	0	0.11	0.28	1.00	0.00	282	20.4	13.06	30	1
7	11	19	27	1.8	1.85	5	0	0.1	0.22	1.00	0.00	340	23.98	11.41	30	1
8	14	16	28	1.87	1.96	5	0	0.14	0.27	1.00	0.00	315	22.37	12.37	30	1
9	11	19	25	1.67	1.74	5	0	0.06	0.15	0.67	0.00	264	19.35	13.25	30	1
10	8	22	34	2.27	1.82	5	0	0.09	0.22	1.00	0.00	244	18.29	13.29	30	1
11	6	24	33	2.2	1.81	5	0	0.00	0.00	0	0	275	19.97	13.18	30	1
12	11	19	33	2.2	2.12	5	0	0.09	0.26	1.00	0.00	264	19.35	13.26	30	1
13	13	17	29	1.93	2.06	5	0	0.09	0.16	0.67	0.00	299	21.41	12.75	30	1
14	14	16	25	1.67	1.96	5	0	0.1	0.23	1.00	0.00	315	22.4	12.32	30	1
15	16	14	26	1.73	2.06	5	0	0.13	0.26	1.00	0.00	344	24.21	11.26	30	1
16	13	17	24	1.6	1.84	5	0	0.06	0.13	0.5	0.00	329	23.26	11.89	30	1
17	13	17	25	1.67	1.89	5	0	0.07	0.2	1.00	0.00	355	24.86	10.84	30	1
18	15	15	29	1.93	2.14	5	0	0.12	0.24	1.00	0.00	330	23.28	11.92	30	1
19	15	15	27	1.8	2.02	5	0	0.1	0.26	1.00	0.00	330	23.29	11.9	30	1
20	12	18	30	2.0	1.91	5	0	0.11	0.21	1.00	0.00	282	20.34	13.14	30	1

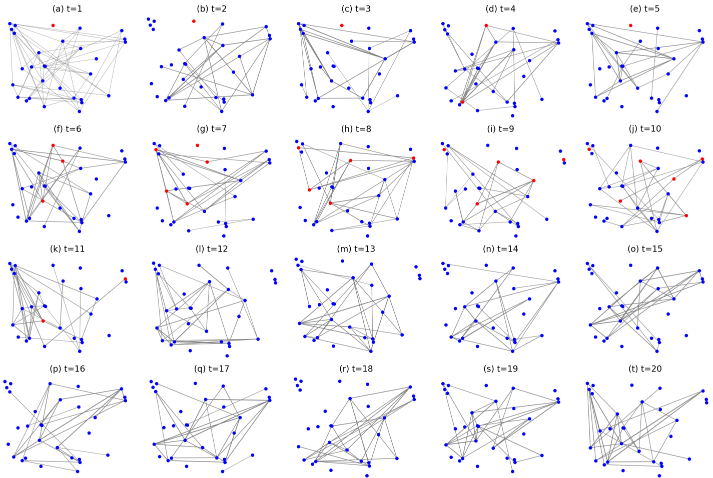

Fig B. The evolving social networks driven by ignorant nodes in an epidemic outbreak.

Egocentric

Table C. Topological information of the network simulations driven by egocentric mutation style under a social capital limit at 5.

Iteration	Nodes		Edges	Node Degree				Clustering coefficient				Shortest path length				
	Connected	Unconnected		Avg.	Std.	Max.	Min.	Avg.	Std.	Max.	Min.	Fake Paths	Avg.	Std.	Max.	Min.
0	30	0	0	0.00	0.00	0	0	0.00	0.00	0	0	435	30.00	0.00	30	30
1	1	29	57	3.8	1.3	5	0	0.09	0.1	0.33	0.00	29	4.34	6.92	30	1
2	16	14	23	1.53	2.03	5	0	0.06	0.14	0.5	0.00	368	25.67	10.15	30	1
3	16	14	22	1.47	2.0	5	0	0.13	0.26	1.00	0.00	368	25.72	10.04	30	1
4	16	14	22	1.47	1.96	5	0	0.15	0.26	0.7	0.00	368	25.71	10.07	30	1
5	16	14	21	1.4	1.84	5	0	0.13	0.25	1.00	0.00	389	27.0	8.71	30	1
6	18	12	20	1.33	1.89	5	0	0.11	0.23	0.83	0.00	369	25.83	9.87	30	1
7	15	15	27	1.8	2.02	5	0	0.1	0.18	0.5	0.00	330	23.33	11.83	30	1
8	13	17	28	1.87	1.94	5	0	0.15	0.32	1.00	0.00	299	21.52	12.6	30	1
9	12	18	35	2.33	2.13	5	0	0.19	0.31	1.00	0.00	282	20.28	13.21	30	1
10	11	19	36	2.4	2.2	5	0	0.19	0.31	1.00	0.00	264	19.17	13.47	30	1
11	12	18	34	2.27	2.05	5	0	0.08	0.2	1.00	0.00	282	20.37	13.1	30	1
12	9	21	29	1.93	1.75	5	0	0.08	0.2	1.00	0.00	263	19.24	13.33	30	1
13	9	21	29	1.93	1.75	5	0	0.06	0.19	1.00	0.00	225	16.94	13.54	30	1
14	12	18	30	2.0	2.0	5	0	0.18	0.25	1.00	0.00	282	20.46	12.98	30	1
15	15	15	24	1.6	1.93	5	0	0.05	0.12	0.5	0.00	330	23.35	11.8	30	1
16	16	14	24	1.6	1.96	5	0	0.14	0.23	1.00	0.00	344	24.2	11.28	30	1
17	16	14	20	1.33	1.8	5	0	0.12	0.22	0.67	0.00	368	25.74	9.99	30	1
18	14	16	25	1.67	1.96	5	0	0.11	0.2	0.6	0.00	315	22.46	12.23	30	1
19	13	17	26	1.73	1.86	5	0	0.21	0.32	1.00	0.00	299	21.64	12.44	30	1
20	13	17	26	1.73	1.9	5	0	0.14	0.28	0.8	0.00	299	21.63	12.45	30	1

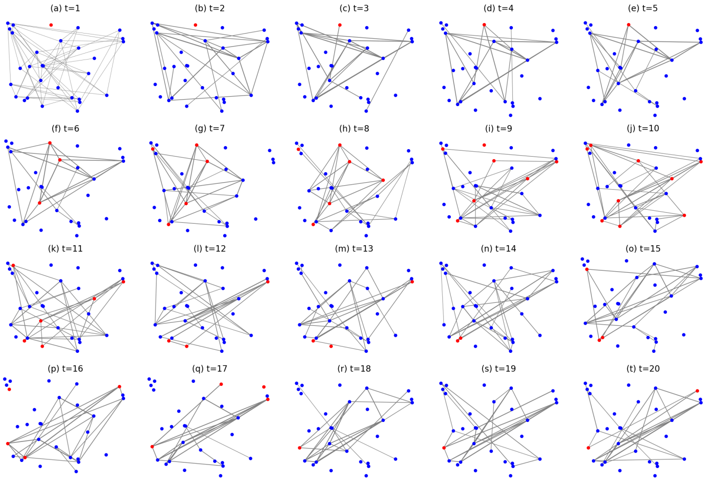

Fig C. The evolving social networks driven by egocentric nodes in an epidemic outbreak.

Cooperative

Table D. Topological information of the network simulations driven by cooperative mutation style under a social capital limit at 5.

Iteration	Nodes		Edges	Node Degree				Clustering coefficient				Shortest path length				
	Connected	Unconnected		Avg.	Std.	Max.	Min.	Avg.	Std.	Max.	Min.	Fake Paths	Avg.	Std.	Max.	Min.
0	30	0	0	0.00	0.00	0	0	0.00	0.00	0	0	435	30.00	0.00	30	30
1	1	29	57	3.8	1.3	5	0	0.09	0.1	0.33	0.00	29	4.34	6.92	30	1
2	14	16	23	1.53	1.94	5	0	0.06	0.15	0.67	0.00	343	24.14	11.33	30	1
3	14	16	23	1.53	1.86	5	0	0.04	0.13	0.67	0.00	354	24.82	10.83	30	1
4	18	12	22	1.47	2.0	5	0	0.07	0.15	0.5	0.00	369	25.74	10.07	30	1
5	15	15	24	1.6	1.91	5	0	0.02	0.07	0.33	0.00	330	23.33	11.84	30	1
6	15	15	25	1.67	2.01	5	0	0.04	0.09	0.33	0.00	356	24.94	10.75	30	1
7	14	16	27	1.8	2.06	5	0	0.08	0.17	0.67	0.00	315	22.37	12.38	30	1
8	16	14	22	1.47	2.0	5	0	0.08	0.17	0.6	0.00	368	25.69	10.1	30	1
9	17	13	21	1.4	1.89	5	0	0.09	0.22	1.00	0.00	357	25.03	10.65	30	1
10	16	14	22	1.47	1.91	5	0	0.05	0.1	0.33	0.00	368	25.69	10.11	30	1
11	10	20	34	2.27	1.86	5	0	0.2	0.34	1.00	0.00	336	23.62	11.77	30	1
12	9	21	32	2.13	1.84	5	0	0.08	0.2	1.00	0.00	323	22.83	12.18	30	1
13	7	23	33	2.2	1.78	5	0	0.12	0.23	1.00	0.00	272	19.71	13.31	30	1
14	10	20	28	1.87	1.82	5	0	0.12	0.25	1.00	0.00	320	22.74	12.12	30	1
15	13	17	24	1.6	1.8	5	0	0.17	0.27	1.00	0.00	329	23.32	11.79	30	1
16	15	15	21	1.4	1.76	5	0	0.06	0.15	0.67	0.00	330	23.38	11.75	30	1
17	16	14	22	1.47	1.82	5	0	0.04	0.18	1.00	0.00	368	25.68	10.12	30	1
18	18	12	21	1.4	1.99	5	0	0.13	0.25	1.00	0.00	369	25.76	10.04	30	1
19	14	16	26	1.73	1.98	5	0	0.1	0.2	0.6	0.00	354	24.8	10.88	30	1
20	12	18	28	1.87	1.91	5	0	0.02	0.06	0.2	0.00	282	20.37	13.09	30	1

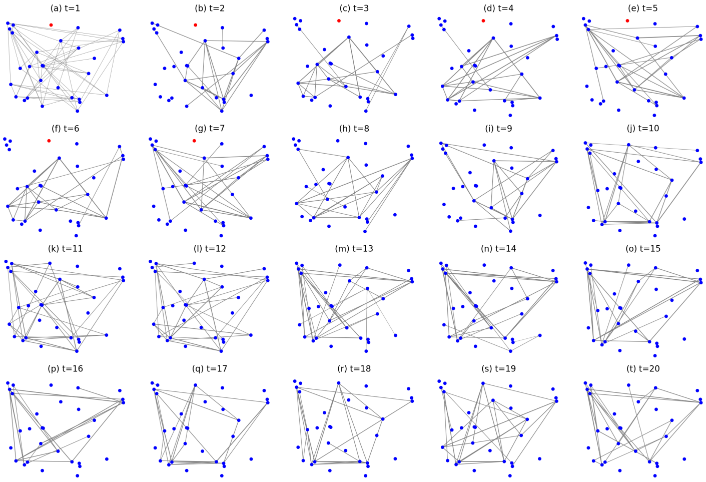

Fig D. The evolving social networks driven by cooperative nodes in an epidemic outbreak.

Collaborative

Table E. Topological information of the network simulations driven by collaborative mutation style under a social capital limit at 5.

Iteration	Nodes		Edges	Node Degree				Clustering coefficient				Shortest path length				
	Connected	Unconnected		Avg.	Std.	Max.	Min.	Avg.	Std.	Max.	Min.	Fake Paths	Avg.	Std.	Max.	Min.
0	30	0	0	0.00	0.00	0	0	0.00	0.00	0	0	435	30.00	0.00	30	30
1	1	29	123	8.2	3.96	21	0	0.41	0.17	1.00	0.00	29	3.63	7.07	30	1
2	1	29	124	8.27	4.57	27	0	0.44	0.16	1.00	0.00	29	3.58	7.07	30	1
3	1	29	129	8.6	4.74	27	0	0.48	0.15	0.8	0.00	29	3.57	7.08	30	1
4	1	29	129	8.6	4.85	27	0	0.5	0.18	1.00	0.00	29	3.57	7.08	30	1
5	1	29	120	8.0	4.6	27	0	0.48	0.19	1.00	0.00	29	3.61	7.07	30	1
6	1	29	124	8.27	4.71	26	0	0.44	0.17	0.7	0.00	29	3.61	7.07	30	1
7	1	29	124	8.27	5.54	26	0	0.55	0.22	1.00	0.00	29	3.63	7.07	30	1
8	1	29	122	8.13	4.65	26	0	0.46	0.16	0.7	0.00	29	3.6	7.07	30	1
9	0	30	130	8.67	2.52	14	5	0.3	0.09	0.5	0.1	0	1.74	0.52	3	1
10	0	30	128	8.53	2.83	16	4	0.28	0.09	0.47	0.1	0	1.74	0.51	3	1
11	0	30	125	8.33	2.87	13	2	0.3	0.13	0.5	0.00	0	1.82	0.6	3	1
12	0	30	118	7.87	2.73	13	3	0.27	0.12	0.6	0.00	0	1.85	0.6	3	1
13	0	30	114	7.6	2.8	12	1	0.25	0.12	0.43	0.00	0	1.9	0.65	4	1
14	0	30	133	8.87	2.51	14	2	0.31	0.09	0.5	0.00	0	1.75	0.55	3	1
15	0	30	125	8.33	1.94	13	4	0.27	0.07	0.43	0.14	0	1.76	0.52	3	1
16	0	30	130	8.67	1.85	12	5	0.29	0.08	0.5	0.14	0	1.74	0.52	3	1
17	0	30	126	8.4	2.2	13	3	0.29	0.07	0.5	0.19	0	1.76	0.53	3	1
18	0	30	132	8.8	1.38	11	6	0.23	0.07	0.39	0.00	0	1.72	0.5	3	1
19	0	30	122	8.13	2.08	13	4	0.28	0.09	0.48	0.1	0	1.79	0.55	3	1
20	0	30	116	7.73	2.69	14	4	0.31	0.15	0.8	0.14	0	1.81	0.55	3	1

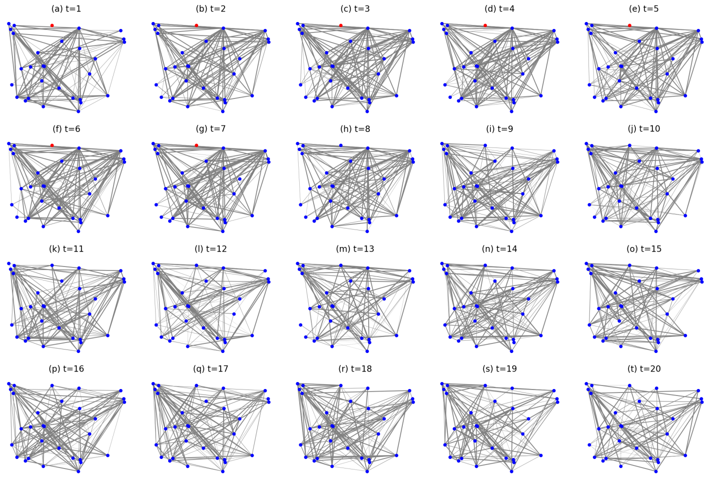

Fig E. The evolving social networks driven by collaborative nodes in an epidemic outbreak.
