# Supplementary material for: Evolutionary Digital Twin-Oriented Complex Networked Systems driven by node features and the mutation of feature preferences
Source: PLoS One. 2024 May 16;19(5):e0303571. doi: 10.1371/journal.pone.0303571 (PMC11098356; doi:10.1371/journal.pone.0303571)
Supplement: S2 Appendix — (PDF) [file pone.0303571.s002.pdf]

Social network simulations over twenty iterations based on an unconnected backbone network under social capital limit at 10

In this appendix, we present the dynamic social networks generated over twenty iterations considering different social DNA mutation styles, under a social capital limit at 10.

Inactive

Table A. Topological information of the network simulations driven by inactive mutation style under a social capital limit at 10.

Iteration	Nodes		Edges	Node Degree				Clustering coefficient				Shortest path length				
	Connected	Unconnected		Avg.	Std.	Max.	Min.	Avg.	Std.	Max.	Min.	Fake Paths	Avg.	Std.	Max.	Min.
0	30	0	0	0.00	0.00	0	0	0.00	0.00	0	0	435	30.00	0.00	30	30
1	1	29	121	8.07	2.1	10	0	0.26	0.08	0.4	0.00	29	3.63	7.07	30	1
2	1	29	131	8.73	1.93	10	0	0.27	0.08	0.46	0.00	29	3.57	7.08	30	1
3	1	29	124	8.27	2.1	10	0	0.26	0.08	0.38	0.00	29	3.6	7.07	30	1
4	1	29	119	7.93	2.13	10	0	0.24	0.08	0.36	0.00	29	3.64	7.06	30	1
5	1	29	122	8.13	2.28	10	0	0.27	0.1	0.42	0.00	29	3.64	7.06	30	1
6	1	29	127	8.47	1.93	10	0	0.29	0.08	0.42	0.00	29	3.6	7.07	30	1
7	1	29	122	8.13	2.11	10	0	0.24	0.06	0.33	0.00	29	3.62	7.07	30	1
8	1	29	123	8.2	2.18	10	0	0.29	0.1	0.67	0.00	29	3.64	7.07	30	1
9	0	30	128	8.53	1.59	10	5	0.28	0.05	0.4	0.16	0	1.76	0.53	3	1
10	0	30	131	8.73	1.29	10	5	0.27	0.06	0.39	0.14	0	1.75	0.54	3	1
11	0	30	129	8.6	1.5	10	5	0.26	0.07	0.4	0.1	0	1.73	0.51	3	1
12	0	30	124	8.27	1.65	10	4	0.24	0.05	0.33	0.17	0	1.74	0.5	3	1
13	0	30	128	8.53	1.45	10	5	0.27	0.07	0.4	0.11	0	1.75	0.52	3	1
14	0	30	126	8.4	1.82	10	3	0.3	0.1	0.67	0.13	0	1.77	0.54	3	1
15	0	30	126	8.4	1.38	10	5	0.22	0.07	0.36	0.05	0	1.76	0.53	3	1
16	0	30	130	8.67	1.37	10	5	0.26	0.07	0.43	0.13	0	1.74	0.51	3	1
17	0	30	131	8.73	1.44	10	6	0.27	0.05	0.38	0.14	0	1.72	0.5	3	1
18	0	30	130	8.67	1.51	10	4	0.29	0.06	0.42	0.17	0	1.73	0.5	3	1
19	0	30	131	8.73	1.44	10	4	0.27	0.06	0.36	0.13	0	1.74	0.52	3	1
20	0	30	130	8.67	1.4	10	5	0.24	0.08	0.38	0.07	0	1.72	0.49	3	1

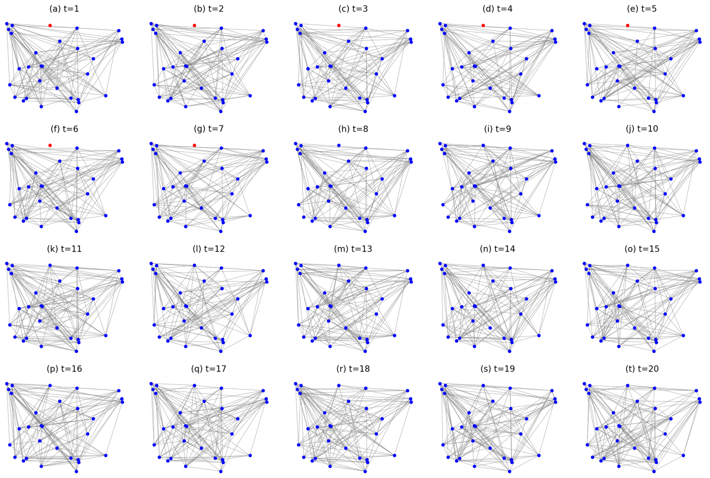

Fig A. The evolving social networks driven by inactive nodes in an epidemic outbreak.

Ignorant

Table B. Topological information of the network simulations driven by ignorant mutation style under a social capital limit at 10.

Iteration	Nodes		Edges	Node Degree				Clustering coefficient				Shortest path length				
	Connected	Unconnected		Avg.	Std.	Max.	Min.	Avg.	Std.	Max.	Min.	Fake Paths	Avg.	Std.	Max.	Min.
0	30	0	0	0.00	0.00	0	0	0.00	0.00	0	0	435	30.00	0.00	30	30
1	1	29	121	8.07	2.1	10	0	0.26	0.08	0.4	0.00	29	3.63	7.07	30	1
2	5	25	74	4.93	3.71	10	0	0.18	0.23	0.67	0.00	135	10.95	12.82	30	1
3	3	27	75	5.0	3.46	10	0	0.41	0.33	1.00	0.00	84	7.94	10.86	30	1
4	1	29	85	5.67	3.13	10	0	0.29	0.33	1.00	0.00	29	4.1	6.97	30	1
5	4	26	78	5.2	3.55	10	0	0.24	0.3	1.00	0.00	179	13.47	13.83	30	1
6	4	26	87	5.8	3.71	10	0	0.21	0.23	1.00	0.00	110	9.14	12.16	30	1
7	4	26	76	5.07	3.69	10	0	0.32	0.26	0.7	0.00	110	9.21	12.12	30	1
8	2	28	87	5.8	2.93	10	0	0.35	0.23	1.00	0.00	57	5.77	9.44	30	1
9	5	25	78	5.2	3.73	10	0	0.31	0.27	1.00	0.00	135	10.77	12.92	30	1
10	6	24	86	5.73	3.82	10	0	0.19	0.17	0.47	0.00	159	12.16	13.55	30	1
11	5	25	83	5.53	3.86	10	0	0.16	0.14	0.4	0.00	135	10.7	12.96	30	1
12	2	28	91	6.07	3.17	10	0	0.26	0.29	1.00	0.00	57	5.71	9.46	30	1
13	2	28	85	5.67	3.33	10	0	0.35	0.33	1.00	0.00	57	6.03	9.36	30	1
14	5	25	83	5.53	3.65	10	0	0.24	0.25	1.00	0.00	135	10.77	12.93	30	1
15	5	25	78	5.2	3.77	10	0	0.27	0.23	0.67	0.00	135	10.78	12.91	30	1
16	5	25	80	5.33	3.79	10	0	0.22	0.25	1.00	0.00	135	10.74	12.94	30	1
17	7	23	69	4.6	4.14	10	0	0.28	0.29	1.00	0.00	278	19.78	13.6	30	1
18	8	22	79	5.27	4.07	10	0	0.24	0.26	1.00	0.00	204	15.04	14.07	30	1
19	4	26	83	5.53	3.73	10	0	0.27	0.31	1.00	0.00	158	12.19	13.47	30	1
20	5	25	83	5.53	3.69	10	0	0.2	0.22	0.7	0.00	135	10.75	12.93	30	1

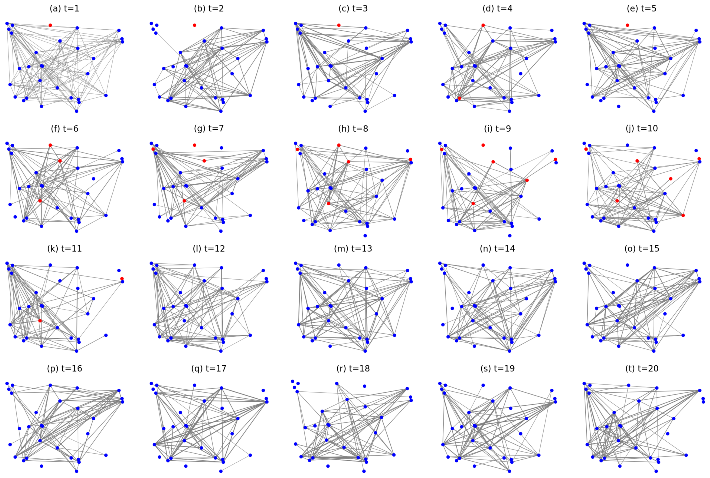

Fig B. The evolving social networks driven by ignorant nodes in an epidemic outbreak.

Egocentric

Table C. Topological information of the network simulations driven by egocentric mutation style under a social capital limit at 10.

Iteration	Nodes		Edges	Node Degree				Clustering coefficient				Shortest path length				
	Connected	Unconnected		Avg.	Std.	Max.	Min.	Avg.	Std.	Max.	Min.	Fake Paths	Avg.	Std.	Max.	Min.
0	30	0	0	0.00	0.00	0	0	0.00	0.00	0	0	435	30.00	0.00	30	30
1	1	29	121	8.07	2.1	10	0	0.26	0.08	0.4	0.00	29	3.63	7.07	30	1
2	7	23	77	5.13	3.7	10	0	0.17	0.16	0.51	0.00	182	13.67	13.86	30	1
3	3	27	73	4.87	3.59	10	0	0.34	0.29	1.00	0.00	134	10.81	12.83	30	1
4	2	28	79	5.27	3.43	10	0	0.31	0.32	1.00	0.00	57	6.13	9.34	30	1
5	3	27	94	6.27	3.41	10	0	0.22	0.25	0.8	0.00	84	7.58	11.01	30	1
6	1	29	94	6.27	3.38	10	0	0.21	0.3	1.00	0.00	29	4.08	6.98	30	1
7	2	28	97	6.47	3.15	10	0	0.19	0.27	1.00	0.00	57	5.74	9.45	30	1
8	2	28	104	6.93	3.17	10	0	0.46	0.26	0.93	0.00	57	5.72	9.46	30	1
9	1	29	87	5.8	3.61	10	0	0.5	0.33	1.00	0.00	83	7.69	10.88	30	1
10	1	29	100	6.67	2.74	10	0	0.42	0.27	1.00	0.00	29	4.03	6.99	30	1
11	1	29	106	7.07	2.9	10	0	0.39	0.21	0.8	0.00	29	4.0	7.0	30	1
12	1	29	100	6.67	3.08	10	0	0.41	0.26	0.9	0.00	29	4.07	6.99	30	1
13	0	30	103	6.87	2.42	10	2	0.25	0.25	1.00	0.00	0	2.11	0.81	5	1
14	4	26	92	6.13	3.39	10	0	0.25	0.25	1.00	0.00	110	9.09	12.19	30	1
15	1	29	98	6.53	3.24	10	0	0.19	0.24	1.00	0.00	29	4.05	6.99	30	1
16	0	30	96	6.4	2.92	10	3	0.33	0.22	0.69	0.00	0	2.27	0.93	4	1
17	3	27	102	6.8	2.9	10	0	0.41	0.23	0.73	0.00	84	7.49	11.04	30	1
18	2	28	107	7.13	2.96	10	0	0.44	0.32	1.00	0.00	57	5.77	9.44	30	1
19	0	30	111	7.4	2.64	10	1	0.49	0.23	1.00	0.00	0	2.17	0.89	4	1
20	2	28	95	6.33	3.14	10	0	0.41	0.24	0.9	0.00	57	5.86	9.42	30	1

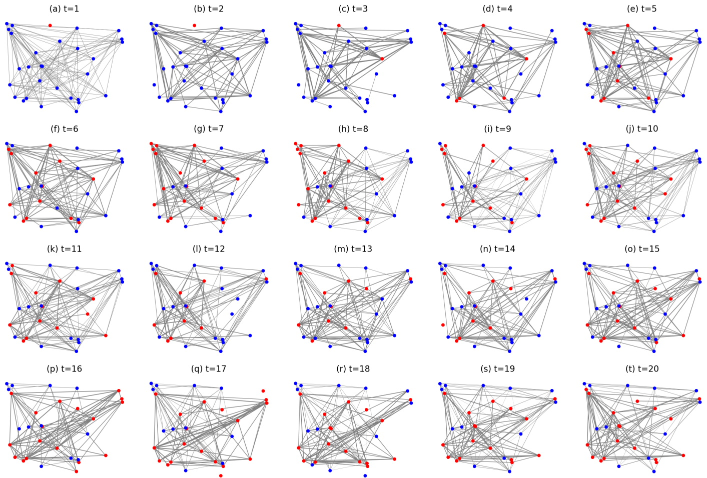

Fig C. The evolving social networks driven by egocentric nodes in an epidemic outbreak.

Cooperative

Table D. Topological information of the network simulations driven by cooperative mutation style under a social capital limit at 10.

Iteration	Nodes		Edges	Node Degree				Clustering coefficient				Shortest path length				
	Connected	Unconnected		Avg.	Std.	Max.	Min.	Avg.	Std.	Max.	Min.	Fake Paths	Avg.	Std.	Max.	Min.
0	30	0	0	0.00	0.00	0	0	0.00	0.00	0	0	435	30.00	0.00	30	30
1	1	29	121	8.07	2.1	10	0	0.26	0.08	0.4	0.00	29	3.63	7.07	30	1
2	8	22	74	4.93	3.8	10	0	0.28	0.27	1.00	0.00	204	15.09	14.02	30	1
3	3	27	85	5.67	3.51	10	0	0.31	0.28	1.00	0.00	84	7.6	10.99	30	1
4	4	26	77	5.13	3.48	10	0	0.29	0.28	1.00	0.00	110	9.26	12.1	30	1
5	4	26	80	5.33	3.59	10	0	0.24	0.28	0.79	0.00	110	9.23	12.11	30	1
6	4	26	76	5.07	3.62	10	0	0.27	0.31	1.00	0.00	110	9.3	12.07	30	1
7	4	26	77	5.13	3.69	10	0	0.29	0.28	0.78	0.00	110	9.32	12.06	30	1
8	7	23	79	5.27	4.04	10	0	0.29	0.25	0.8	0.00	224	16.31	14.11	30	1
9	11	19	70	4.67	4.38	10	0	0.27	0.29	0.79	0.00	298	21.04	13.22	30	1
10	9	21	73	4.87	4.19	10	0	0.27	0.3	0.87	0.00	225	16.43	14.06	30	1
11	4	26	77	5.13	3.53	10	0	0.31	0.26	1.00	0.00	110	9.17	12.14	30	1
12	7	23	76	5.07	4.17	10	0	0.25	0.25	0.71	0.00	242	17.47	14.04	30	1
13	3	27	84	5.6	3.51	10	0	0.43	0.3	1.00	0.00	84	7.58	11.00	30	1
14	4	26	94	6.27	3.3	10	0	0.22	0.22	1.00	0.00	110	8.99	12.24	30	1
15	4	26	78	5.2	3.57	10	0	0.34	0.27	1.00	0.00	158	12.25	13.43	30	1
16	9	21	70	4.67	4.16	10	0	0.29	0.29	1.00	0.00	225	16.5	13.99	30	1
17	5	25	83	5.53	3.47	10	0	0.32	0.27	1.00	0.00	135	10.69	12.97	30	1
18	9	21	81	5.4	4.0	10	0	0.39	0.29	1.00	0.00	225	16.36	14.13	30	1
19	5	25	78	5.2	3.75	10	0	0.33	0.26	1.00	0.00	181	13.59	13.86	30	1
20	5	25	88	5.87	3.66	10	0	0.16	0.18	0.5	0.00	135	10.64	13.0	30	1

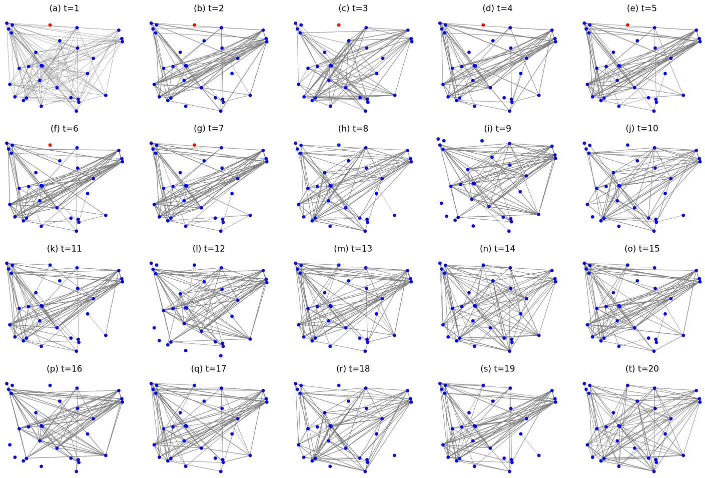

Fig D. The evolving social networks driven by cooperative nodes in an epidemic outbreak.

Collaborative

Table E. Topological information of the network simulations driven by collaborative mutation style under a social capital limit at 10.

Iteration	Nodes		Edges	Node Degree				Clustering coefficient				Shortest path length				
	Connected	Unconnected		Avg.	Std.	Max.	Min.	Avg.	Std.	Max.	Min.	Fake Paths	Avg.	Std.	Max.	Min.
0	30	0	0	0.00	0.00	0	0	0.00	0.00	0	0	435	30.00	0.00	30	30
1	1	29	225	15.0	4.18	26	0	0.56	0.11	0.67	0.00	29	3.35	7.14	30	1
2	1	29	214	14.27	5.09	28	0	0.59	0.13	0.89	0.00	29	3.37	7.13	30	1
3	1	29	211	14.07	5.16	28	0	0.59	0.13	0.79	0.00	29	3.38	7.13	30	1
4	1	29	216	14.4	5.14	28	0	0.59	0.12	0.72	0.00	29	3.37	7.13	30	1
5	1	29	213	14.2	5.06	28	0	0.59	0.12	0.69	0.00	29	3.38	7.13	30	1
6	1	29	213	14.2	5.26	28	0	0.6	0.13	0.76	0.00	29	3.38	7.13	30	1
7	0	30	220	14.67	4.64	25	1	0.57	0.13	0.75	0.00	0	1.53	0.57	3	1
8	1	29	222	14.8	4.84	24	0	0.59	0.13	0.73	0.00	29	3.36	7.14	30	1
9	0	30	214	14.27	3.34	20	8	0.51	0.06	0.65	0.33	0	1.51	0.5	2	1
10	0	30	229	15.27	2.95	22	11	0.53	0.05	0.68	0.45	0	1.47	0.5	2	1
11	0	30	217	14.47	4.17	26	7	0.58	0.09	0.93	0.45	0	1.5	0.5	2	1
12	0	30	235	15.67	3.32	22	8	0.57	0.05	0.71	0.49	0	1.46	0.5	2	1
13	0	30	227	15.13	3.63	22	7	0.55	0.06	0.69	0.41	0	1.48	0.5	2	1
14	0	30	221	14.73	3.3	22	9	0.53	0.05	0.65	0.44	0	1.49	0.5	2	1
15	0	30	219	14.6	3.37	20	6	0.53	0.05	0.64	0.42	0	1.5	0.5	2	1
16	0	30	234	15.6	3.01	23	6	0.54	0.04	0.62	0.47	0	1.46	0.5	2	1
17	0	30	227	15.13	3.32	21	6	0.55	0.04	0.64	0.49	0	1.48	0.5	2	1
18	0	30	230	15.33	3.63	24	9	0.57	0.08	0.81	0.45	0	1.47	0.5	2	1
19	0	30	215	14.33	3.21	19	6	0.5	0.07	0.58	0.27	0	1.51	0.5	2	1
20	0	30	219	14.6	3.27	21	7	0.5	0.07	0.64	0.31	0	1.5	0.5	2	1

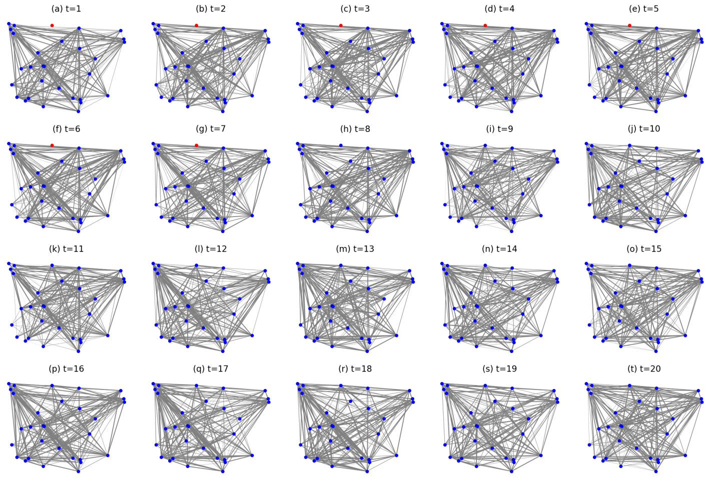

Fig E. The evolving social networks driven by collaborative nodes in an epidemic outbreak.
