# Supplementary material for: Evolutionary Digital Twin-Oriented Complex Networked Systems driven by node features and the mutation of feature preferences
Source: PLoS One. 2024 May 16;19(5):e0303571. doi: 10.1371/journal.pone.0303571 (PMC11098356; doi:10.1371/journal.pone.0303571)
Supplement: S3 Appendix — (PDF) [file pone.0303571.s003.pdf]

Social network simulations over twenty iterations based on an unconnected backbone network under social capital limit at 15

In this appendix, we present the dynamic social networks generated over twenty iterations considering different social DNA mutation styles, under a social capital limit at 15.

Inactive

Table A. Topological information of the network simulations driven by inactive mutation style under a social capital limit at 5.

Iteration	Nodes		Edges	Node Degree				Clustering coefficient				Shortest path length				
	Connected	Unconnected		Avg.	Std.	Max.	Min.	Avg.	Std.	Max.	Min.	Fake Paths	Avg.	Std.	Max.	Min.
0	30	0	0	0.00	0.00	0	0	0.00	0.00	0	0	435	30.00	0.00	30	30
1	1	29	192	12.8	2.87	15	0	0.46	0.09	0.58	0.00	29	3.43	7.12	30	1
2	1	29	199	13.27	2.66	15	0	0.45	0.09	0.54	0.00	29	3.41	7.12	30	1
3	1	29	191	12.73	3.12	15	0	0.43	0.09	0.56	0.00	29	3.43	7.12	30	1
4	1	29	192	12.8	2.91	15	0	0.44	0.09	0.55	0.00	29	3.43	7.12	30	1
5	1	29	195	13.0	3.06	15	0	0.44	0.09	0.53	0.00	29	3.42	7.12	30	1
6	1	29	196	13.07	2.83	15	0	0.45	0.09	0.51	0.00	29	3.42	7.12	30	1
7	1	29	194	12.93	2.89	15	0	0.44	0.09	0.52	0.00	29	3.42	7.12	30	1
8	1	29	194	12.93	2.78	15	0	0.44	0.09	0.53	0.00	29	3.42	7.12	30	1
9	0	30	201	13.4	1.74	15	8	0.44	0.03	0.52	0.37	0	1.54	0.5	2	1
10	0	30	200	13.33	1.97	15	7	0.43	0.05	0.53	0.33	0	1.54	0.5	2	1
11	0	30	202	13.47	1.43	15	9	0.45	0.03	0.5	0.36	0	1.54	0.5	2	1
12	0	30	200	13.33	1.35	15	10	0.42	0.03	0.47	0.37	0	1.54	0.5	2	1
13	0	30	207	13.8	1.05	15	11	0.45	0.03	0.54	0.38	0	1.52	0.5	2	1
14	0	30	196	13.07	1.81	15	7	0.42	0.04	0.55	0.33	0	1.55	0.5	2	1
15	0	30	199	13.27	1.59	15	9	0.42	0.03	0.48	0.36	0	1.54	0.5	2	1
16	0	30	207	13.8	1.6	15	8	0.45	0.04	0.51	0.36	0	1.52	0.5	2	1
17	0	30	199	13.27	1.53	15	10	0.43	0.04	0.51	0.33	0	1.54	0.5	2	1
18	0	30	207	13.8	1.28	15	11	0.45	0.03	0.52	0.38	0	1.52	0.5	2	1
19	0	30	207	13.8	1.3	15	11	0.46	0.03	0.55	0.42	0	1.52	0.5	2	1
20	0	30	207	13.8	1.62	15	9	0.46	0.04	0.55	0.35	0	1.52	0.5	2	1

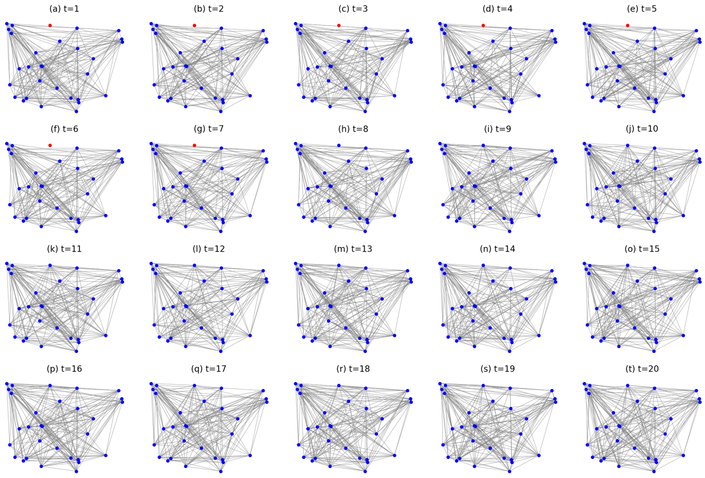

Fig A. The evolving social networks driven by inactive nodes in an epidemic outbreak.

Ignorant

Table B. Topological information of the network simulations driven by ignorant mutation style under a social capital limit at 15.

Iteration	Nodes		Edges	Node Degree				Clustering coefficient				Shortest path length				
	Connected	Unconnected		Avg.	Std.	Max.	Min.	Avg.	Std.	Max.	Min.	Fake Paths	Avg.	Std.	Max.	Min.
0	30	0	0	0.00	0.00	0	0	0.00	0.00	0	0	435	30.00	0.00	30	30
1	1	29	192	12.8	2.87	15	0	0.46	0.09	0.58	0.00	29	3.43	7.12	30	1
2	2	28	137	9.13	5.44	15	0	0.51	0.28	1.00	0.00	57	5.6	9.51	30	1
3	1	29	143	9.53	4.89	15	0	0.47	0.21	0.83	0.00	29	3.66	7.07	30	1
4	0	30	145	9.67	4.29	15	1	0.48	0.26	1.00	0.00	0	1.84	0.73	4	1
5	2	28	141	9.4	5.01	15	0	0.48	0.25	1.00	0.00	57	5.48	9.55	30	1
6	0	30	151	10.07	4.5	15	1	0.35	0.2	0.76	0.00	0	1.76	0.63	4	1
7	1	29	135	9.0	5.1	15	0	0.49	0.24	1.00	0.00	29	3.71	7.06	30	1
8	0	30	148	9.87	4.22	15	1	0.47	0.24	1.00	0.00	0	1.78	0.64	4	1
9	2	28	136	9.07	5.46	15	0	0.53	0.28	1.00	0.00	57	5.46	9.55	30	1
10	4	26	128	8.53	5.55	15	0	0.5	0.25	1.00	0.00	110	8.85	12.32	30	1
11	4	26	127	8.47	5.63	15	0	0.51	0.31	1.00	0.00	110	8.92	12.28	30	1
12	5	25	128	8.53	5.91	15	0	0.5	0.34	1.00	0.00	135	10.49	13.1	30	1
13	3	27	145	9.67	5.24	15	0	0.5	0.24	1.00	0.00	84	7.14	11.2	30	1
14	4	26	128	8.53	5.58	15	0	0.45	0.29	1.00	0.00	110	8.87	12.31	30	1
15	2	28	144	9.6	4.72	15	0	0.5	0.23	1.00	0.00	57	5.43	9.56	30	1
16	2	28	125	8.33	5.41	15	0	0.54	0.31	1.00	0.00	57	5.56	9.51	30	1
17	5	25	132	8.8	5.05	15	0	0.57	0.3	1.00	0.00	135	10.44	13.13	30	1
18	3	27	121	8.07	4.77	15	0	0.53	0.33	1.00	0.00	84	7.27	11.14	30	1
19	4	26	99	6.6	4.62	15	0	0.42	0.31	1.00	0.00	110	8.99	12.24	30	1
20	3	27	91	6.07	4.09	15	0	0.4	0.32	1.00	0.00	84	7.4	11.08	30	1

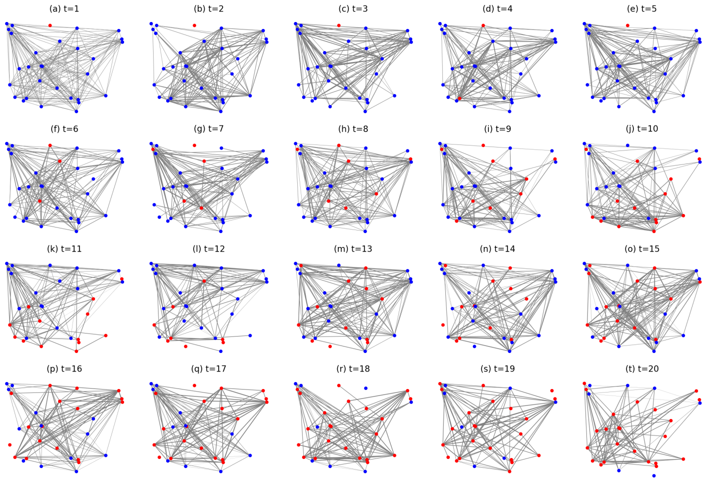

Fig B. The evolving social networks driven by ignorant nodes in an epidemic outbreak.

Egocentric

Table C. Topological information of the network simulations driven by egocentric mutation style under a social capital limit at 15.

Iteration	Nodes		Edges	Node Degree				Clustering coefficient				Shortest path length				
	Connected	Unconnected		Avg.	Std.	Max.	Min.	Avg.	Std.	Max.	Min.	Fake Paths	Avg.	Std.	Max.	Min.
0	30	0	0	0.00	0.00	0	0	0.00	0.00	0	0	435	30.00	0.00	30	30
1	1	29	192	12.8	2.87	15	0	0.46	0.09	0.58	0.00	29	3.43	7.12	30	1
2	5	25	139	9.27	6.02	15	0	0.36	0.31	0.79	0.00	135	10.43	13.14	30	1
3	0	30	146	9.73	5.3	15	1	0.58	0.29	1.00	0.00	0	1.91	0.77	4	1
4	1	29	147	9.8	5.22	15	0	0.45	0.31	0.8	0.00	29	3.71	7.06	30	1
5	2	28	165	11.00	4.5	15	0	0.4	0.2	1.00	0.00	57	5.34	9.59	30	1
6	0	30	160	10.67	4.17	15	4	0.65	0.27	1.00	0.14	0	1.87	0.77	4	1
7	2	28	135	9.0	5.0	15	0	0.63	0.35	1.00	0.00	57	5.59	9.51	30	1
8	2	28	139	9.27	4.33	15	0	0.64	0.3	1.00	0.00	57	5.59	9.51	30	1
9	3	27	128	8.53	4.73	15	0	0.69	0.31	1.00	0.00	84	7.89	10.91	30	1
10	1	29	146	9.73	4.77	15	0	0.68	0.28	1.00	0.00	29	4.13	7.01	30	1
11	1	29	148	9.87	4.54	15	0	0.57	0.21	0.8	0.00	29	3.82	7.05	30	1
12	0	30	152	10.13	3.97	15	2	0.59	0.2	1.00	0.21	0	1.8	0.68	3	1
13	0	30	153	10.2	3.67	15	3	0.51	0.19	1.00	0.27	0	1.8	0.69	4	1
14	1	29	155	10.33	3.67	15	0	0.45	0.19	1.00	0.00	29	3.59	7.08	30	1
15	1	29	160	10.67	3.88	15	0	0.46	0.17	0.83	0.00	29	3.56	7.09	30	1
16	0	30	148	9.87	4.19	15	1	0.66	0.23	1.00	0.00	0	1.91	0.79	4	1
17	2	28	136	9.07	4.15	15	0	0.53	0.25	1.00	0.00	57	5.59	9.51	30	1
18	2	28	144	9.6	4.42	15	0	0.48	0.27	1.00	0.00	57	5.51	9.53	30	1
19	1	29	150	10.00	4.43	15	0	0.38	0.23	1.00	0.00	29	3.69	7.07	30	1
20	2	28	121	8.07	4.05	15	0	0.44	0.29	1.00	0.00	57	5.59	9.5	30	1

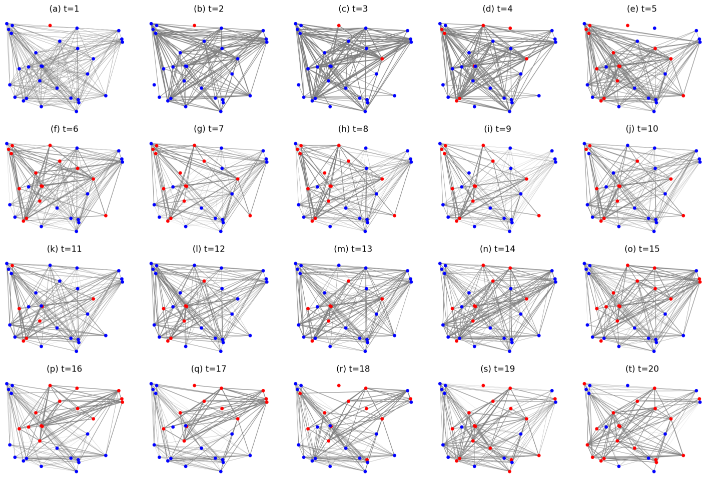

Fig C. The evolving social networks driven by egocentric nodes in an epidemic outbreak.

Cooperative

Table D. Topological information of the network simulations driven by cooperative mutation style under a social capital limit at 15.

Iteration	Nodes		Edges	Node Degree				Clustering coefficient				Shortest path length				
	Connected	Unconnected		Avg.	Std.	Max.	Min.	Avg.	Std.	Max.	Min.	Fake Paths	Avg.	Std.	Max.	Min.
0	30	0	0	0.00	0.00	0	0	0.00	0.00	0	0	435	30.00	0.00	30	30
1	1	29	192	12.8	2.87	15	0	0.46	0.09	0.58	0.00	29	3.43	7.12	30	1
2	4	26	145	9.67	5.42	15	0	0.5	0.26	1.00	0.00	110	8.83	12.33	30	1
3	1	29	151	10.07	4.75	15	0	0.44	0.24	1.00	0.00	29	3.63	7.08	30	1
4	2	28	147	9.8	5.1	15	0	0.46	0.22	0.67	0.00	57	5.42	9.56	30	1
5	2	28	150	10.00	5.22	15	0	0.46	0.3	1.00	0.00	57	5.45	9.56	30	1
6	1	29	155	10.33	5.21	15	0	0.47	0.27	0.76	0.00	29	3.75	7.06	30	1
7	1	29	151	10.07	5.05	15	0	0.46	0.29	0.83	0.00	29	3.72	7.07	30	1
8	1	29	153	10.2	4.84	15	0	0.45	0.24	1.00	0.00	29	3.62	7.08	30	1
9	1	29	140	9.33	5.88	15	0	0.42	0.32	0.78	0.00	29	3.93	7.03	30	1
10	0	30	147	9.8	5.53	15	1	0.5	0.38	1.00	0.00	0	2.07	0.93	4	1
11	0	30	159	10.6	4.24	15	2	0.48	0.22	1.00	0.00	0	1.77	0.67	4	1
12	4	26	136	9.07	6.26	15	0	0.46	0.35	0.86	0.00	110	9.05	12.22	30	1
13	0	30	154	10.27	4.69	15	1	0.5	0.23	1.00	0.00	0	1.83	0.74	4	1
14	3	27	140	9.33	6.02	15	0	0.52	0.34	1.00	0.00	84	7.26	11.15	30	1
15	0	30	159	10.6	4.69	15	1	0.48	0.23	0.93	0.00	56	5.31	9.51	30	1
16	4	26	135	9.0	6.03	15	0	0.5	0.31	0.84	0.00	110	8.97	12.26	30	1
17	1	29	156	10.4	4.64	15	0	0.53	0.22	1.00	0.00	29	3.65	7.08	30	1
18	6	24	136	9.07	6.45	15	0	0.5	0.36	1.00	0.00	159	12.02	13.66	30	1
19	1	29	136	9.07	6.03	15	0	0.47	0.4	0.92	0.00	29	4.17	7.01	30	1
20	0	30	159	10.6	4.63	15	1	0.51	0.23	1.00	0.00	0	1.84	0.8	4	1

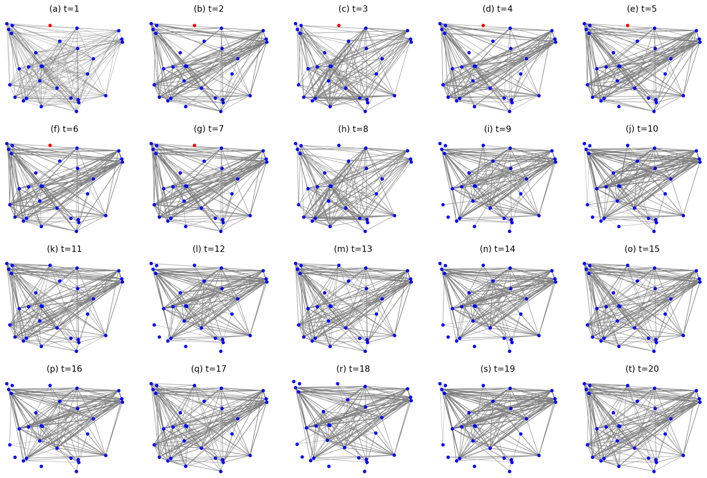

Fig D. The evolving social networks driven by cooperative nodes in an epidemic outbreak.

Collaborative

Table E. Topological information of the network simulations driven by collaborative mutation style under a social capital limit at 15.

Iteration	Nodes		Edges	Node Degree				Clustering coefficient				Shortest path length				
	Connected	Unconnected		Avg.	Std.	Max.	Min.	Avg.	Std.	Max.	Min.	Fake Paths	Avg.	Std.	Max.	Min.
0	30	0	0	0.00	0.00	0	0	0.00	0.00	0	0	435	30.00	0.00	30	30
1	1	29	302	20.13	4.45	26	0	0.72	0.14	0.8	0.00	29	3.17	7.18	30	1
2	1	29	284	18.93	5.4	28	0	0.73	0.15	0.95	0.00	29	3.21	7.17	30	1
3	1	29	290	19.33	5.09	28	0	0.72	0.14	0.83	0.00	29	3.2	7.18	30	1
4	1	29	282	18.8	5.35	28	0	0.73	0.14	0.88	0.00	29	3.22	7.17	30	1
5	1	29	290	19.33	4.96	28	0	0.71	0.14	0.79	0.00	29	3.2	7.18	30	1
6	0	30	279	18.6	5.23	28	1	0.71	0.14	0.81	0.00	0	1.37	0.51	3	1
7	1	29	283	18.87	5.45	28	0	0.74	0.15	0.88	0.00	29	3.22	7.17	30	1
8	0	30	276	18.4	5.29	29	1	0.7	0.14	0.85	0.00	0	1.37	0.48	2	1
9	0	30	287	19.13	4.78	29	8	0.73	0.07	0.86	0.64	0	1.34	0.47	2	1
10	0	30	286	19.07	4.17	25	10	0.71	0.06	0.82	0.61	0	1.34	0.47	2	1
11	0	30	303	20.2	3.39	28	11	0.72	0.04	0.79	0.66	0	1.3	0.46	2	1
12	0	30	277	18.47	4.59	29	7	0.71	0.07	0.9	0.59	0	1.36	0.48	2	1
13	0	30	300	20.00	3.56	26	12	0.71	0.04	0.82	0.65	0	1.31	0.46	2	1
14	0	30	278	18.53	4.84	29	7	0.72	0.07	0.9	0.59	0	1.36	0.48	2	1
15	0	30	280	18.67	5.37	29	8	0.76	0.09	0.93	0.62	0	1.36	0.48	2	1
16	0	30	264	17.6	4.83	29	8	0.67	0.07	0.78	0.51	0	1.39	0.49	2	1
17	0	30	303	20.2	4.69	29	11	0.76	0.07	0.93	0.65	0	1.3	0.46	2	1
18	0	30	288	19.2	4.45	28	10	0.72	0.05	0.85	0.64	0	1.34	0.47	2	1
19	0	30	288	19.2	4.76	29	6	0.74	0.07	1.00	0.64	0	1.34	0.47	2	1
20	0	30	276	18.4	4.39	26	8	0.69	0.07	0.83	0.56	0	1.37	0.48	2	1

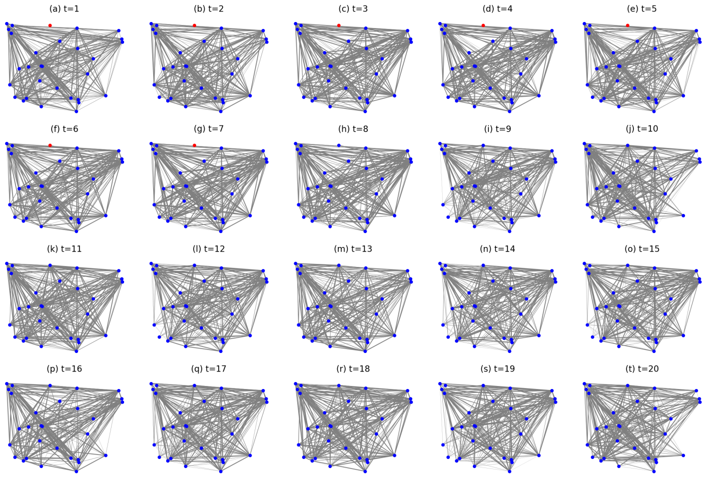

Fig E. The evolving social networks driven by collaborative nodes in an epidemic outbreak.
