# Supplementary material for: Evolutionary Digital Twin-Oriented Complex Networked Systems driven by node features and the mutation of feature preferences
Source: PLoS One. 2024 May 16;19(5):e0303571. doi: 10.1371/journal.pone.0303571 (PMC11098356; doi:10.1371/journal.pone.0303571)
Supplement: S4 Appendix — (PDF) [file pone.0303571.s004.pdf]

Social network simulations over twenty iterations based on an unconnected backbone network under social capital limit at 20

In this appendix, we present the dynamic social networks generated over twenty iterations considering different social DNA mutation styles, under a social capital limit at 20.

Inactive

Table A. Topological information of the network simulations driven by inactive mutation style under a social capital limit at 5.

Iteration	Nodes		Edges	Node Degree				Clustering coefficient				Shortest path length				
	Connected	Unconnected		Avg.	Std.	Max.	Min.	Avg.	Std.	Max.	Min.	Fake Paths	Avg.	Std.	Max.	Min.
0	30	0	0	0.00	0.00	0	0	0.00	0.00	0	0	435	30.00	0.00	30	30
1	1	29	268	17.87	3.73	20	0	0.63	0.12	0.71	0.00	29	3.25	7.16	30	1
2	1	29	271	18.07	3.68	20	0	0.63	0.12	0.68	0.00	29	3.24	7.17	30	1
3	1	29	266	17.73	3.84	20	0	0.63	0.12	0.69	0.00	29	3.26	7.16	30	1
4	1	29	272	18.13	3.67	20	0	0.64	0.12	0.69	0.00	29	3.24	7.17	30	1
5	1	29	274	18.27	3.6	20	0	0.64	0.12	0.69	0.00	29	3.24	7.17	30	1
6	1	29	271	18.07	3.68	20	0	0.64	0.12	0.73	0.00	29	3.24	7.17	30	1
7	1	29	273	18.2	3.68	20	0	0.64	0.12	0.72	0.00	29	3.24	7.17	30	1
8	1	29	264	17.6	3.59	20	0	0.61	0.12	0.7	0.00	29	3.26	7.16	30	1
9	0	30	282	18.8	1.47	20	15	0.63	0.02	0.67	0.6	0	1.35	0.48	2	1
10	0	30	275	18.33	2.13	20	11	0.63	0.03	0.68	0.55	0	1.37	0.48	2	1
11	0	30	281	18.73	1.39	20	15	0.63	0.01	0.66	0.6	0	1.35	0.48	2	1
12	0	30	280	18.67	1.6	20	14	0.63	0.02	0.67	0.57	0	1.36	0.48	2	1
13	0	30	282	18.8	1.08	20	17	0.62	0.02	0.66	0.57	0	1.35	0.48	2	1
14	0	30	278	18.53	1.54	20	13	0.63	0.02	0.67	0.55	0	1.36	0.48	2	1
15	0	30	280	18.67	1.51	20	14	0.63	0.02	0.66	0.59	0	1.36	0.48	2	1
16	0	30	279	18.6	1.4	20	15	0.62	0.02	0.66	0.57	0	1.36	0.48	2	1
17	0	30	279	18.6	1.47	20	15	0.63	0.02	0.67	0.57	0	1.36	0.48	2	1
18	0	30	278	18.53	1.73	20	13	0.62	0.03	0.68	0.57	0	1.36	0.48	2	1
19	0	30	281	18.73	1.41	20	15	0.64	0.02	0.68	0.6	0	1.35	0.48	2	1
20	0	30	280	18.67	1.3	20	16	0.63	0.02	0.67	0.59	0	1.36	0.48	2	1

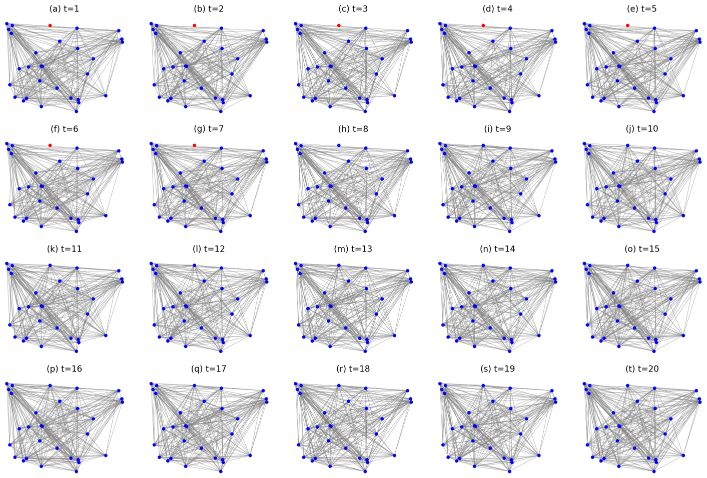

Fig A. The evolving social networks driven by inactive nodes in an epidemic outbreak.

Ignorant

Table B. Topological information of the network simulations driven by ignorant mutation style under a social capital limit at 20.

Iteration	Nodes		Edges	Node Degree				Clustering coefficient				Shortest path length				
	Connected	Unconnected		Avg.	Std.	Max.	Min.	Avg.	Std.	Max.	Min.	Fake Paths	Avg.	Std.	Max.	Min.
0	30	0	0	0.00	0.00	0	0	0.00	0.00	0	0	435	30.00	0.00	30	30
1	1	29	268	17.87	3.73	20	0	0.63	0.12	0.71	0.00	29	3.25	7.16	30	1
2	1	29	216	14.4	6.48	20	0	0.64	0.24	0.97	0.00	29	3.42	7.13	30	1
3	1	29	223	14.87	6.06	20	0	0.67	0.18	1.00	0.00	29	3.38	7.13	30	1
4	0	30	227	15.13	4.91	20	4	0.64	0.13	1.00	0.39	0	1.5	0.54	3	1
5	1	29	225	15.0	5.54	20	0	0.64	0.18	1.00	0.00	29	3.38	7.13	30	1
6	0	30	229	15.27	5.35	20	2	0.59	0.15	1.00	0.2	0	1.51	0.56	3	1
7	1	29	220	14.67	6.26	20	0	0.65	0.21	1.00	0.00	29	3.38	7.13	30	1
8	0	30	226	15.07	5.14	20	5	0.64	0.14	1.00	0.2	0	1.5	0.54	3	1
9	3	27	216	14.4	7.0	20	0	0.71	0.25	1.00	0.00	84	6.93	11.3	30	1
10	4	26	198	13.2	7.62	20	0	0.72	0.3	1.00	0.00	110	8.64	12.44	30	1
11	4	26	154	10.27	6.65	20	0	0.62	0.34	1.00	0.00	110	8.8	12.35	30	1
12	4	26	147	9.8	6.92	20	0	0.56	0.4	1.00	0.00	110	8.81	12.34	30	1
13	2	28	216	14.4	7.05	20	0	0.65	0.28	0.96	0.00	57	5.22	9.64	30	1
14	2	28	170	11.33	6.9	20	0	0.63	0.33	1.00	0.00	57	5.37	9.59	30	1
15	2	28	171	11.4	5.66	20	0	0.57	0.29	1.00	0.00	57	5.31	9.6	30	1
16	4	26	87	5.8	4.97	19	0	0.52	0.38	1.00	0.00	110	8.99	12.23	30	1
17	5	25	84	5.6	4.18	17	0	0.47	0.35	1.00	0.00	135	10.66	12.99	30	1
18	6	24	87	5.8	4.04	13	0	0.42	0.36	1.00	0.00	159	12.14	13.57	30	1
19	4	26	99	6.6	5.0	20	0	0.5	0.34	1.00	0.00	110	8.94	12.26	30	1
20	4	26	101	6.73	4.6	20	0	0.51	0.33	1.00	0.00	110	8.95	12.26	30	1

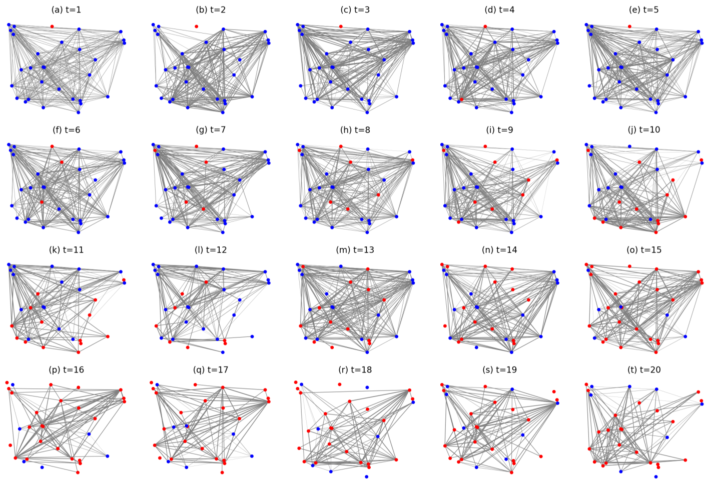

Fig B. The evolving social networks driven by ignorant nodes in an epidemic outbreak.

Egocentric

Table C. Topological information of the network simulations driven by egocentric mutation style under a social capital limit at 20.

Iteration	Nodes		Edges	Node Degree				Clustering coefficient				Shortest path length				
	Connected	Unconnected		Avg.	Std.	Max.	Min.	Avg.	Std.	Max.	Min.	Fake Paths	Avg.	Std.	Max.	Min.
0	30	0	0	0.00	0.00	0	0	0.00	0.00	0	0	435	30.00	0.00	30	30
1	1	29	268	17.87	3.73	20	0	0.63	0.12	0.71	0.00	29	3.25	7.16	30	1
2	3	27	215	14.33	7.61	20	0	0.62	0.31	0.88	0.00	84	6.94	11.29	30	1
3	0	30	224	14.93	7.03	20	1	0.63	0.35	0.91	0.00	0	1.6	0.69	4	1
4	0	30	232	15.47	5.64	20	4	0.59	0.24	0.85	0.13	0	1.51	0.57	3	1
5	3	27	220	14.67	6.77	20	0	0.67	0.25	0.94	0.00	84	6.91	11.3	30	1
6	1	29	143	9.53	5.04	20	0	0.6	0.3	1.00	0.00	29	3.7	7.06	30	1
7	2	28	140	9.33	4.38	19	0	0.55	0.28	0.93	0.00	57	5.4	9.57	30	1
8	7	23	50	3.33	2.74	9	0	0.22	0.35	1.00	0.00	182	14.02	13.58	30	1
9	3	27	81	5.4	2.95	12	0	0.35	0.34	1.00	0.00	84	7.78	10.92	30	1
10	1	29	110	7.33	3.72	14	0	0.47	0.39	1.00	0.00	29	4.1	6.99	30	1
11	3	27	111	7.4	3.67	13	0	0.5	0.35	1.00	0.00	84	7.37	11.09	30	1
12	1	29	106	7.07	4.13	18	0	0.59	0.36	1.00	0.00	29	3.93	7.01	30	1
13	1	29	147	9.8	4.37	20	0	0.59	0.24	0.9	0.00	29	3.56	7.09	30	1
14	4	26	114	7.6	5.44	17	0	0.39	0.37	0.93	0.00	110	8.98	12.25	30	1
15	1	29	125	8.33	4.6	20	0	0.38	0.29	1.00	0.00	29	3.74	7.05	30	1
16	3	27	103	6.87	3.5	13	0	0.34	0.26	0.87	0.00	84	7.34	11.1	30	1
17	5	25	89	5.93	3.77	13	0	0.43	0.34	1.00	0.00	135	10.66	12.99	30	1
18	1	29	124	8.27	3.38	13	0	0.45	0.31	1.00	0.00	29	3.82	7.03	30	1
19	0	30	106	7.07	3.38	15	2	0.54	0.27	1.00	0.00	0	2.16	0.89	5	1
20	1	29	117	7.8	4.13	17	0	0.5	0.28	1.00	0.00	29	3.77	7.04	30	1

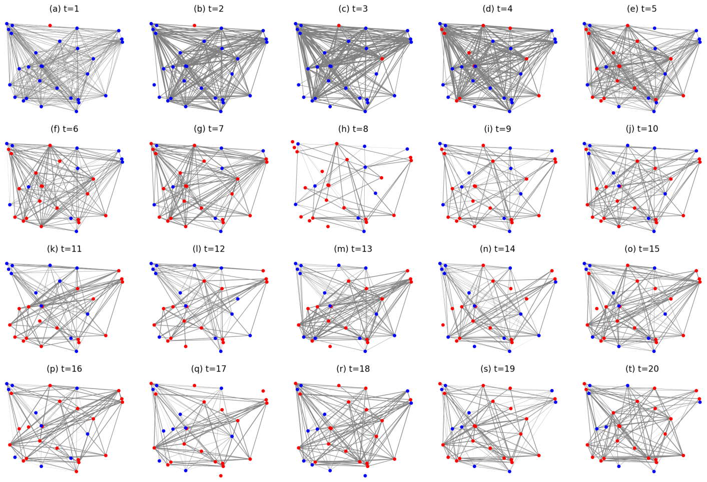

Fig C. The evolving social networks driven by egocentric nodes in an epidemic outbreak.

Cooperative

Table D. Topological information of the network simulations driven by cooperative mutation style under a social capital limit at 20.

Iteration	Nodes		Edges	Node Degree				Clustering coefficient				Shortest path length				
	Connected	Unconnected		Avg.	Std.	Max.	Min.	Avg.	Std.	Max.	Min.	Fake Paths	Avg.	Std.	Max.	Min.
0	30	0	0	0.00	0.00	0	0	0.00	0.00	0	0	435	30.00	0.00	30	30
1	1	29	268	17.87	3.73	20	0	0.63	0.12	0.71	0.00	29	3.25	7.16	30	1
2	3	27	219	14.6	7.17	20	0	0.66	0.27	1.00	0.00	84	6.93	11.3	30	1
3	0	30	243	16.2	5.42	20	1	0.64	0.21	0.84	0.00	0	1.49	0.58	3	1
4	1	29	227	15.13	6.68	20	0	0.61	0.27	0.84	0.00	29	3.43	7.13	30	1
5	0	30	240	16.0	5.61	20	1	0.6	0.25	0.84	0.00	0	1.49	0.58	3	1
6	1	29	234	15.6	6.23	20	0	0.59	0.28	0.85	0.00	29	3.37	7.14	30	1
7	1	29	240	16.0	5.68	20	0	0.6	0.25	0.84	0.00	29	3.34	7.15	30	1
8	1	29	232	15.47	6.18	20	0	0.59	0.27	0.83	0.00	29	3.37	7.14	30	1
9	1	29	225	15.0	6.82	20	0	0.67	0.27	0.92	0.00	29	3.41	7.13	30	1
10	0	30	228	15.2	5.74	20	2	0.62	0.17	0.83	0.00	0	1.51	0.56	3	1
11	2	28	225	15.0	6.84	20	0	0.66	0.28	0.89	0.00	57	5.16	9.66	30	1
12	1	29	226	15.07	6.14	20	0	0.62	0.22	0.82	0.00	29	3.37	7.14	30	1
13	1	29	222	14.8	6.97	20	0	0.61	0.31	0.88	0.00	29	3.4	7.13	30	1
14	0	30	226	15.07	6.1	20	1	0.64	0.23	1.00	0.00	0	1.55	0.62	3	1
15	1	29	224	14.93	6.79	20	0	0.62	0.3	0.88	0.00	29	3.42	7.13	30	1
16	0	30	228	15.2	6.02	20	1	0.63	0.22	1.00	0.00	0	1.54	0.61	3	1
17	2	28	226	15.07	6.72	20	0	0.65	0.28	0.91	0.00	57	5.19	9.65	30	1
18	0	30	227	15.13	6.0	20	1	0.64	0.21	1.00	0.00	0	1.57	0.66	4	1
19	1	29	226	15.07	6.41	20	0	0.61	0.28	0.86	0.00	29	3.37	7.14	30	1
20	2	28	224	14.93	6.32	20	0	0.63	0.23	1.00	0.00	57	5.16	9.66	30	1

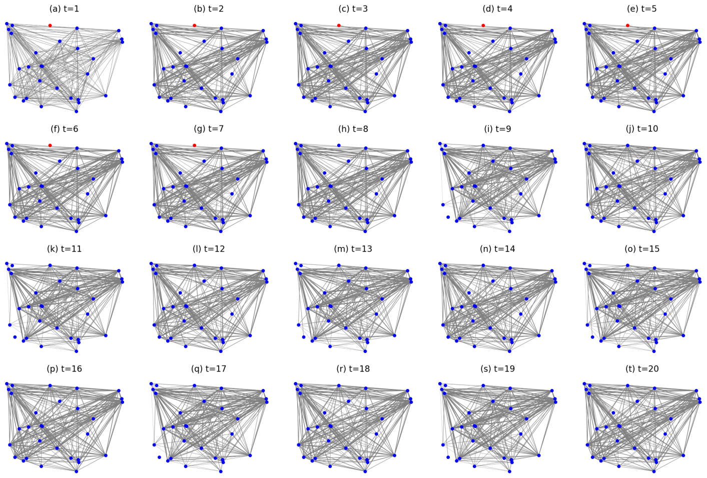

Fig D. The evolving social networks driven by cooperative nodes in an epidemic outbreak.

Collaborative

Table E. Topological information of the network simulations driven by collaborative mutation style under a social capital limit at 20.

Iteration	Nodes		Edges	Node Degree				Clustering coefficient				Shortest path length				
	Connected	Unconnected		Avg.	Std.	Max.	Min.	Avg.	Std.	Max.	Min.	Fake Paths	Avg.	Std.	Max.	Min.
0	30	0	0	0.00	0.00	0	0	0.00	0.00	0	0	435	30.00	0.00	30	30
1	1	29	356	23.73	4.85	27	0	0.85	0.16	0.9	0.00	29	3.05	7.21	30	1
2	0	30	323	21.53	5.71	29	1	0.81	0.16	0.95	0.00	0	1.26	0.44	2	1
3	0	30	329	21.93	5.88	29	1	0.83	0.17	0.98	0.00	0	1.24	0.43	2	1
4	0	30	325	21.67	5.89	29	1	0.83	0.16	0.94	0.00	0	1.25	0.43	2	1
5	0	30	329	21.93	6.07	29	1	0.84	0.17	1.00	0.00	0	1.24	0.43	2	1
6	0	30	303	20.2	6.12	29	1	0.8	0.16	1.00	0.00	0	1.3	0.46	2	1
7	0	30	324	21.6	5.87	29	1	0.82	0.16	1.00	0.00	0	1.26	0.44	2	1
8	0	30	319	21.27	5.85	29	1	0.81	0.16	0.95	0.00	0	1.27	0.44	2	1
9	0	30	321	21.4	5.15	29	8	0.82	0.07	0.97	0.71	0	1.26	0.44	2	1
10	0	30	339	22.6	4.25	29	12	0.83	0.04	0.94	0.76	0	1.22	0.41	2	1
11	0	30	324	21.6	4.74	29	8	0.8	0.06	0.9	0.7	0	1.26	0.44	2	1
12	0	30	350	23.33	3.82	29	13	0.84	0.04	0.94	0.78	0	1.2	0.4	2	1
13	0	30	342	22.8	4.36	29	10	0.84	0.05	0.94	0.76	0	1.21	0.41	2	1
14	0	30	322	21.47	5.21	29	8	0.82	0.07	0.94	0.71	0	1.26	0.44	2	1
15	0	30	348	23.2	4.06	29	11	0.84	0.05	0.95	0.78	0	1.2	0.4	2	1
16	0	30	317	21.13	5.26	29	10	0.81	0.08	0.96	0.71	0	1.27	0.44	2	1
17	0	30	356	23.73	3.85	29	15	0.86	0.05	0.94	0.79	0	1.18	0.39	2	1
18	0	30	316	21.07	5.32	29	10	0.81	0.08	0.95	0.68	0	1.27	0.45	2	1
19	0	30	351	23.4	3.83	29	13	0.84	0.04	0.94	0.78	0	1.19	0.39	2	1
20	0	30	321	21.4	5.1	29	9	0.81	0.06	0.93	0.7	0	1.26	0.44	2	1

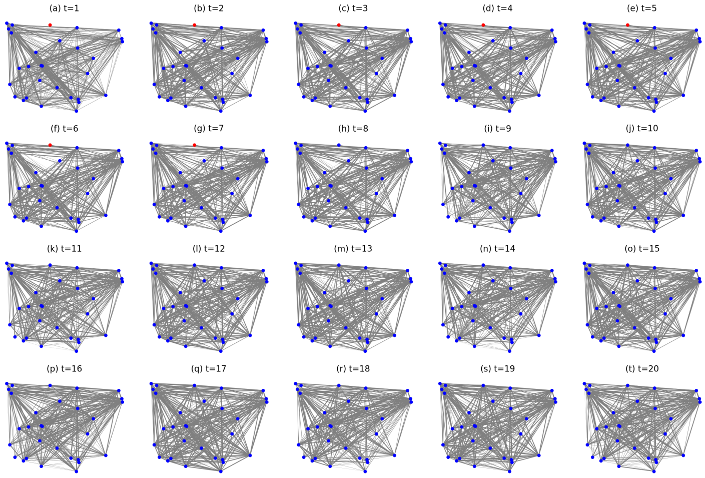

Fig E. The evolving social networks driven by collaborative nodes in an epidemic outbreak.
